# Selection of reference genes for RT‐qPCR normalization in blueberry (*Vaccinium corymbosum* × *angustifolium*) under various abiotic stresses

**DOI:** 10.1002/2211-5463.12903

**Published:** 2020-06-23

**Authors:** Yu Deng, Yadong Li, Haiyue Sun

**Affiliations:** ^1^ College of Life Sciences Jilin Agricultural University Changchun China; ^2^ Engineering Center of Genetic Breeding and Innovative Utilization of Small Fruits of Jilin Province College of Horticulture Jilin Agricultural University Changchun China

**Keywords:** abiotic stress, blueberry (*Vaccinium corymbosum* × *angustifolium*), reference gene, RT‐qPCR

## Abstract

As a small fruit rich in anthocyanins, blueberry (*Vaccinium corymbosum* × *angustifolium*) has become a focus of research in recent years for identifying genes related to anthocyanin transport and stress resistance mechanisms based on transcriptome sequencing. However, the lack of validated, stably expressed reference genes greatly limits the functional study of blueberry genes. Therefore, in this study, we selected 14 candidate reference genes from a blueberry transcriptome database and used three algorithms (geNorm, NormFinder and BestKeeper) to evaluate the expression stability of these genes in various organs at different fruit developmental stages under five abiotic stress conditions. *EF1α*, *EIF* and *TBP* were observed to be the most stable and were thus chosen as reference genes for quantitative real‐time PCR. Measurement of the relative expression of *VcMATE1* (European Nucleotide Archive accession number KF875433) in blueberry further verified the reliability of these reference genes, which may have great utility for determining the accuracy of gene expression analyses in future research on blueberry.

## Graphical abstract

A stably expressed reference gene is required for us to understand gene function and stress resistance mechanisms of blueberry. In this study, 14 candidate reference genes were evaluated and validated under natural conditions and various abiotic stresses by three algorithms. Our results may lay the foundation for accurate analysis of blueberry gene expression.

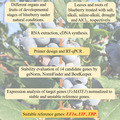

**Abbreviations**
ABA: abscisic acid
*ACE*: *actin*
Com: Comprehensive ranking
C*t*: cycle threshold
CTAB: cetyl trimethyl ammonium Bromide
*CYP*: *cyclophilin*
*EF1α*: *elongbation factor 1‐alpha 3*
*EIF*: *eukaryotic initiation factor 4A*
*Fbox*: *F‐box family protein*
*FLD*: *flowering locus D*
*GAPDH*: *glyceraldehyde‐3‐phosphate dehydrogenase*
*HIS*: *histone*
HKG: housekeeping gene
Nr database: RefSeq nonredundant proteins database
PEG: poly(ethylene glycol)
*PP2A*: *protein phosphatase 2A regulatory subunit*
*RP*: *RNA polymerase subunit*
RPKM: reads per kilobase per million mapped reads
RT‐qPCR: quantitative real‐time PCR
*S*: stability
*SAND*: *SAND family protein*
SD: standard deviation
TARAAPE: transcripts more prevalent in the sarcocarp library
*TBP*: *TATA‐box binding protein*
TBRAAPE: transcripts more prevalent in the exocarp library
*TUB*: *β‐Tubulin*
*UBCE*: *ubiquitin conjugating enzyme2*

Plants are continuously exposed to various ambient conditions that can cause detrimental effects during all developmental stages [1, 2]. Abiotic stresses are nonbiological factors that influence living organisms in a specific environment that negatively affects the growth and yields of crop [3]. High salinity [2, 4], drought [4, 5], flooding [6, 7], chilling [8, 9, 10], heat [4, 10] and heavy metal stresses [11, 12, 13, 14, 15, 16] are adverse natural factors that affect fruit tree growth and yield that are undesirable to be encountered during the whole process. Shielding plants from high salinity, drought and other abiotic stresses is difficult. Plants have evolved strategies to cope with such stresses, and elucidation of the underlying mechanisms is thus increasingly useful [2, 5]. Researchers have found that abiotic stress‐induced gene expression is an efficient, valuable tool for perceiving slight changes in the expression levels of worthwhile target genes [5].

In conjunction with advances in scientific research in multiple disciplines, a variety of methods have been developed for use in gene expression analysis, such as RNA sequencing, serial analysis of gene expression, RNase protection assays, microarrays and northern blotting. Three main techniques are currently used to detect differential gene expression induced by various abiotic stress conditions: northern blotting, microarrays and quantitative real‐time PCR (RT‐qPCR) [17]. But the uncontrollability of the quality of RNA and the low‐throughput nature of northern blotting limit the accuracy of the determination of expression levels. Because northern blotting is time‐consuming and labor intensive, it has been largely superseded by microarrays and RT‐qPCR [17]. Although the ability of microarray platforms to handle thousands of gene probes is attractive, this method does not have the sensitivity needed to detect variation in gene expression or the specificity exhibited by probe hybridization [18, 19, 20].

RT‐qPCR, which has the highest sensitivity and accuracy of different methods for detecting gene expression changes, uses probes or fluorescent dyes to determine the quantity of the initial template [21]. Similar to DNA microarray hybridization, RT‐qPCR can be used to screen multiple target genes simultaneously. Furthermore, the nature of the PCR leads to a high detection sensitivity and high specificity, thereby allowing gene expression levels to be measured. Such characteristics make RT‐qPCR an attractive tool for better studying gene expression changes and regulatory mechanisms induced by abiotic stresses in plants [22].

Because of the earlier‐mentioned benefits, we used RT‐qPCR based on the SYBR Green I method in this study. Although RT‐qPCR is powerful and sensitive, another indispensable factor, reference genes, must also be considered. Reference genes are internal controls whose expressions in various species, organs, cells and environments are relatively constant. During detection of gene expression level changes, reference genes can be used to correct for sample size and experimental errors. Although both functional and nonfunctional genes are feasible choices as reference genes, housekeeping genes (HKGs) are the most frequently used because their expression levels are less influenced by ambient factors, and they are consistently and stably expressed in almost all organs and during all growth phases. HKGs have minimal influence on RNA quality and the efficiency of reverse transcription, and normalization to HKGs increases the reliability of RT‐qPCR results. Recent studies have shown that a suitable HKG should be carefully selected because no HKG is applicable to all conditions. In this study, we therefore aimed to select a suitable reference gene for the standardization of gene expression in blueberry [17].

Blueberry is a deciduous or evergreen, perennial shrub in *Vaccinium* (Ericaceae), a genus that also includes cranberry and lingonberry [23]. Blueberry is of interest because of its valuable, succulent, attractive fruits, which contain a high level of anthocyanins [24]. In addition, commercial blueberry cultivation is becoming more popular among growers. To better understand blueberry physiology and gene functions, we chose a transcriptomic approach, which requires precise quantification of expression abundance. RT‐qPCR is an accurate, stable and efficient method for the detection of transcript abundance. Gene expression data obtained by RT‐qPCR must be normalized using validated, stably expressed reference genes. The evaluation and identification of suitable reference genes were thus important for our study of blueberry. Various abiotic stresses seriously affect fruit yield [4, 10, 15, 25]. We therefore aimed to study gene expression in blueberry under abiotic stress, which required the identification of a stable reference gene. On the basis of literature reports, we chose the following 14 reference genes as candidates for standardization of a target gene and tested their expression stability: *actin* (*ACT*), *cyclophilin* (*CYP*), *elongation factor 1‐alpha 3* (*EF1α*), *eukaryotic initiation factor* *4A* (*EIF*), *F‐box family protein* (*Fbox*), *flowering locus D* (*FLD*), *glyceraldehyde‐3‐phosphate dehydrogenase* (*GAPDH*), *histone* (*HIS*), *protein phosphatase* *2A* *regulatory subunit* (*PP2A*), *RNA polymerase subunit* (*RP*), *SAND family protein* (*SAND*), *TATA‐box binding protein* (*TBP*), *β‐Tubulin* (*TUB*) and *ubiquitin conjugating enzyme2* (*UBCE*) [17]. Our search for a stable internal reference gene was aimed to lay a foundation for future studies of gene expression in blueberry.

## Materials and methods

### Plant materials, growth conditions and abiotic treatments

The half‐high blueberry cultivar ‘Northland’ was used during the 2018 season in this study. We collected roots, stems, leaves, leaf buds and flower buds from fresh, tender parts. Flowers were gathered at the full‐bloom phase, and green, pink and blue fruits were, respectively, collected 24, 42 and 54 days after flowering. Seeds, exocarps and sarcocarps were separated from blue fruits after collection. Samples collection location was Engineering Center of Genetic Breeding and Innovative Utilization of Small Fruits of Jilin Province, Changchun, China.

For abiotic stress treatments, samples were collected from ~1000 two‐year‐old seedlings of ‘Northland’ grown at the farm of Tonghua Heyun Modern Agricultural Co. in Tonghua, China, during the 2018 season. The plants were then cultured in 4 L Hoagland’s nutrient solution (pH 4.5–5.0) and grown in a controlled climate chamber (25 °C/22 °C day/night temperature, 16‐h/8‐h photoperiod, 100 μmoL·m^−2^·s^−1^ photon flux density and 40–60% relative humidity). The roots of the blueberry were cleaned before placing into Hoagland’s nutrient solution. All samples in nonstress and stress treatments were oxygenated for 2 h twice daily, with the Hoagland’s nutrient solution replaced at regular intervals. After treatment, plant materials were immediately collected in pre‐prepared aluminum foil parcels, quickly frozen in liquid nitrogen and then stored in an ultracold storage freezer at −80 °C until milling for total RNA isolation. The whole process was performed rapidly to prevent sample thawing.

Prior to abiotic treatments, we precultured the plants in the climate chamber for 10 days. The following stress treatments were applied: salt treatment (110 mm NaCl), alkaline treatment (110 mm NaHCO_3_), saline–alkaline treatment (50 mm NaCl and 70 mm NaHCO_3_), drought treatment [8% poly(ethylene glycol) (PEG) 8000] and AlCl_3_ treatment (100 μm AlCl_3_). At various time points during the different stress treatments (0, 2, 6, 12 and 24 h), root and leaf samples were separately collected, with at least three biological repeats, and frozen in liquid nitrogen for expression analyses (Fig. S1; Table S1).

### RNA extraction, DNase treatment and cDNA synthesis

Total RNA was extracted using a modified cetyl trimethyl ammonium bromide method [26, 27]. The quantity and quality of extracted RNA were determined on an IMPLEN P330 instrument. Only RNA samples meeting the following criteria were used in this study: (a) absorbance (*A*) ratios within a certain range, namely, 1.8 ≤ *A*_260_/*A*_280_ ≤ 2.0; (b) *A*_260_/*A*_230_ approximately equal to 2.0; and (c) 28S/18S ribosomal RNA bands clear and distinct, with no smearing on 1.2% (w/v) agarose gels. To ensure the consistency of each individual reaction, we synthesized cDNA in 20‐µL volumes containing 1000 ng template RNA using a PrimeScript RT reagent kit with gDNA Eraser (Perfect Real Time, Takara, Japan) according to the kit protocol. The gDNA Eraser in the reagent kit was able to effectively remove DNA in the total RNA. All cDNAs were stored at −20 °C until use.

### Selection of candidate reference genes

The 14 candidate reference genes were evaluated. These genes were chosen based on their previous use in blueberry and other popular species, including *ACT*, *CYP*, *EF1α*, *EIF*, *Fbox*, *FLD*, *GAPDH*, *HIS*, *PP2A*, *RP*, *SAND*, *TBP*, *TUB* and *UBCE*. Because blueberry genomic information is lacking, we had previously generated a transcriptome from blueberry exocarps and sarcocarps by Illumina sequencing technology. After assembly and annotation using SOAPdenovo, expression profile data of each organ were mapped to the transcriptome. The Genome Analyzer IIX platform was used to convert unigene reads per kilobase per million mapped reads (FPKM values). The following statistics were obtained for differentially expressed genes: gene ID, gene expression level, gene description and the differential expression relationship, log_2_ (TBRAAPE_RPKM/TARAAPE_RPKM). Values of false discovery rate ≤0.001 and |log_2_ (TBRAAPE_RPKM/TARAAPE_RPKM)| ≥ 1 were used as the criteria for judging the significance of gene expression differences [28].

Using the earlier transcriptome, we also selected several HKGs as candidate genes according to the results of previous studies. Genes meeting the following criteria were considered to be candidate reference genes: protein annotated in the RefSeq non‐redundant proteins database and |log_2_ (TBRAAPE_RPKM/TARAAPE_RPKM) | < 0.6.

### Primer design and validation of candidate genes

Primers were designed with primer premier 5 software (PREMIER, North York, ON, USA) and the Primer‐BLAST online tool according to the following criteria: primer length of ~18–30 bp, GC content 40–60%, melting temperature 58–62 °C and amplicon length of 100–150 bp. All RT‐qPCR primers were synthesized by Suzhou Genewiz Bio‐Technology Services Co. (Suzhou, China).

High‐quality amplification efficiency is a prerequisite for reliable RT‐qPCR results. After even mixing, cDNA was diluted by 5‐fold gradient dilution (5^0^, 5^−1^, 5^−2^, 5^−3^ and 5^−4^). Calibration curves were automatically generated by the StepOne Plus system software, and PCR amplification efficiency was automatically calculated according to the formula *E* = 10^(−1/slope)^ − 1. To ensure high specificity and efficiency of primers during RT‐qPCR amplification, we used only primers with an amplification efficiency near 100% and a correlation coefficient (*R* ^2^) >0.99. The presence of a single peak in the melting curve was required to further confirm the amplification specificity of the mRNA of a candidate reference gene.

### RT‐qPCR and data analyses

RT‐qPCR was performed in 96‐well plates on an Applied Biosystems StepOne Plus Realtime PCR system (Thermo Fisher, Waltham, MA, USA), with the ratio of components in each 20‐µL reaction mixture conforming to the specifications of a TB Green Premix Ex Taq II kit (Tli RNaseH Plus; Takara). The following cycling protocol was used: 40 cycles of 95 °C for 30 s, 95 °C for 5 s and 60 °C for 30 s, followed by 95 °C for 10 s, 60 °C for 60 s and 95 °C for 15 s to generate the melting curve. After program completion, background‐corrected fluorescence data and cycle threshold (C*_t_*) values were immediately calculated, as well as output by the instrument software. To confirm primer specificity, we checked the RT‐qPCR products by 1% (w/v) agarose gel electrophoresis.

### Analysis of candidate reference‐gene expression stability

C*_t_* values of three replicates, output by the earlier‐mentioned software, were averaged, and the relative expression level (*Q*) of each analyzed gene was calculated using the formulas $Q=2^{-\Delta C_{t}}$ and $Q=2^{-\Delta \Delta C_{t}}$.

To assess the feasibility of candidate reference genes, we analyzed the generated data in geNorm [29], NormFinder [30] and BestKeeper [31]. geNorm was used to obtain *M*‐values for each candidate gene, as well as the optimal number of reference genes, the latter based on the average pairwise variation (*V_n_*_/_ *_n_*_+1_). Using geNorm, we ranked candidate genes according to their expression stability by calculating their *M*‐values, which are inversely proportional to their stability. The default value of *V_n_*_/_ *_n_*_+1_, which was slightly adjustable, was 0.15. If *V_n_*_/_ *_n_*_+1_ was no more than 0.15, the optimal number of reference genes was *n*. If the value of *V_n_*_/_ *_n_*_+1_ was greater than 0.15, the optimal number was expected to be *n* + 1. NormFinder was used to obtain stability (*S*) values and the optimal intergroup gene combination. BestKeeper was also used to analyze the stability of candidate reference genes and additionally used to directly calculate average C*_t_* values. Because BestKeeper can analyze only 10 genes at a time, we removed the four worst genes as determined by NormFinder and geNorm. Finally, geometric means of the results of the three algorithms were combined to obtain a consensus ranking of candidate reference genes.

### Validation of reference genes

*VcMATE1* (European Nucleotide Archive accession number KF875433) was previously cloned in our laboratory [32]. The forward and reverse primer sequences used for RT‐qPCR were 5′‐TGCTTCCATGGCTACCTCCTT‐3′ and 5′‐TTTTGCTCCATAGGACTGCCC‐3′, respectively. Several abiotic stress conditions, mentioned earlier, were chosen for validation of stable and unstable reference genes. To normalize the expression level of *VcMATE1*, we used the reference genes most and least stably expressed under various conditions, namely, the most stable in organs and colored fruits at different periods of maturity (*EIF*, *EF1α*), leaves and roots under conditions of salinity (*PP2A*, *TBP*), alkaline stress (*EIF*, *UBCE*), saline–alkaline conditions (*PP2A*, *HIS*), simulated drought (*TBP*, *GAPDH*) and exposed to AlCl_3_ (*TBP*, *EF1α*), and the least stable under nonstress conditions (*ACT*, *CYP*) and exposure to salinity (*CYP*, *SAND*), alkalinity (*GAPDH*, *CYP*), saline–alkaline (*CYP*, *SAND*), AlCl_3_ (*SAND*, *UBCE*) and simulated drought (*SAND*, *ACT*).

## Results

### Screening for universal candidate reference genes

As shown in Table S2, we evaluated the expression stabilities of all transcripts and removed those transcripts lacking a credible function annotation. Using combined information from the transcriptome database and previous reports, we ultimately selected 14 candidate genes: *ACT* (unigene 2464), *CYP* (unigene 13197), *EF1α* (unigene 12271), *EIF* (unigene 19256), *Fbox* (unigene 7226), *FLD* (unigene 28351), *GAPDH* (unigene 17625), *HIS* (unigene 8208), *PP2A* (unigene 14576), *RP* (unigene 15023), *SAND* (unigene 12206), *TBP* (unigene 11381), *TUB* (unigene 4780) and *UBCE* (unigene 4251) (Table 1).

**Table 1:** Description of the candidate reference genes. KEGG, Kyoto Encyclopedia of Genes and Genomes.

Gene symbol	Target sequence	Nr Description	Nr ID	KEGG Orthology	Gene length (bp)	TARAAPE_RPKM	TBRAAPE_RPKM	Log_2_ ratio
*ACT*	Unigene 2464	Actin (*Populus trichocarpa*)	gi|224088196|ref|XP_002308365.1|	K10355	585	59.346	41.9122	−0.50178
*CYP*	Unigene 13197	Cyclophilin (*Ziziphus jujuba*)	gi|196166898|gb|ACG70968.1|	K01802	495	40.5789	40.4744	−0.00371
*EF1α*	Unigene 12271	Elongation factor 1‐alpha 3 (*Lilium longiflorum*)	gi|5917747|gb|AAD56020.1|AF181492_1	K03231	258	685.0639	830.3061	0.27740
*EIF*	Unigene 19256	Eukaryotic initiation factor 4A‐14 (*Nicotiana tabacum*)	gi|2500520|sp|Q40467.1|IF414_TOBAC	K03257	123	250.9837	239.3225	−0.06918
*Fbox*	Unigene 7226	F‐box protein family (*Arabidopsis lyrata* subsp. *lyrata*)	gi|297806791|ref|XP_002871279.1|	K10102	609	9.1991	8.8816	−0.05067
*FLD*	Unigene 28351	Flowering locus D (*Arabidopsis thaliana*)	gi|240255318|ref|NP_187650.4|	K11450	453	3.4496	3.3721	−0.03278
*GAPDH*	Unigene 17625	Glyceraldehyde‐3‐phosphate dehydrogenase (*Magnolia quinquepeta*)	gi|120669|sp|P26518.1|G3PC_MAGLI	K00134	582	596.6472	656.4584	0.13783
*HIS*	Unigene 8208	Histone H3.2 (*Arabidopsis* *thaliana*)	gi|153799895|gb|ABS50666.1|	K11253	468	153.1042	143.9564	0.28304
*PP2A*	Unigene 14576	Protein phosphatase 2A regulatory subunit B (*Arabidopsis thaliana*)	gi|75274192|sp|Q9LU89.1|2A5N_ARATH	K11584	1518	30.1756	31.5648	0.06493
*RP*	Unigene 15023	RNA polymerase subunit (*Medicago truncatula*)	gi|124359979|gb|ABN07995.1|	K03013	723	75.1492	51.1261	−0.55570
*SAND*	Unigene 12206	SAND family protein (*Arabidopsis lyrata* subsp.* lyrata*)	gi|297822433|ref|XP_002879099.1|	K20195	1890	29.2467	27.7054	−0.18620
*TBP*	Unigene 11381	TATA‐box binding protein (*Phaseolus vulgaris*)	gi|4102725|gb|AAD10238.1|	K03120	606	37.4796	41.8497	0.15911
*TUB*	Unigene 4780	beta‐Tubulin (*Eucalyptus grandis*)	gi|153799895|gb|ABS50666.1|	K07375	1125	15.5758	15.5318	−0.00408
*UBCE*	Unigene 4251	Ubiquitin conjugating enzyme2‐like (*Solanum tuberosum*)	gi|213494485|gb|ACJ48964.1|	K06689	444	236.2412	245.37	0.05470

RNA was quantified, and we verified the integrity of the RNA. The 28S RNA band on the agarose gel was approximately two times brighter than that of 18S RNA (Fig. S2; Table S3).

RT‐qPCR using a 5‐fold serially diluted template yielded amplification products with high efficiency and specificity. The locations of primer pairs on transcript sequences were shown in Data S1. Primer specificity was confirmed by the presence of a single peak in melting curves (Table S4). Amplification efficiencies of candidate reference gene primers varied between 94.312% and 103.908%, and standard curve correlation coefficients ranged from 0.990 to 0.999 (Table 2). RT‐qPCR products were evaluated by 1.0% (w/v) agarose gel electrophoresis and sequencing. Each lane on the agarose gels contained only one band, and the sequencing results confirmed that the expected products were generated (Fig. S3).

**Table 2:** List of primer sequences and related information for 14 candidate reference genes.

Gene symbol	Target sequence	Gene description	Primer sequence (5′–3′) (forward/reverse)	*R* ^2^	Amplification efficiency (%)	Amplicon Tm (°C)	Amplicon length (bp)
*ACT*	Unigene 2464	Actin	GAAATAACAGCGTTGGCCCC	0.997	96.014	83.92	112
			GGAAGGTACTGAGGGATGCG				
*CYP*	Unigene 13197	Cyclophilin	TATTTGCTGATACCACGCCCA	0.990	100.106	84.07	101
			CCCTTTGTAGTGCAATGGCTTC				
*EF1α*	Unigene 12271	Elongation factor‐1 alpha 3	TGGAAATGGGTATGCCCCAG	0.999	97.624	83.47	147
			ACCATACCGGCATCTCCATTC				
*EIF*	Unigene 19256	Eukaryotic initiation factor 4A	GGAGGAAAGGTGTTGCCATCA	0.999	99.905	81.99	117
			GGAGATCAGCAACGTTTGCTG				
*Fbox*	Unigene 7226	F‐box family protein	CGATTCAAGAGCGTGTCAAAGC	0.997	96.744	83.19	109
			AATGCAAACCTGAGACGGTGG				
*FLD*	Unigene 28351	Flowering locus D	GAGTGAAGCTGGTTGGGAGAA	0.997	94.312	83.77	100
			GAAGTTGAAGCAGACTTGCGG				
*GAPDH*	Unigene 17625	Glyceraldehyde‐3‐phosphate dehydrogenase	CCGGAGCTGAGTTTGTTGTT	0.998	95.161	82.58	105
			GACCACCTTCTTTGCACCAC				
*HIS*	Unigene 8208	Histone	AGGAGTCAAGAAGCCCCACA	0.999	96.045	81.70	127
			AGCAATCTCACGAACAAGCC				
*PP2A*	Unigene 14576	Protein phosphatase 2A regulatory subunit	TTCCTGAGATTCGTGGCATCA	0.992	98.785	81.25	103
			CCTCGGAATCGAAAAGATCCA				
*RP*	Unigene 15023	RNA polymerase subunit	GACGAAGGTAGCACCGAGAG	0.996	95.203	79.9	142
			GTGTTTGGCCGTGAATGGAC				
*SAND*	Unigene 12206	SAND family protein	CACCCGAATTCCACTTCAATTG	0.993	97.808	83.63	101
			GGATTATCGGATGCAAGGTCG				
*TBP*	Unigene 11381	TATA‐box binding protein	GCCAACCGGTGGATCTTTCTA	0.991	103.908	80.79	108
			GTGCAATGGCCTTAAGTTCCAA				
*TUB*	Unigene 4780	Beta‐tubulin	CCCCGATAACTTCGTGTTTGG	0.995	100.983	83.48	101
			CGACATCGAGAACCGAATCAAT				
*UBCE*	Unigene 4251	Ubiquitin conjugating enzyme2	CAAACCCCGATGATCCTCTTG	0.990	101.35	83.63	101

### Expression profiles of candidate reference genes

**Fig. 1:**
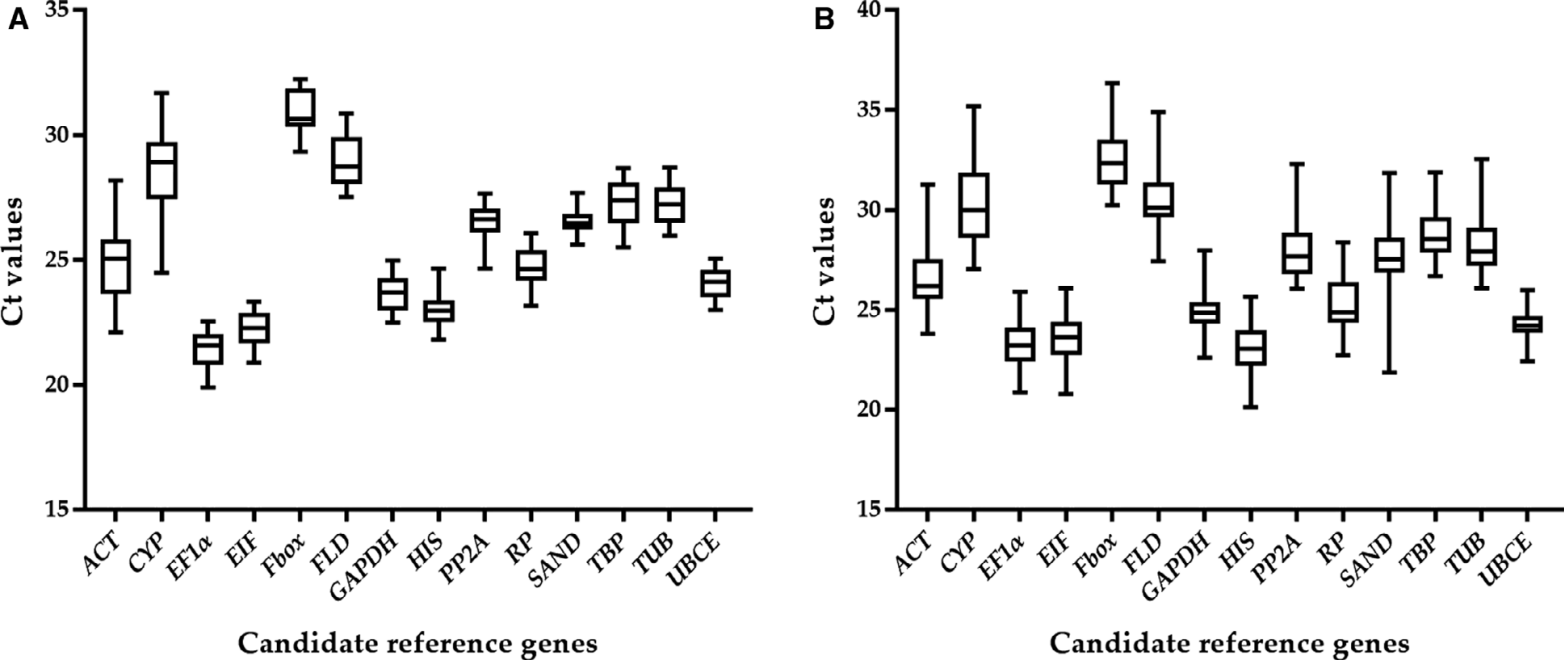
RT‐qPCR C*_t_* values of the candidate reference genes. (A) Candidate reference genes were analyzed in all organ samples. (B) Candidate reference genes were analyzed in leaf and root samples under five abiotic stresses. The box indicates the 25th and 75th percentiles. A line in the box represents the median. Whiskers represent the maximum and minimum values.

Under a nonstress condition, C*_t_* values of candidate genes varied from 19.88 to 32.34 in all test samples, with most values ranging between 22.54 and 29.41. The average C*_t_* values of *CYP*, *Fbox* and *FLD* were 31.68, 32.25 and 30.86, respectively, which indicates that their expressions were weak and their transcript abundances were low. Among the studied reference genes, genes with high expression variation (>6 cycles) were *ACT* and *CYP* (6.10 and 7.20 cycles, respectively). The remaining candidate reference genes had low expression variation (<4 cycles), which ranged from 2.04 to 3.34 cycles (Fig. 1A; Data S2).

Under the different abiotic stress conditions used in this study, C*_t_* values of the 14 genes ranged from 20.80 to 36.36. The majority of C*_t_* values were between 22.23 and 29.55. C*_t_* values of *CYP*, *Fbox* and *FLD* were 35.18, 36.36 and 34.89, respectively. *Fbox*, *PP2A*, *TUB*, *ACT*, *FLD*, *CYP* and *SAND* exhibited high expression variation (>6 cycles, namely, 6.11, 6.22, 6.45, 7.46, 7.49, 8.15 and 9.99, respectively), whereas *UBCE*, *EF1α*, *TBP*, *EIF*, *GAPDH*, *HIS* and *RP* had low expression variation (<6 cycles): 3.56, 5.04, 5.19, 5.27, 5.36, 5.52 and 5.66, respectively (Fig. 1B; Data S3).

The length of the box also provided information about deviations: the shorter the box, the smaller the deviations. Screening for reliable reference genes by various scientific methods was thus necessary to standardize gene expressions under specific conditions in blueberry.

### Analysis of candidate reference‐gene expression stability

#### geNorm analysis

**Fig. 2:**
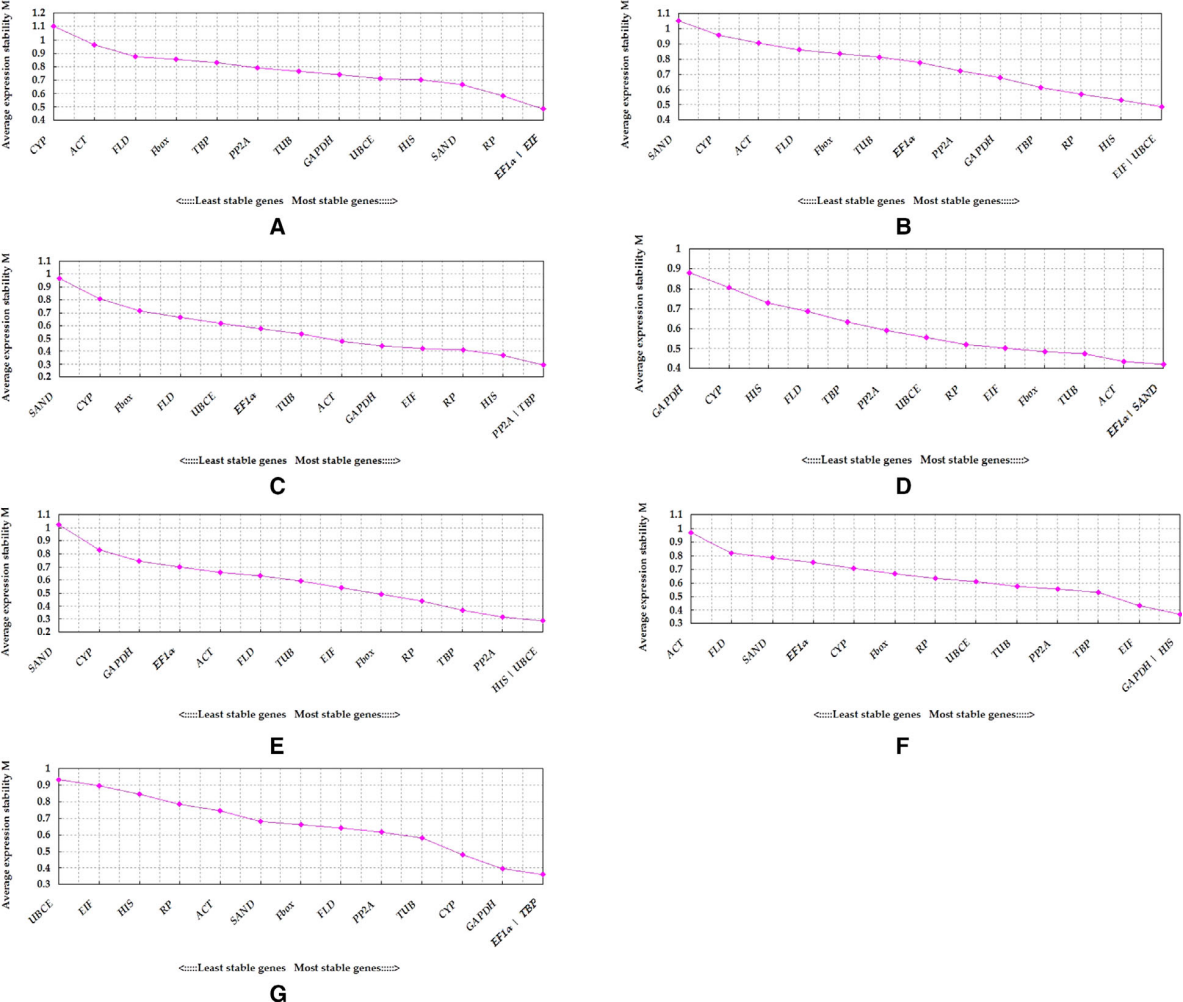
Expression stability and ranking of the candidate reference genes as determined by geNorm. (A) All organ samples without abiotic stresses. (B) All tissue samples under five abiotic stresses. (C) NaCl treatment. (D) NaHCO_3_ treatment. (E) NaCl + NaHCO_3_ treatment. (F) Simulated drought. (G) AlCl_3_ treatment. Average expression stability values (*M*) of the reference genes measured by geNorm. The lower *M*‐value indicated more stable expression level.

**Fig. 3:**
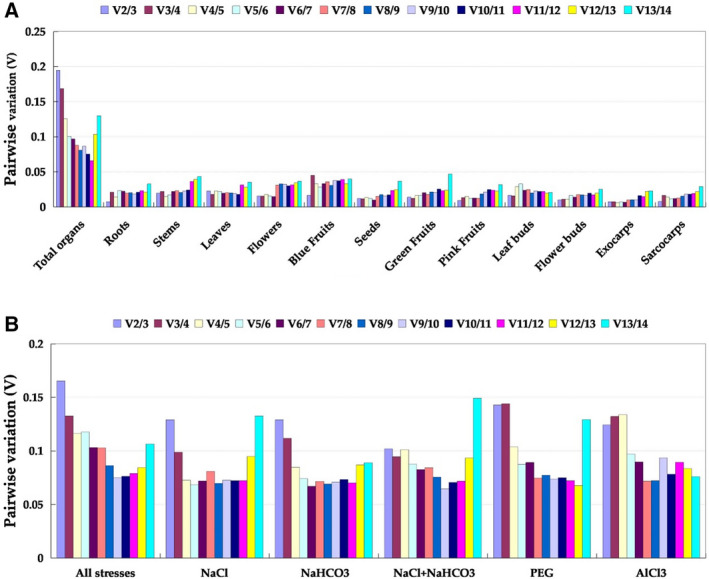
Pairwise variation (*V_n_*/*V_n_*_+1_) values calculated by geNorm. (A) All organ samples without abiotic stresses. (B) All tissue samples under different abiotic stresses. *V_n_*/*V_n_*_+1_ > 0.15 means an additional (*n* + 1) reference was required, whereas *V_n_*/*V_n_*_+1_ ≤ 0.15 means only *n* reference was required.

According to the geNorm analysis, the genes with the smallest *M*‐value (0.485), and thus highest stability, in all organ samples without treatment were *EF1α* and *EIF*. Although *V*_2_/*V*_3_ and *V*_3_/*V*_4_ were both greater than 0.15, *V*_4_/*V*_5_ was smaller than 0.15, which indicated that four genes were needed for normalization of gene expression. The third‐ and fourth‐most stable genes were *RP* (*M* = 0.583) and *SAND* (*M* = 0.667), respectively, and the least stable gene was *CYP* (*M* = 1.101) (Figs 2A and 3A; Table 3).

**Table 3:** Expression stability ranking of 14 candidate reference genes in all organ samples of blueberry without abiotic stresses.

Symbol	All organ samples in blueberry							
	GeNorm		NormFinder		BestKeeper			Com
	*M* ^a^	Rank	*S* ^b^	Rank	*r* ^c^	SD^d^	Rank	
*ACT*	0.965	13	0.878	13	–^e^	–	(11)	12
*CYP*	1.101	14	1.256	14	–	–	(12)	14
*EF1α*	0.485	1	0.328	3	0.773	0.634	2	2
*EIF*	0.485	1	0.271	2	0.855	0.599	1	1
*Fbox*	0.856	11	0.634	12	–	–	(14)	12
*FLD*	0.875	12	0.617	11	–	–	(13)	11
*GAPDH*	0.739	7	0.408	6	0.768	0.707	4	5
*HIS*	0.699	5	0.427	7	0.558	0.555	(10)	7
*PP2A*	0.791	9	0.479	9	0.742	0.673	6	9
*RP*	0.583	3	0.377	5	0.773	0.743	2	3
*SAND*	0.667	4	0.245	1	0.765	0.376	5	3
*TBP*	0.830	10	0.604	10	0.647	0.843	9	10
*TUB*	0.766	8	0.435	8	0.715	0.748	7	8
*UBCE*	0.713	6	0.365	4	0.705	0.501	8	6
Best gene	*EF1α*/*EIF*		*SAND*			*EIF*		*EIF*
Worst gene	*CYP*		*CYP*			*Fbox*		*CYP*
Best combination	*EF1*α/*EIF*/*RP*/*SAND*							

Results without statistical significance were put in parentheses and not used at last.

^a^ *M*: stability values were calculated by geNorm. The lower the *M*‐value is, the more stable is the gene.

^b^ *S*: stability values were calculated by NormFinder. The lower the *S*‐value is, the more stable is the gene.

^c^ *r*: Pearson’s correlation coefficient was calculated by BestKeeper. The higher the *r*‐value is, the more stable is the gene.

^d^ SD: the SD was calculated by BestKeeper. The value of SD should be <1.

^e^ The dashes indicate that the Pearson’s correlation coefficient that was *P* > 0.05 or SD > 1 was deleted.

In both leaf and root samples under all abiotic stresses, the most stable genes were *EIF* and *UBCE* (*M* = 0.487), and the least stable gene was *SAND* (*M* = 1.054). Because *V*_2_/*V*_3_ > 0.15 and *V*_3_/*V*_4_ < 0.15, we needed to add a third gene, *HIS* (*M* = 0.530), to the normalization. The candidate reference genes assessed in our study did not exhibit consistent stability across different sample sets and all situations (Figs 2B and 3B; Table 4). Under high salinity, *PP2A* + *TBP* (*M* = 0.292, *V*_2_/*V*_3_ = 0.129) were ranked as the most stable, whereas *SAND* (*M* = 0.967) was the least stable (Figs 2C and 3B; Table 5). Under high alkalinity, *EIF* + *SAND* (*M* = 0.422, *V*_2_/*V*_3_ = 0.129) and *GAPDH* (*M* = 0.881) were the best and worst reference genes, respectively (Figs 2D and 3B; Table 5). In leaf and root samples subjected to combined salinity and alkalinity stress, *HIS* + *UBCE* (*M* = 0.285, *V*_2_/*V*_3_ = 0.102) and *SAND* (*M* = 1.022) had the highest and lowest stabilities, respectively (Figs 2E and 3B; Table 5). *GAPDH* + *HIS* (*V*_2_/*V*_3_ = 0.143), with an *M*‐value of 0.367, and *ACT*, with an *M*‐value of 0.970, were, respectively, the most and least suitable genes in leaf and root samples under PEG‐simulated drought (Figs 2F and 3B; Table 5). *EF1α* + *TBP* (*M* = 0.359, *V*_2_/*V*_3_ = 0.124) performed best in root samples subjected to the AlCl_3_ treatment, whereas *UBCE* (*M* = 0.933) performed the worst (Figs 2G and 3B; Table 5). *SAND* and *CYP* were the least stably expressed candidate reference genes under the different abiotic stresses in this study (Fig. 2; Tables 4 and 5). These results underscore the importance of screening for the reference genes that are most appropriate for a given set of experimental conditions.

**Table 4:** Expression stability ranking of 14 candidate reference genes in all samples of blueberry under abiotic stresses.

Symbol	All samples under five abiotic stresses							
	GeNorm		NormFinder		BestKeeper			Com
	*M* ^a^	Rank	*S* ^b^	Rank	*r* ^c^	SD^d^	Rank	
*ACT*	0.903	12	0.205	5	–^e^	–	(14)	11
*CYP*	0.958	13	0.307	12	–	–	(11)	13
*EF1α*	0.779	8	0.159	3	0.919	0.997	2	3
*EIF*	0.487	1	0.194	4	0.883	0.909	4	1
*Fbox*	0.833	10	0.243	8	–	–	(8)	9
*FLD*	0.861	11	0.409	14	–	–	(12)	14
*GAPDH*	0.677	6	0.300	10	0.834	0.729	5	7
*HIS*	0.530	3	0.290	9	0.952	0.965	1	3
*PP2A*	0.722	7	0.207	6	–	–	(9)	8
*RP*	0.571	4	0.133	2	–	–	(10)	5
*SAND*	1.054	14	0.230	7	–	–	(13)	12
*TBP*	0.614	5	0.082	1	0.907	0.943	3	1
*TUB*	0.812	9	0.310	13	–	–	(7)	10
*UBCE*	0.487	1	0.304	11	0.746	0.488	6	6
Best gene	*EIF*/*UBCE*		*TBP*			*HIS*		*TBP*/*EIF*
Worst gene	*SAND*		*FLD*			*ACT*		*FLD*
Best combination	*EIF*/*UBCE*/*HIS*		*EIF*/*PP2A* (0.080)					

^a^ *M*: stability values were calculated by geNorm. The lower the *M*‐value is, the more stable is the gene.

^b^ *S*: stability values were calculated by NormFinder. The lower the *S*‐value is, the more stable is the gene.

^c^ *r*: Pearson’s correlation coefficient was calculated by BestKeeper. The higher the *r*‐value is, the more stable is the gene.

^d^ SD: the SD was calculated by BestKeeper. The SD should be <1.

^e^ The dashes indicate that the Pearson’s correlation coefficient that was *P* > 0.05 or SD > 1 was deleted.

**Table 5:** Expression stability comprehensive ranking of 14 candidate reference genes in all organs under common conditions or in both leaves and roots under various abiotic stresses.

Method	1	2	3	4	5	6	7	8	9	10	11	12	13	14
(A) Ranking order under different organs (better‐good‐average)														
geNorm	*EF1α*/*EIF*		*RP*	*SAND*	*HIS*	*UBCE*	*GAPDH*	*TUB*	*PP2A*	*TBP*	*Fbox*	*FLD*	*ACT*	*CYP*
NormFinder	*SAND*	*EIF*	*EF1α*	*UBCE*	*RP*	*GAPDH*	*HIS*	*TUB*	*PP2A*	*TBP*	*FLD*	*Fbox*	*ACT*	*CYP*
BestKeeper	*EIF*	*EF1α*	*RP*	*GAPDH*	*SAND*	*PP2A*	*TUB*	*UBCE*	*TBP*	*HIS*	*ACT*	*CYP*	*FLD*	*Fbox*
Comprehensive ranking	*EIF*	*EF1α*	*RP*	*SAND*	*GAPDH*	*UBCE*	*HIS*	*TUB*	*PP2A*	*TBP*	*FLD*	*Fbox*	*ACT*	*CYP*
(B) Ranking order under all stresses (better‐good‐average)														
geNorm	*EIF/UBCE*		*HIS*	*RP*	*TBP*	*GAPDH*	*PP2A*	*EF1α*	*TUB*	*Fbox*	*FLD*	*ACT*	*CYP*	*SAND*
NormFinder	*TBP*	*RP*	*EF1α*	*EIF*	*ACT*	*PP2A*	*SAND*	*Fbox*	*HIS*	*GAPDH*	*UBCE*	*CYP*	*TUB*	*FLD*
BestKeeper	*HIS*	*EF1α*	*TBP*	*EIF*	*GAPDH*	*UBCE*	*TUB*	*Fbox*	*PP2A*	*RP*	*CYP*	*FLD*	*SAND*	*ACT*
Comprehensive ranking	*EIF*	*TBP*	*EF1α*	*HIS*	*RP*	*UBCE*	*GAPDH*	*PP2A*	*Fbox*	*TUB*	*ACT*	*SAND*	*CYP*	*FLD*
(C) Ranking order under NaCl stress (better‐good‐average)														
geNorm	*PP2A*/*TBP*		*HIS*	*RP*	*EIF*	*GAPDH*	*ACT*	*TUB*	*EF1α*	*UBCE*	*FLD*	*Fbox*	*CYP*	*SAND*
NormFinder	*PP2A*	*GAPDH*	*TBP*	*EIF*	*EF1α*	*RP*	*ACT*	*HIS*	*TUB*	*UBCE*	*FLD*	*Fbox*	*CYP*	*SAND*
BestKeeper	*PP2A*	*FLD*	*GAPDH*	*HIS*	*TBP*	*EF1α*	*EIF*	*Fbox*	*ACT*	*TUB*	*RP*	*CYP*	*SAND*	*UBCE*
Comprehensive ranking	*PP2A*	*TBP*	*GAPDH*	*HIS*	*EIF*	*EF1α*	*RP*	*ACT*	*FLD*	*TUB*	*Fbox*	*UBCE*	*CYP*	*SAND*
(D) Ranking order under NaHCO_3_ stress (better‐good‐average)														
geNorm	*EF1α*/*SAND*		*ACT*	*TUB*	*Fbox*	*EIF*	*RP*	*UBCE*	*PP2A*	*TBP*	*FLD*	*HIS*	*CYP*	*GAPDH*
NormFinder	*EIF*	*UBCE*	*Fbox*	*TUB*	*EF1α*	*RP*	*TBP*	*ACT*	*PP2A*	*SAND*	*FLD*	*HIS*	*CYP*	*GAPDH*
BestKeeper	*UBCE*	*EIF*	*SAND*	*TBP*	*Fbox*	*TUB*	*EF1α*	*HIS*	*GAPDH*	*FLD*	*ACT*	*RP*	*PP2A*	*CYP*
Comprehensive ranking	*EIF*	*UBCE*	*EF1α*	*Fbox*	*SAND*	*TUB*	*TBP*	*ACT*	*RP*	*PP2A*	*FLD*	*HIS*	*GAPDH*	*CYP*
(E) Ranking order under NaCl + NaHCO_3_ stress (better‐good‐average)														
geNorm	*HIS*/*UBCE*		*PP2A*	*TBP*	*RP*	*Fbox*	*EIF*	*TUB*	*FLD*	*ACT*	*EF1α*	*GAPDH*	*CYP*	*SAND*
NormFinder	*PP2A*	*RP*	*UBCE*	*HIS*	*Fbox*	*EF1α*	*EIF*	*TBP*	*ACT*	*TUB*	*GAPDH*	*FLD*	*CYP*	*SAND*
BestKeeper	*TUB*	*PP2A*	*FLD*	*Fbox*	*EIF*	*HIS*	*UBCE*	*EF1α*	*GAPDH*	*RP*	*ACT*	*TBP*	*CYP*	*SAND*
Comprehensive ranking	*PP2A/HIS*		*UBCE*	*Fbox*	*RP*	*EIF*	*TUB*	*FLD*	*TBP*	*EF1α*	*ACT*	*GAPDH*	*CYP*	*SAND*
(F) Ranking order under drought stress (better‐good‐average)														
geNorm	*GAPDH*/*HIS*		*EIF*	*TBP*	*PP2A*	*TUB*	*UBCE*	*RP*	*Fbox*	*CYP*	*EF1α*	*SAND*	*FLD*	*ACT*
NormFinder	*TBP*	*PP2A*	*TUB*	*CYP*	*Fbox*	*GAPDH*	*RP*	*EIF*	*HIS*	*EF1α*	*UBCE*	*FLD*	*SAND*	*ACT*
BestKeeper	*TBP*	*EIF*	*GAPDH*	*RP*	*UBCE*	*TUB*	*Fbox*	*FLD*	*SAND*	*PP2A*	*EF1α*	*HIS*	*CYP*	*ACT*
Comprehensive ranking	*TBP*	*GAPDH*	*EIF*	*TUB*	*PP2A*	*RP*	*Fbox*	*HIS*	*UBCE*	*CYP*	*EF1α*	*FLD*	*SAND*	*ACT*
(G) Ranking order under AlCl_3_ stresses (better‐good‐average)														
geNorm	*EF1α*/*TBP*		*GAPDH*	*CYP*	*TUB*	*PP2A*	*FLD*	*Fbox*	*SAND*	*ACT*	*RP*	*HIS*	*EIF*	*UBCE*
NormFinder	*GAPDH*	*EF1α*	*TBP*	*RP*	*Fbox*	*CYP*	*ACT*	*PP2A*	*TUB*	*FLD*	*EIF*	*HIS*	*UBCE*	*SAND*
BestKeeper	*ACT*	*TBP*	*HIS*	*EF1α*	*RP*	*EIF*	*GAPDH*	*UBCE*	*TUB*	*PP2A*	*FLD*	*SAND*	*CYP*	*Fbox*
Comprehensive ranking	*TBP*	*EF1α*	*GAPDH*	*ACT*	*RP*	*TUB*	*CYP*	*PP2A*	*Fbox*	*HIS*	*FLD*	*EIF*	*SAND*	*UBCE*

#### NormFinder analysis

The most suitable reference gene is the one with the smallest *S*‐value. Calculated *S*‐values and rankings of candidate reference genes are summarized in Tables 3 and 4.

According to NormFinder, the most stably expressed reference gene in all organ samples under nonstress conditions was *SAND* (*S* = 0.245). The most unstable gene was *CYP* (*S* = 1.256; Table 3). *CYP* was calculated to be the worst gene in both geNorm and NormFinder.

In leaves and roots under the five abiotic stresses, *TBP* (*S* = 0.082) and *EIF* + *PP2A* (*S* = 0.080) had the best performance. The worst gene was *FLD* (*S* = 0.409) (Table 4). In leaf and root samples subjected to the NaCl treatment, the best reference genes were *PP2A* (*S* = 0.111) and *GAPDH* + *PP2A* (*S* = 0.072). The most unstable one was *SAND* (0.673) (Table 5). Under NaHCO_3_ treatment conditions, *EIF* (*S* = 0.150) and *Fbox* + *UBCE* (*S* = 0.111) were found to be the most stable for analysis of leaf and root samples. *GAPDH* (0.759) performed the worst (Table 5). In root and leaf samples subjected to combined NaCl–NaHCO_3_ treatment, *PP2A* (*S* = 0.188) and *PP2A* + *RP* (*S* = 0.130) were the best choices, and *SAND* (0.984) was the worst (Table 5). In leaf and root samples under PEG treatment conditions, *TBP* and *EIF* + *TUB* exhibited the most stable expression, with *S*‐values of 0.141 and 0.106, respectively, and the most unstable gene was *ACT* (0.551) (Table 5). In AlCl_3_‐stressed leaf and root samples, *GAPDH* (*S* = 0.130) and *EF1α* + *GAPDH* (*S* = 0.082) displayed the highest stability. The unstable gene was *SAND* (0.661; Table 5). The stability rankings of leaves and roots separately under different stresses analyzed by NormFinder were shown in Table S5.

#### BestKeeper analysis

BestKeeper, designed by Pfaffl *et al*. [31], can be used to analyze both reference and relevant target genes. Expression levels can be analyzed for only 10 HKGs and 10 target genes in 100 samples at a time. After using BestKeeper to calculate the Pearson’s correlation coefficient (*r*), standard deviation (SD) and coefficient of variation between each pair of genes, the magnitudes of these values can be compared to determine the most stable reference genes. In particular, the larger the value of *r* and the smaller the values of SD and the coefficient of variation, the higher is the expression stability. If the SD is >1, the expression of the candidate reference gene is not considered to be stable.

We first excluded candidate genes with SD values greater than 1. In plant organ samples not subjected to any stress treatments, the most stable reference gene was *EIF* (*r* = 0.855), followed by *EF1α* (*r* = 0.773) and *RP* (*r* = 0.773), which were equally good choices, and then *GAPDH* (*r* = 0.765) and *SAND* (*r* = 0.768; Table 3). In leaves and roots under all abiotic stresses, the five most stable genes in descending order were *HIS* (*r* = 0.952), *EF1α* (*r* = 0.919), *TBP* (*r* = 0.907), *EIF* (*r* = 0.883) and *GAPDH* (*r* = 0.834; Table 4). The stability ranking of 14 candidate reference genes for leaves and roots taken separately of blueberry under all five abiotic stresses by BestKeeper were shown in Table S6. The most stable gene in leaves under all stresses was *HIS* (*r* = 0.880). In roots, *TBP* (*r* = 0.970) performed the best under five treatments. When leaves and roots were calculated separately under individual stress conditions, the *P*‐values of all candidate genes were not <0.05, so the results were not included in it.

### Comprehensive ranking

According to the results of three software programs, we ranked all of the 14 candidate reference genes comprehensively. For all organs under common conditions, *EIF* and *EF1α* was the most stable combination. On the contrary, *CYP* and *ACT* were the least stable genes. Under all five kinds of stresses, *EIF* and *TBP* were the most stable genes, and *CYP* and *FLD* were the worst ones in leaves and roots of blueberry. Under NaCl stress, *PP2A*/*TBP* and *CYP*/*SAND* were the best and worst reference genes, respectively. *EIF*/*UBCE* was the most stable combination, and *GAPDH* and *CYP* were the least stable genes under NaHCO_3_ stress. *PP2A*/*HIS* ranked the best order, and *CYP* and *SAND* ranked the worst order under saline–alkaline condition. The most stable reference genes under drought stress were *TBP*/*GAPDH*, and the most unstable genes were *SAND* and *ACT*. The best performed reference genes under AlCl_3_ treatment were *TBP* and *EF1α*. *SAND* and *UBCE* performed the worst (Table 5). The comprehensive ranking for leaves and root taken separately was shown in Table S7.

To validate the candidate reference genes, we chose them from the comprehensive rankings to analyze the relative expression of *VcMATE1* (Table 5).

### Validation of selected reference genes

**Fig. 4:**
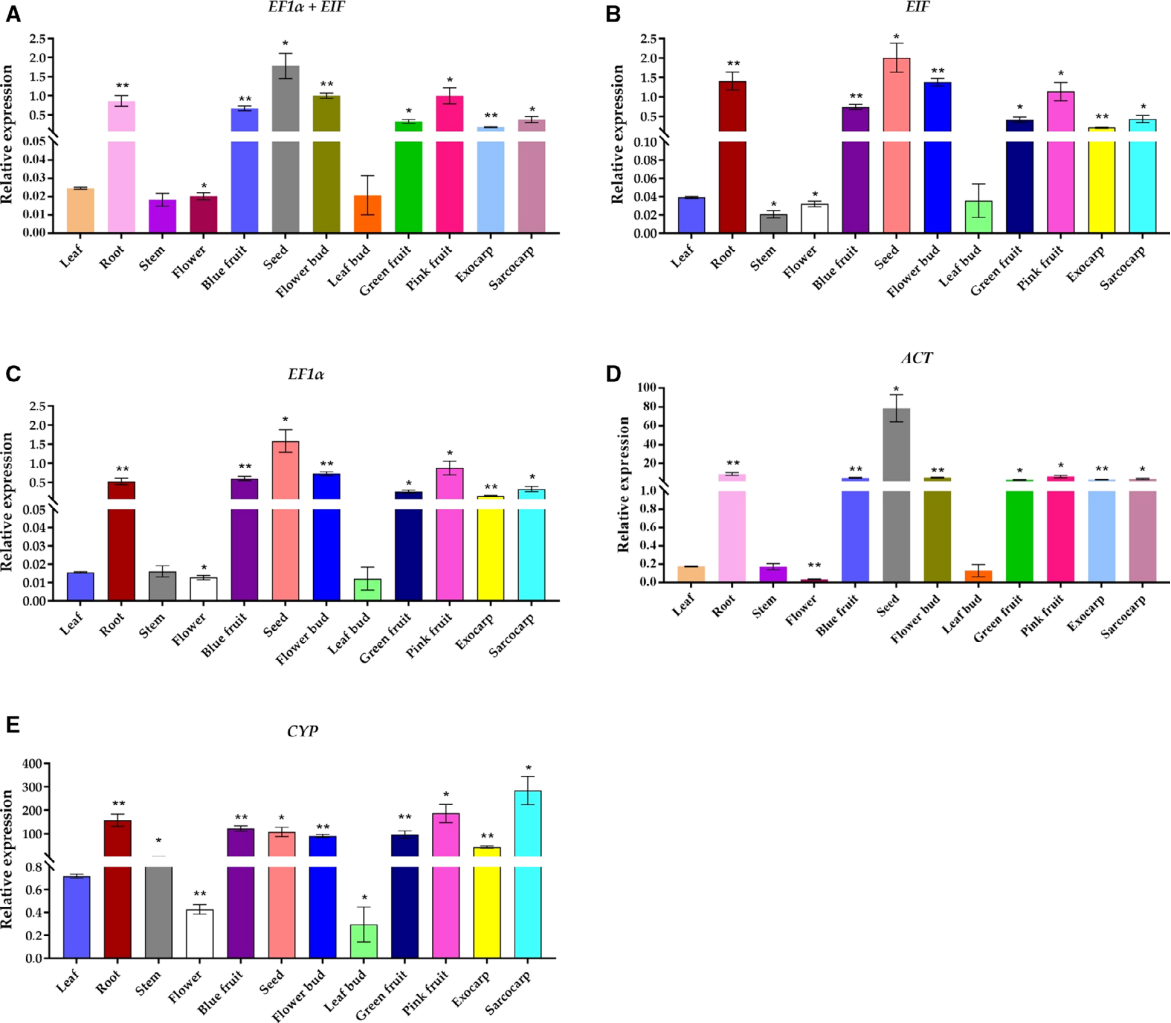
Relative quantification of *VcMATE1* expression using validated reference genes for normalization in different organs. (A) *VcMATE1* normalized by stable reference gene *EF1α* + *EIF*. (B) *VcMATE1* normalized by stable reference gene *EIF*. (C) *VcMATE1* normalized by stable reference gene *EF1α*. (D) *VcMATE1* normalized by unstable reference gene *ACT*. (E) *VcMATE1* normalized by unstable reference gene *CYP*. The error bars represent the SD of three biological replicates. Asterisks indicate that the difference is significant at *P < 0.05 and extremely significant at **P < 0.01, *t*‐test.

To confirm the reliability of our results, we selected the two most stable and two least stable reference genes under different experimental conditions and used them to analyze the relative expression of *VcMATE1* under specific conditions (Fig. 4; Data S4). When the combination of *EIF* + *EF1α* was used as reference genes, the relative expression profiles of *VcMATE1* in different organs and fruit developmental stages were extremely similar to those obtained using *EIF* or *EF1α* as the reference (Fig. 4C). *VcMATE1* expression trends normalized using *ACT* and *CYP*, the two least stable genes, differed from those based on *EIF* and *EF1α*, and the levels of relative expression of *VcMATE1* were extremely high; this was especially true when *CYP* was the reference gene (Fig. 4D,E).

**Fig. 5:**
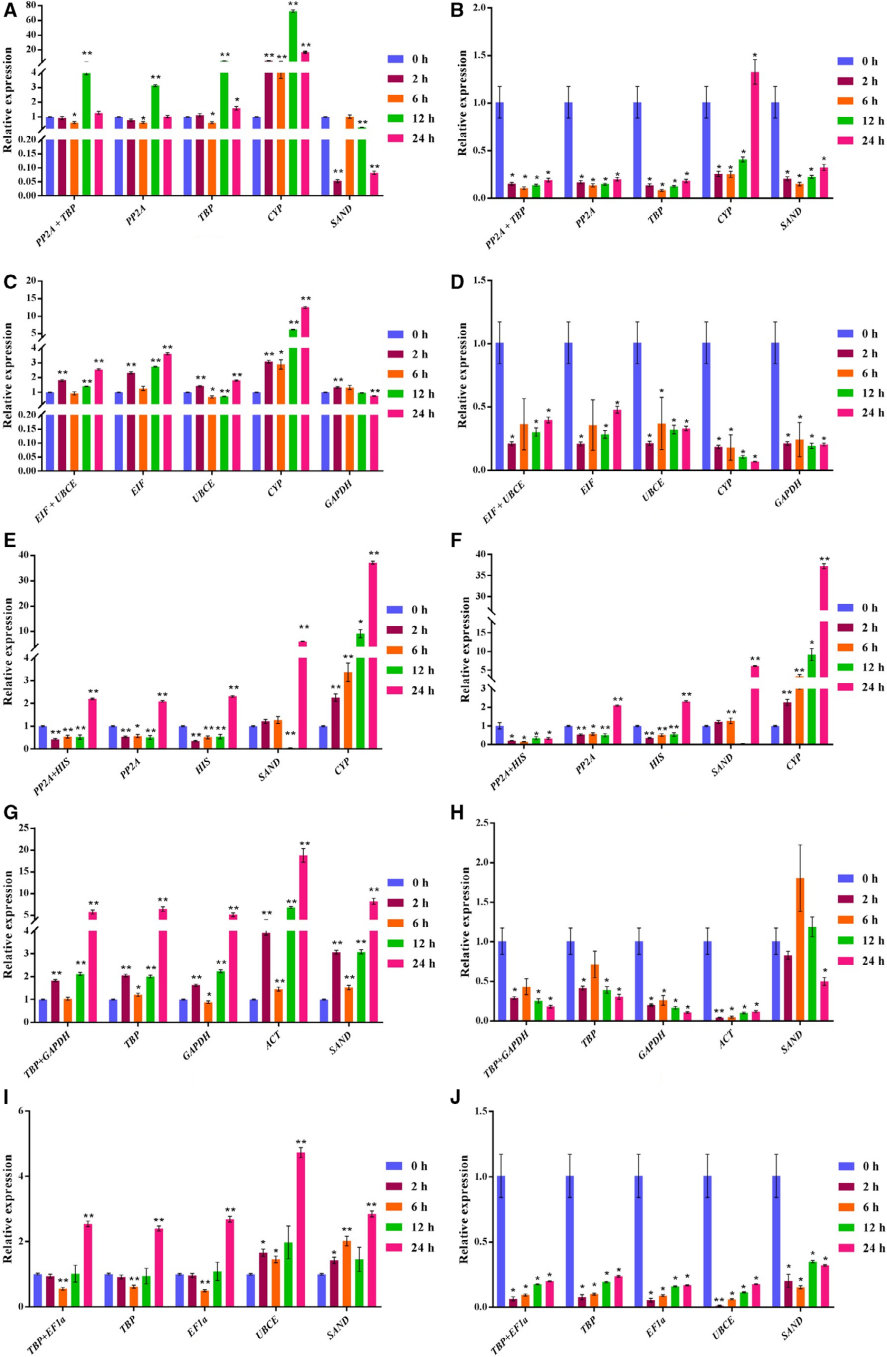
Relative quantification of *VcMATE1* expression using validated reference genes for normalization under different stress conditions. (A) Leaves treated with 110 mm NaCl. (B) Roots treated with 110 mm NaCl. (C) Leaves treated with 110 mm NaHCO_3_. (D) Roots treated with 110 mm NaHCO_3_. (E) Leaves treated with 50 mm NaCl + 70 mm NaHCO_3_. (F) Roots treated with 50 mm NaCl + 70 mm NaHCO_3_. (G) Leaves treated with 8% PEG 8000. (H) Roots treated with 8% PEG 8000. (I) Leaves treated with 100 μm AlCl_3_. (J) Roots treated with 100 μm AlCl_3_. The error bars represent the SD of three biological replicates. Asterisks indicate that the difference is significant at the level of *P < 0.05 and extremely significant at the level of **P < 0.01, *t*‐test.

Under conditions of salinity, the expression trend of *VcMATE1* in leaves calculated by stable reference genes *PP2A* + *TBP*, *PP2A* and *TBP* was similar (12 h > 24 h > 0 h ≈ 2 h > 6 h). When *CYP* or *SAND* was used as a reference gene, the expression levels of *VcMATE1* were quite different (Fig. 5A). The relative expression levels of *VcMATE1* in roots based on *PP2A* + *TBP* followed the same trend as those obtained using *TBP* or *PP2A* as the reference gene. A trend similar to the one based on these two stable reference genes (0 h > 24 h > 2 h > 12 h > 6 h) was observed when *VcMATE1* was normalized relative to *SAND*. As shown in Fig. 5B, in contrast, the trend obtained using *CYP* as the reference gene was 24 h > 0 h > 12 h > 2 h > 6 h.

When the leaves of blueberry were treated by NaHCO_3_, *VcMATE1* expressed similarly based on stable genes (*EIF* + *UBCE*, *EIF*, *UBCE*) and an unstable one (*CYP*), but the expression level of *VcMATE1* treated for 24 h was extremely high, which was different from the stable reference genes. The expression trend of *VcMATE1* based on an unstable gene, *GAPDH*, was almost flat. Therefore, *CYP* and *GAPDH* were testified not suitable as reference genes under this condition (Fig. 5C). Roots under alkaline conditions followed a decreasing trend when the least stable genes, *CYP* and *GAPDH*, were used as internal controls. In contrast, a fluctuating pattern of expression was observed for *VcMATE1* based on the most stable reference genes (*EIF* + *UBCE*, *EIF* and *UBCE*, respectively; Fig. 5D).

Under the treatment of 50 mm NaCl + 70 mm NaHCO_3_, stable candidate genes (*PP2A* + *HIS*, *PP2A* and *HIS*) were used as reference genes, and the expression of *VcMATE1* in the leaves showed a trend of decreasing first and then increasing. Taking unstable candidate genes *CYP* and *SAND* as internal controls, the expression of *VcMATE1* in leaves generally showed an upward trend. Also, the relative expression was extremely low (*SAND* as reference gene, treated for 12 h) or high (*CYP* as reference gene, treated for 24 h), which was not conducive to studying the expression pattern of the target gene (Fig. 5E). We observed that normalization of the relative expression of *VcMATE1* in blueberry roots under saline–alkaline conditions yielded similar results when the best genes (*PP2A* + *HIS*, *PP2A* and *HIS*) were used as calibrators, with major discrepancies obtained upon normalization using the worst reference genes, *CYP* and *SAND*. The relative expression level of *VcMATE1* normalized using *SAND* was nearly 0 at 12 h after stress treatment; compared with this value, 37.22‐, 2.09‐ and 2.32‐fold higher *VcMATE1* expression levels were observed at the same time point based on *CYP*, *PP2A* and *HIS*, respectively (Fig. 5F).

Under drought conditions, the relative expression of *VcMATE1* in blueberry leaves showed a similar expression trend based on the selected stable (*TBP* + *GAPDH*, *TBP* and *GAPDH*) and unstable (*ACT* and *SAND*) reference genes. However, treated for 24 h, the expression level of the target gene with *ACT* as reference gene significantly increased, which would lead to the unreliable expression pattern (Fig. 5G). *VcMATE1* expression levels in roots under simulated drought conditions decreased regularly (0 h > 6 h > 2 h > 12 h > 24 h) based on *TBP* + *GAPDH*, *TBP* and *GAPDH* as internal reference genes. Obviously different expression trends were obtained when the least stable reference genes were used as internal controls (*SAND*: 6 h > 12 h > 0 h > 6 h > 24 h; *ACT*: 0 h > 24 h > 12 h > 6 h > 2 h; Fig. 5H).

Expression levels of *VcMATE1* in leaves under AlCl_3_ treatment conditions followed the trend of 24 h > 12 h > 0 h > 2 h > 6 h when *TBP* + *EF1α*, *TBP* and *EF1α* were used as internal controls. In contrast, the patterns of *VcMATE1* expression normalized according to the least stable genes, *UBCE* and *SAND*, were 24 h > 12 h > 2 h > 6 h > 0 h and 24 h > 6 h > 12 h > 2 h > 0 h, respectively (Fig. 5I). In roots, when the most stable genes (*TBP* + *EF1α*, *TBP* and *EF1α*) were selected as reference genes, the relative expression trends of *VcMATE1* were 0 h > 24 h > 12 h > 6 h > 2 h. When the unstable gene, *UBCE*, was used as reference gene, the expression trend of the target gene decreased first and then increased, but after 2 h of treatment, the relative expression was almost zero. A different expression trend was observed when the least stable reference gene was used as internal control (*SAND*: 0 h > 12 h > 24 h > 2 h > 6 h; Fig. 5J). All of this indicated that unstable candidate genes were not reliable as reference genes.

These results confirm the feasibility and reliability of the selected reference genes.

## Discussion

With the development of biotechnology, RT‐qPCR has been applied widely for analysis of gene expression, and the selection of appropriate internal reference genes is recognized as the primary prerequisite for reliable and accurate real‐time results. Nevertheless, previous studies on reference gene standardization have demonstrated that reference gene stability is not absolutely constant in diverse species and organs, and under different abiotic/biotic conditions and developmental stages. Even reference genes that work well in model plants may barely be applicable to other species. For instance, *ACT* is invariably considered to be the best choice in a variety of model species, including *Arabidopsis thaliana* under abiotic stresses (salt, drought and cold) [33, 34, 35, 36, 37], *Nicotiana tabacum* under stress treatment (heat, cold, drought, salt and UV) [38] and *Oryza sativa* subjected to NaCl and abscisic acid (ABA) treatments [39]. Moreover, the same reference gene is often not applicable across closely related species. *ACT* has been found to be the most stable reference gene for *Vitis vinifera* under salt and osmotic stresses [40, 41]. In blueberry under salt stress, *ACT* ranked eighth out of 14 candidate genes in our study, thus demonstrating that it was not stably expressed under our experimental conditions. *UBC*, another reference gene used in the genus *Vaccinium*, has been found to be suitable as an internal control in different organs of both rabbiteye and southern highbush blueberry [42], but did not perform well in the half‐high blueberry cultivar ‘Northland’ in our study.

Several factors may be responsible for the earlier‐mentioned variation and observed differences in the expression stability of candidate reference genes. First, RNA expression levels are not constant under all conditions, with those of internal reference genes varying because of differences in factors such as cell‐cycle stage, species, materials and sequencing libraries. Second, we used three main algorithms, NormFinder, geNorm and BestKeeper, to analyze the data obtained in this study [17, 43, 44]. These three methods are the ones currently used by researchers to assess the stability of candidate genes for use as reference genes in RT‐qPCR analyses. NormFinder can generate the best reference gene or best combination, whereas geNorm can select a combination of reference genes and rank them by suitability. Unlike NormFinder and geNorm, BestKeeper does not require preprocessing of data and can directly make use of C*_t_* values obtained by RT‐qPCR for calculations [44]. It is not an exaggeration to say that a comprehensive analysis using multiple methods is the best way to obtain the optimal reference gene.

To date, no reference gene has been found to be suitable in all types of cells or organs [17, 43, 45]. Researchers should therefore conduct preliminary experiments to identify stably expressed reference genes based on the type of cells and organs to be studied and their experimental requirements. At the same time, the expressions of two or more internal reference genes, chosen using an algorithm that selects multiple reference genes, can be averaged and used to normalize the specific target gene expression data to obtain more reliable results.

In this study, we evaluated genes that have been frequently used as internal controls in a large number of species. The most stably expressed genes in various organs and the five abiotic stress conditions were *EIF*/*EF1α* and *EIF*/*TBP*, respectively. In a previous investigation, *EF1α* was found to be the most suitable reference gene in *O*.* sativa* [46] and *Solanum tuberosum* [44] during different development stages and under hormone, salt and drought treatments. *EF1α* and *EIF4A* were determined to be the most stable genes for use in different organ and abiotic stress subsets (ABA, drought, salt and high/low temperature) in *Pennisetum glaucum* [47]. Some, although not all, findings in other species are consistent with those of our study, thus indicating that our results are also credible. Regardless of whether our results are consistent with the conclusions of other studies, however, our observations demonstrate that the reference gene most suitable for a set of experimental conditions and a specific analysis should be selected and further evaluated prior to measurements of gene expression levels.

Finally, to further confirm the accuracy of the results of this study, we selected the *VcMATE1* gene, a member of the MATE (multidrug and toxic compound extrusion transporter) family, which phylogenetic analysis has clustered with genes involved in the detoxification of xenobiotics or export of toxic cations [32 ]. On the basis of its predicted function, we expected *VcMATE1* to respond to diverse abiotic stress conditions and thus be of interest in future studies of blueberry stress resistance. We therefore normalized expression levels of *VcMATE1* using the two most stable and two least stable reference genes in each treatment subgroup. Relative expression levels of the *VcMATE1* gene normalized using the most stable reference gene were the most consistent. Moreover, the selected genes were stable under normal conditions over time, which indicates that our study results are significant and valuable.

## Conclusions

To ensure the accuracy of gene expression analyses, we selected 14 candidate reference genes from a blueberry fruit transcriptome and analyzed them by RT‐qPCR to identify the most appropriate ones for the normalization of potential functional gene expression data. In this study, we determined the optimal set of reference genes for different organs of blueberry under normal and abiotic stress conditions. In all organs under nonstress conditions, *EIF* + *EF1α* was the best choice, whereas *EIF* + *TBP* was the best combination under all five abiotic stresses. We provided more specific reference gene recommendations for analyses of expression under individual stresses: *PP2A*/*TBP* (salinity), *EIF*/*UBCE* (alkalinity), *PP2A*/*HIS* (salinity–alkalinity) and *TBP/GAPDH* (drought) and* TBP/EF1α* (AlCl_3_). The use of these reference genes should aid future studies of molecular mechanisms of stress resistance and molecular breeding in blueberry.

## Conflict of interest

The authors declare no conflict of interest.

## Author contributions

HS conceived and designed the project. YD performed the experiments, analyzed the data and wrote the manuscript. YL provided the blueberry samples. HS and YL contributed to the manuscript writing review and editing.

## Supporting information


**Fig**.** S1**. Abiotic treatments of 2‐year‐old cutting plants of blueberry. (A) 110 mm NaCl treatment. (B) 110 mm NaHCO_3_ treatment. (C) 50 mm NaCl + 70 mm NaHCO_3_ treatment. (D) 8% PEG8000 treatment. (E) 100 μm AlCl_3_ treatment.

**Fig**.** S2**. Agarose gel electrophoresis for total RNA of blueberry.

**Fig**.** S3**. Products of RT‐qPCR of 14 candidate reference genes.

**Table S1**. Description of the samples under abiotic stresses used for RT‐qPCR.

**Table S2**. Selection of candidate reference genes based on blueberry fruit transcriptome.

**Table S3**. RNA quantification of blueberry under different experimental conditions. (A) RNA quantification of different tissues of blueberry under common condition. (B) RNA quantification of tissues of blueberry in different abiotic conditions.

**Table S4**. The amplification specificity of 14 candidate reference mRNA genes.

**Table S5**. (A) Expression stability ranking of 14 candidate reference genes in leaves of blueberry under abiotic stresses by NormFinder. (B) Expression stability ranking of 14 candidate reference genes in leaves of blueberry under abiotic stresses by NormFinder.

**Table S6**. Expression stability ranking of 14 candidate reference genes in leaves and roots of blueberry under abiotic stresses by BestKeeper.

**Table S7**. Expression stability comprehensive ranking of 14 candidate reference genes in leaves and roots under abiotic stresses. (A) Expression stability comprehensive ranking of 14 candidate reference genes in leaves under abiotic stresses. (B) Expression stability comprehensive ranking of 14 candidate reference genes in roots under abiotic stresses.

**Data S1**. Primer pair annealing locations on their respective transcripts.

**Data S2**. The C*_t_* values of 14 candidate genes in different tissues of blueberry.

**Data S3**. The C*_t_* values of 14 candidate genes in leafs and roots of blueberry under different abiotic stresses.

**Data S4**. (A) The C*_t_* values of *VcMATE1* in different tissues of blueberry (three replicates). (B) The C*_t_* values of *VcMATE1* of blueberry under different abiotic stresses (three replicates).

## Data Availability

Model data are available in the European Nucleotide Archive under accession number KF875433.
