# Supplementary figures and images for: Selection of reference genes for RT‐qPCR normalization in blueberry (Vaccinium corymbosum × angustifolium) under various abiotic stresses
Source: FEBS Open Bio. 2020 Jun 23;10(8):1418–35. doi: 10.1002/2211-5463.12903 (PMC7396441; doi:10.1002/2211-5463.12903)

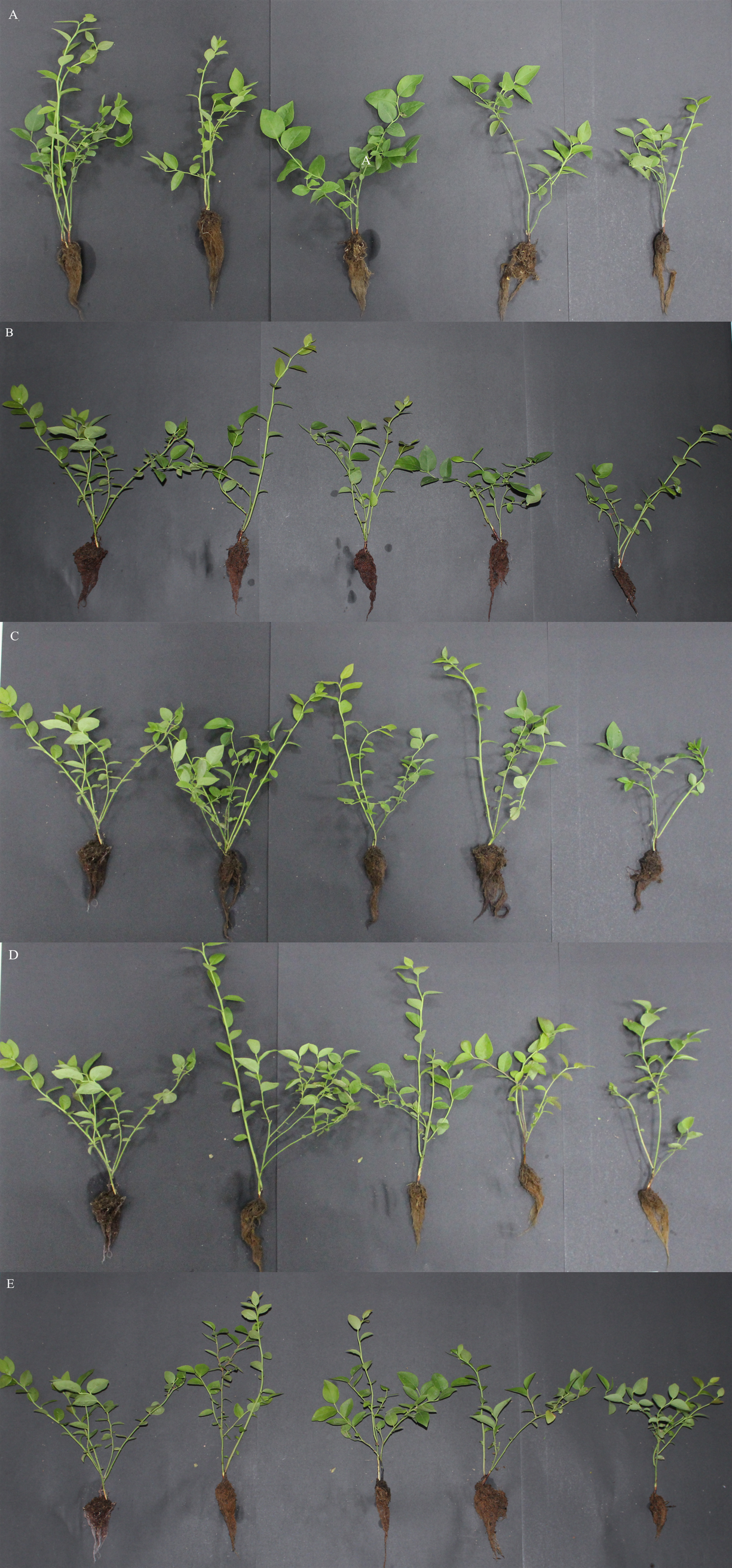

Supplement: Supplementary file 1 — Fig. S1. Abiotic treatments of 2‐year‐old cutting plants of blueberry. (A) 110 mm NaCl treatment. (B) 110 mm NaHCO3 treatment. (C) 50 mm NaCl + 70 mm NaHCO3 treatment. (D) 8% PEG8000 treatment. (E) 100 μm AlCl3 treatment. [file FEB4-10-1418-s001.tif]

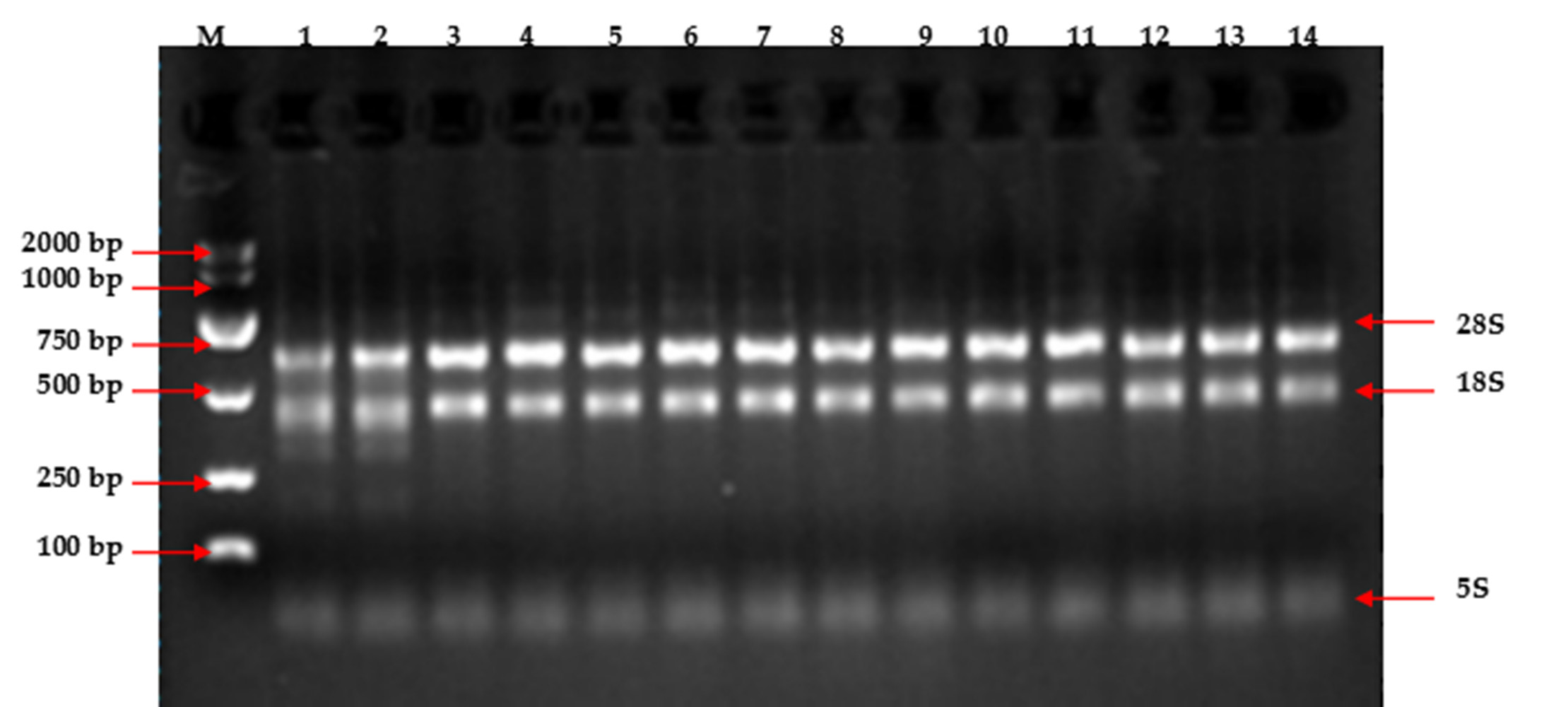

Supplement: Supplementary file 2 — Fig. S2. Agarose gel electrophoresis for total RNA of blueberry. [file FEB4-10-1418-s002.tif]

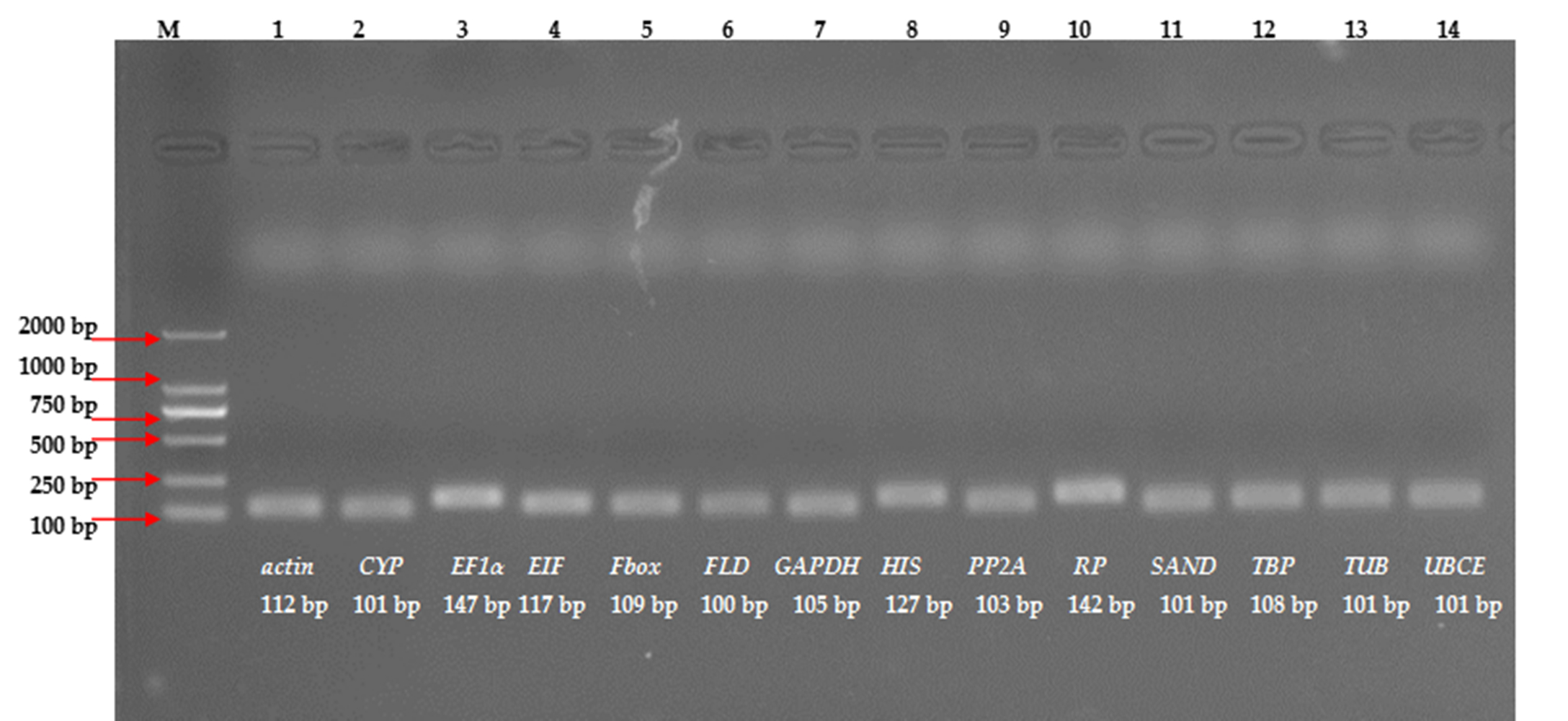

Supplement: Supplementary file 3 — Fig. S3. Products of RT‐qPCR of 14 candidate reference genes. [file FEB4-10-1418-s003.tif]
