# Supplementary material for: Selection of reference genes for RT‐qPCR normalization in blueberry (Vaccinium corymbosum × angustifolium) under various abiotic stresses
Source: FEBS Open Bio. 2020 Jun 23;10(8):1418–35. doi: 10.1002/2211-5463.12903 (PMC7396441; doi:10.1002/2211-5463.12903)
Supplement: Supplementary file 4 — Table S1. Description of the samples under abiotic stresses used for RT‐qPCR. [file FEB4-10-1418-s004.doc]

**Table S1. Description of the samples under abiotic stresses used for RT-qPCR.**

| **Sample No.** | **Cultivar** | **Developmental stage** | **Abiotic Stress Conditions** | **Treated time** | **Tissue** | **The number of technical replicates** | **The number of biological replicates** |
| --- | --- | --- | --- | --- | --- | --- | --- |
| **1** | Northland | Two years old cutting plants | Salinity (110 mM NaCl) | 2 hours | Leaves | 3 | 3 |
| **2** | Northland | Two years old cutting plants | Salinity (110 mM NaCl) | 6 hours | Leaves | 3 | 3 |
| **3** | Northland | Two years old cutting plants | Salinity (110 mM NaCl) | 12 hours | Leaves | 3 | 3 |
| **4** | Northland | Two years old cutting plants | Salinity (110 mM NaCl) | 24 hours | Leaves | 3 | 3 |
| **5** | Northland | Two years old cutting plants | Salinity (110 mM NaCl) | 2 hours | Roots | 3 | 3 |
| **6** | Northland | Two years old cutting plants | Salinity (110 mM NaCl) | 6 hours | Roots | 3 | 3 |
| **7** | Northland | Two years old cutting plants | Salinity (110 mM NaCl) | 12 hours | Roots | 3 | 3 |
| **8** | Northland | Two years old cutting plants | Salinity (110 mM NaCl) | 24 hours | Roots | 3 | 3 |
| **9** | Northland | Two years old cutting plants | Aalkalinity (110 mM NaHCO_3_) | 2 hours | Leaves | 3 | 3 |
| **10** | Northland | Two years old cutting plants | Aalkalinity (110 mM NaHCO_3_) | 6 hours | Leaves | 3 | 3 |
| **11** | Northland | Two years old cutting plants | Aalkalinity (110 mM NaHCO_3_) | 12 hours | Leaves | 3 | 3 |
| **12** | Northland | Two years old cutting plants | Aalkalinity (110 mM NaHCO_3_) | 24 hours | Leaves | 3 | 3 |
| **13** | Northland | Two years old cutting plants | Aalkalinity (110 mM NaHCO_3_) | 2 hours | Roots | 3 | 3 |
| **14** | Northland | Two years old cutting plants | Aalkalinity (110 mM NaHCO_3_) | 6 hours | Roots | 3 | 3 |
| **15** | Northland | Two years old cutting plants | Aalkalinity (110 mM NaHCO_3_) | 12 hours | Roots | 3 | 3 |
| **16** | Northland | Two years old cutting plants | Aalkalinity (110 mM NaHCO_3_) | 24 hours | Roots | 3 | 3 |
| **17** | Northland | Two years old cutting plants | Salinity and alkalinity  (50 mM NaCl and 70 mM NaHCO_3_) | 2 hours | Leaves | 3 | 3 |
| **18** | Northland | Two years old cutting plants | Salinity and alkalinity  (50 mM NaCl and 70 mM NaHCO_3_) | 6 hours | Leaves | 3 | 3 |
| **19** | Northland | Two years old cutting plants | Salinity and alkalinity  (50 mM NaCl and 70 mM NaHCO_3_) | 12 hours | Leaves | 3 | 3 |
| **20** | Northland | Two years old cutting plants | Salinity and alkalinity  (50 mM NaCl and 70 mM NaHCO_3_) | 24 hours | Leaves | 3 | 3 |
| **21** | Northland | Two years old cutting plants | Salinity and alkalinity  (50 mM NaCl and 70 mM NaHCO_3_) | 2 hours | Roots | 3 | 3 |
| **22** | Northland | Two years old cutting plants | Salinity and alkalinity  (50 mM NaCl and 70 mM NaHCO_3_) | 6 hours | Roots | 3 | 3 |
| **23** | Northland | Two years old cutting plants | Salinity and alkalinity  (50 mM NaCl and 70 mM NaHCO_3_) | 12 hours | Roots | 3 | 3 |
| **24** | Northland | Two years old cutting plants | Salinity and alkalinity  (50 mM NaCl and 70 mM NaHCO_3_) | 24 hours | Roots | 3 | 3 |
| **25** | Northland | Two years old cutting plants | Drought (8% PEG8000) | 2 hours | Leaves | 3 | 3 |
| **26** | Northland | Two years old cutting plants | Drought (8% PEG8000) | 6 hours | Leaves | 3 | 3 |
| **27** | Northland | Two years old cutting plants | Drought (8% PEG8000) | 12 hours | Leaves | 3 | 3 |
| **28** | Northland | Two years old cutting plants | Drought (8% PEG8000) | 24 hours | Leaves | 3 | 3 |
| **29** | Northland | Two years old cutting plants | Drought (8% PEG8000) | 2 hours | Roots | 3 | 3 |
| **30** | Northland | Two years old cutting plants | Drought (8% PEG8000) | 6 hours | Roots | 3 | 3 |
| **31** | Northland | Two years old cutting plants | Drought (8% PEG8000) | 12 hours | Roots | 3 | 3 |
| **32** | Northland | Two years old cutting plants | Drought (8% PEG8000) | 24 hours | Roots | 3 | 3 |
| **33** | Northland | Two years old cutting plants | AlCl_3_ (100uM AlCl_3_ ) | 2 hours | Leaves | 3 | 3 |
| **34** | Northland | Two years old cutting plants | AlCl_3_ (100uM AlCl_3_ ) | 6 hours | Leaves | 3 | 3 |
| **35** | Northland | Two years old cutting plants | AlCl_3_ (100uM AlCl_3_ ) | 12 hours | Leaves | 3 | 3 |
| **36** | Northland | Two years old cutting plants | AlCl_3_ (100uM AlCl_3_ ) | 24 hours | Leaves | 3 | 3 |
| **37** | Northland | Two years old cutting plants | AlCl_3_ (100uM AlCl_3_ ) | 2 hours | Roots | 3 | 3 |
| **38** | Northland | Two years old cutting plants | AlCl_3_ (100uM AlCl_3_ ) | 6 hours | Roots | 3 | 3 |
| **39** | Northland | Two years old cutting plants | AlCl_3_ (100uM AlCl_3_ ) | 12 hours | Roots | 3 | 3 |
| **40** | Northland | Two years old cutting plants | AlCl_3_ (100uM AlCl_3_ ) | 12 hours | Roots | 3 | 3 |
| **41** | Northland | Two years old cutting plants | Blank control (No treatment) | 0 hours | Leaves | 3 | 3 |
| **42** | Northland | Two years old cutting plants | Blank control (No treatment) | 0 hours | Roots | 3 | 3 |

^1^ Three biological replicates have been pooled together after cDNA synthesis for expression stability assessment of candidate reference mRNA/miRNA.

^2^ The sets of samples. (1) Leaf and root combined samples (n = 42). (2) Leaf samples (n = 21). (3) Root samples (n = 21).
