# Supplementary material for: Selection of reference genes for RT‐qPCR normalization in blueberry (Vaccinium corymbosum × angustifolium) under various abiotic stresses
Source: FEBS Open Bio. 2020 Jun 23;10(8):1418–35. doi: 10.1002/2211-5463.12903 (PMC7396441; doi:10.1002/2211-5463.12903)
Supplement: Supplementary file 5 — Table S2. Selection of candidate reference genes based on blueberry fruit transcriptome. [file FEB4-10-1418-s005.doc]

**Table S2. Selection of candidate reference genes based on blueberry fruit transcriptome.**

| **Gene Symbol** | **Gene ID** | **TARAAPE_RPKM** | **TBRAAPE_RPKM** | **log_2_(TBRAAPE_RPKM/TARAAPE_RPKM)** |
| --- | --- | --- | --- | --- |
| ***Actin*** | Unigene18644_All | 708.311 | 214.6277 | -1.722546664 |
|  | Unigene2464_All | 59.346 | 41.9122 | -0.501780542 |
|  | Unigene7520_All | 145.0453 | 95.1082 | -0.60886191 |
| ***CYP*** | Unigene13197_All | 40.5787 | 40.4744 | -0.003712953 |
|  | Unigene5225_All | 240.385 | 448.9981 | 0.901362465 |
| ***EF1α*** | Unigene12271_All | 685.0639 | 830.3016 | 0.277396915 |
|  | Unigene16052_All | 524.7603 | 995.125 | 0.923219178 |
|  | Unigene20628_All | 401.6106 | 833.4053 | 1.053220927 |
|  | Unigene31695_All | 0.616 | 5.5199 | 3.163639875 |
| ***EIF*** | Unigene19256_All | 250.9837 | 239.2325 | -0.069180278 |
| ***Fbox*** | Unigene29248_All | 1.8395 | 3.663 | 0.993712025 |
|  | Unigene7226_All | 9.1991 | 8.8816 | -0.050673123 |
| ***FLD*** | Unigene28351_All | 3.4496 | 3.3721 | -0.032781763 |
|  | Unigene31589_All | 2.176 | 4.5583 | 1.066817321 |
| ***GAPDH*** | Unigene12031_All | 126.8869 | 220.6496 | 0.798214001 |
|  | Unigene1467_All | 28.4727 | 15.0435 | -0.920439045 |
|  | Unigene14856_All | 99.3536 | 289.8544 | 1.544684237 |
|  | Unigene17625_All | 596.6472 | 656.4584 | 0.137825478 |
|  | Unigene30876_All | 4.286 | 16.8393 | 1.974128413 |
|  | Unigene4159_All | 1375.6673 | 230.4881 | -2.57736743 |
| ***HIS*** | Unigene8208_All | 153.1042 | 143.9564 | -0.08888193 |
| ***PP2A*** | Unigene14576_All | 30.1756 | 31.5648 | 0.064934153 |
|  | Unigene9933_All | 10.3966 | 3.6297 | -1.518189584 |
| ***RP*** | Unigene1133_All | 9.824 | 15.0874 | 0.618961743 |
|  | Unigene15023_All | 75.1492 | 51.1261 | -0.555697767 |
|  | Unigene17877_All | 11.0336 | 7.0253 | -0.651271846 |
|  | Unigene33482_All | 1.444 | 3.3609 | 1.218776875 |
|  | Unigene5012_All | 3.45 | 6.8333 | 0.985986104 |
| ***SAND*** | Unigene12206_All | 29.2467 | 25.7054 | -0.186202388 |
| ***TBP*** | Unigene11381_All | 37.4796 | 41.8497 | 0.159111725 |
| ***TUB*** | Unigene30545_All | 4.7274 | 1.7263 | -1.45336374 |
|  | Unigene3235_All | 18.851 | 6.8729 | -1.455650183 |
|  | Unigene32370_All | 402.9463 | 169.8626 | -1.246219348 |
|  | Unigene4780_All | 15.5758 | 15.5318 | -0.004081229 |
|  | Unigene8911_All | 60.6274 | 32.3283 | -0.907172309 |
| ***UBCE*** | Unigene10606_All | 175.1778 | 96.7266 | -0.856835363 |
|  | Unigene1297_All | 54.2946 | 93.8683 | 0.789829313 |
|  | Unigene18373_All | 180.3585 | 75.917 | -1.248372529 |
|  | Unigene18931_All | 12.3909 | 3.8235 | -1.696315201 |
|  | Unigene4251_All | 236.2412 | 245.37 | 0.05469828 |
|  | Unigene5826_All | 208.0161 | 132.6144 | -0.649457754 |
