# Supplementary material for: Selection of reference genes for RT‐qPCR normalization in blueberry (Vaccinium corymbosum × angustifolium) under various abiotic stresses
Source: FEBS Open Bio. 2020 Jun 23;10(8):1418–35. doi: 10.1002/2211-5463.12903 (PMC7396441; doi:10.1002/2211-5463.12903)
Supplement: Supplementary file 6 — Table S3. RNA quantification of blueberry under different experimental conditions. (A) RNA quantification of different tissues of blueberry under common condition. (B) RNA quantification of tissues of blueberry in different abiotic conditions. [file FEB4-10-1418-s006.doc]

**Table S3 (A) RNA quantification of different tissues of blueberry under common condition.**

| **Tissues** | **Concentration (ng/μL)** | **260/280** | **260/230** |
| --- | --- | --- | --- |
| **Root** | 1435 | 2.014 | 1.936 |
| **Stem** | 1394 | 2.068 | 2.070 |
| **Leaf** | 2032 | 2.004 | 2.056 |
| **Flower** | 2145 | 1.837 | 2.091 |
| **Leaf bud** | 2034 | 1.938 | 1.974 |
| **Flower bud** | 1937 | 2.043 | 1.923 |
| **Green fruit** | 1038 | 2.097 | 2.037 |
| **Pink fruit** | 998 | 2.037 | 1.849 |
| **Blue fruit** | 1136 | 2.083 | 2.003 |
| **Seed** | 835 | 1.962 | 2.049 |
| **Exocarp** | 1039 | 2.035 | 1.959 |
| **Sarcocarp** | 844 | 2.099 | 2.011 |

**Table S3 (B) RNA quantification of tissues of blueberry in different abiotic conditions.**

| **Abiotic conditions** | **Treated time (hour)** | **Tissues** | | | | | |
| --- | --- | --- | --- | --- | --- | --- | --- |
|  |  | **Leaves** | | | **Roots** | | |
|  |  | **Concentration (ng/μL)** | **260/280** | **260/230** | **Concentration (ng/μL)** | **260/280** | **260/230** |
| **Salinity (110 mM NaCl)** | 2 | 1816 | 1.985 | 2.030 | 1630 | 2.028 | 1.869 |
|  | 6 | 1322 | 2.001 | 2.019 | 1758 | 2.007 | 2.013 |
|  | 12 | 305 | 2.028 | 1.998 | 1620 | 1.966 | 2.016 |
|  | 24 | 668 | 2.067 | 2.073 | 704 | 1.989 | 2.086 |
| **Aalkalinity (100 mM NaHCO_3_)** | 2 | 1530 | 2.103 | 1.890 | 1606 | 2.070 | 2.048 |
|  | 6 | 1086 | 2.013 | 2.073 | 900 | 2.027 | 2.056 |
|  | 12 | 1400 | 2.098 | 2.083 | 1298 | 2.094 | 2.018 |
|  | 24 | 1670 | 2.024 | 1.902 | 280 | 2.053 | 2.372 |
| **Salinity and alkalinity (50 mM NaCl and 70 mM NaHCO_3_)** | 2 | 1928 | 1.892 | 1.895 | 658 | 2.082 | 2.084 |
|  | 6 | 1614 | 2.018 | 2.048 | 942 | 2.030 | 2.091 |
|  | 12 | 1484 | 2.035 | 1.923 | 910 | 2.068 | 2.107 |
|  | 24 | 2034 | 1.937 | 2.034 | 578 | 2.035 | 2.050 |
| **Drought (8% PEG8000)** | 2 | 1462 | 2.007 | 2.019 | 1274 | 2.048 | 2.059 |
|  | 6 | 1822 | 2.038 | 1.937 | 1340 | 1.968 | 2.039 |
|  | 12 | 1930 | 1.963 | 2.074 | 1370 | 2.057 | 2.168 |
|  | 24 | 1846 | 1.829 | 2.023 | 988 | 1.992 | 1.878 |
| **AlCl_3_ (100uM AlCl_3_ )** | 2 | 1816 | 2.003 | 2.048 | 1242 | 2.003 | 1.959 |
|  | 6 | 818 | 2.084 | 2.017 | 1390 | 2.014 | 2.079 |
|  | 12 | 1724 | 1.803 | 2.134 | 1230 | 2.030 | 2.075 |
|  | 24 | 1646 | 2.090 | 1.912 | 1158 | 2.046 | 2.036 |
| **Control (No treatment)** | 0 | 1476 | 2.034 | 2.042 | 1037 | 2.087 | 1.928 |
