# Supplementary material for: Selection of reference genes for RT‐qPCR normalization in blueberry (Vaccinium corymbosum × angustifolium) under various abiotic stresses
Source: FEBS Open Bio. 2020 Jun 23;10(8):1418–35. doi: 10.1002/2211-5463.12903 (PMC7396441; doi:10.1002/2211-5463.12903)
Supplement: Supplementary file 7 — Table S4. The amplification specificity of 14 candidate reference mRNA genes. [file FEB4-10-1418-s007.doc]

**Table S4.** **The amplification specificity of 14 candidate reference mRNA genes.**

| **Symbol** | **Amplification plots** | **Dissociation curve** | **Standard curves** |
| --- | --- | --- | --- |
| ***actin*** | **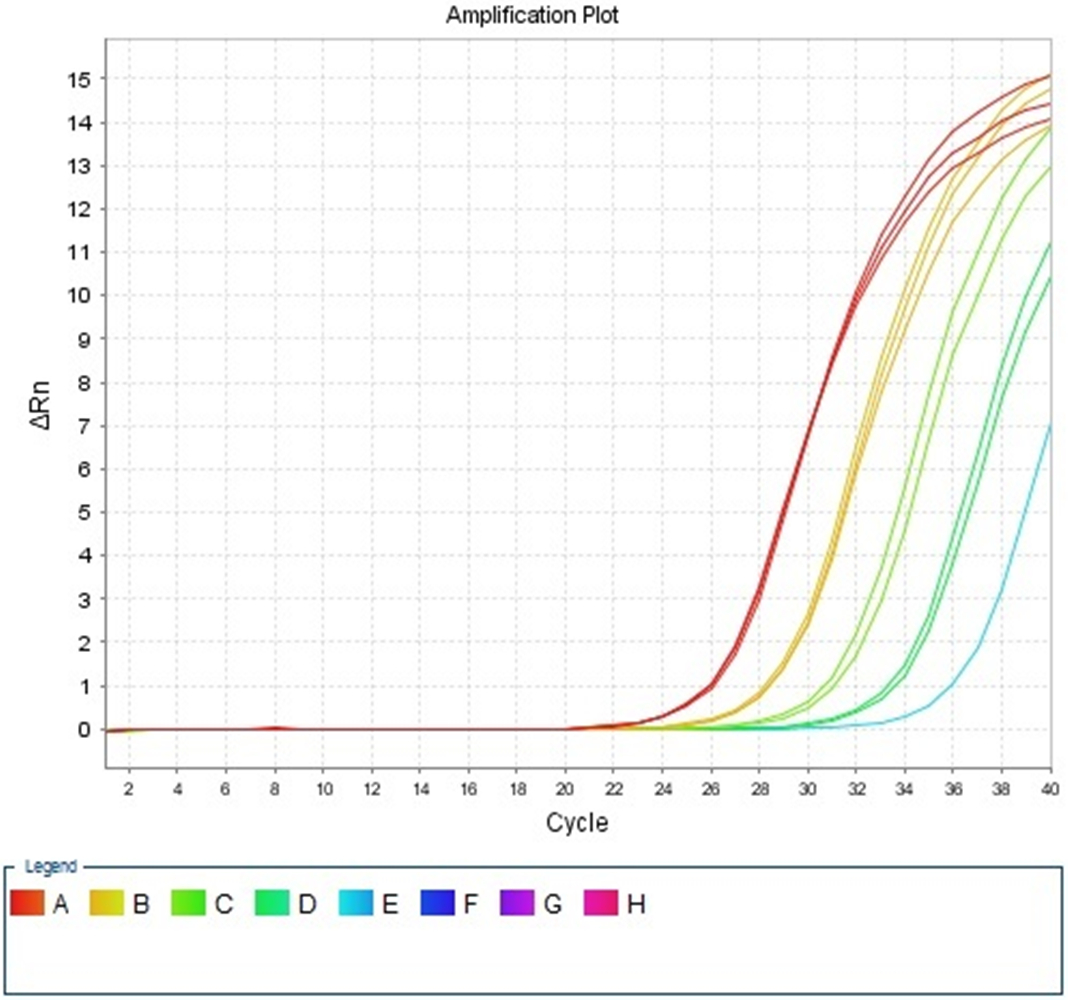** | **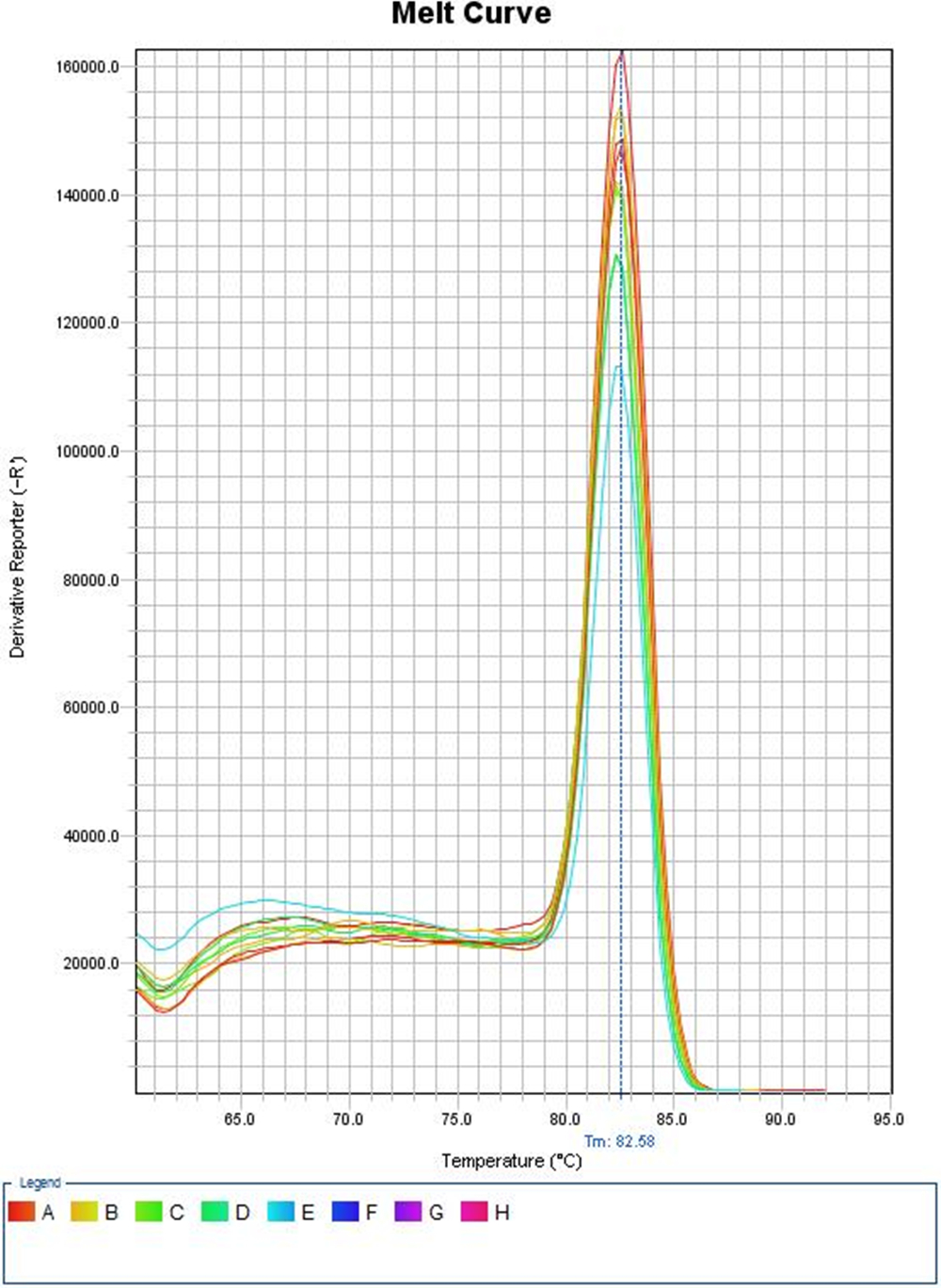** | **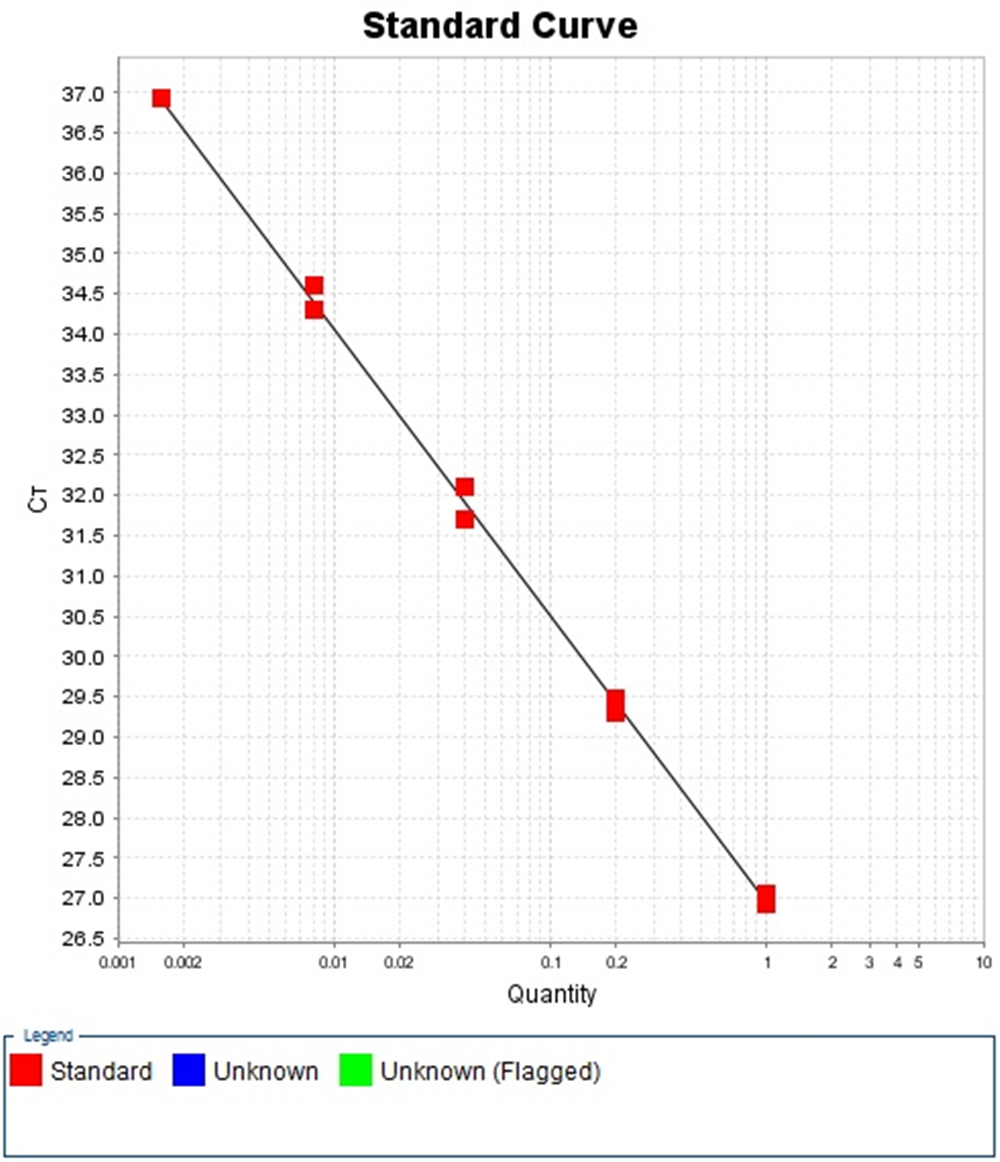** |
| ***CYP*** | **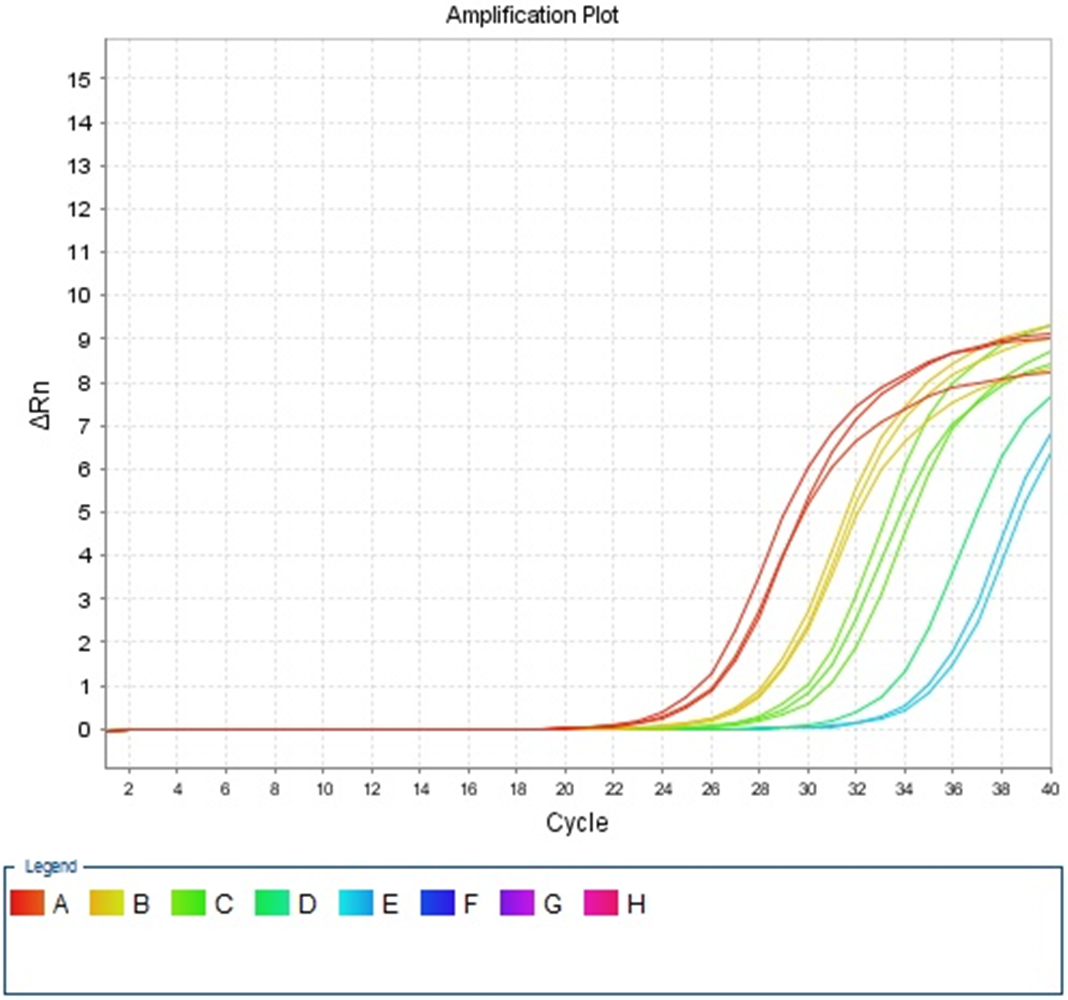** | **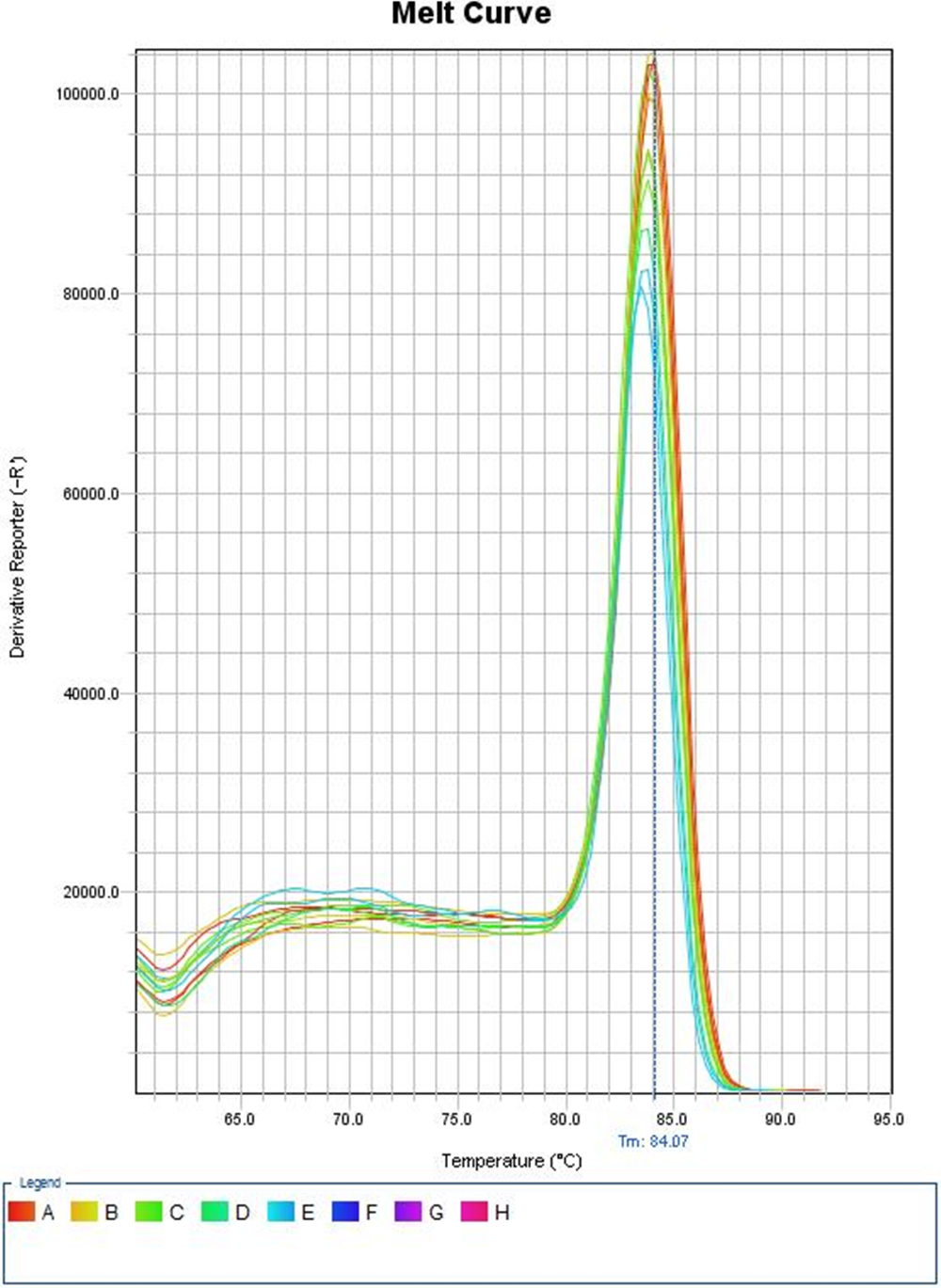** | **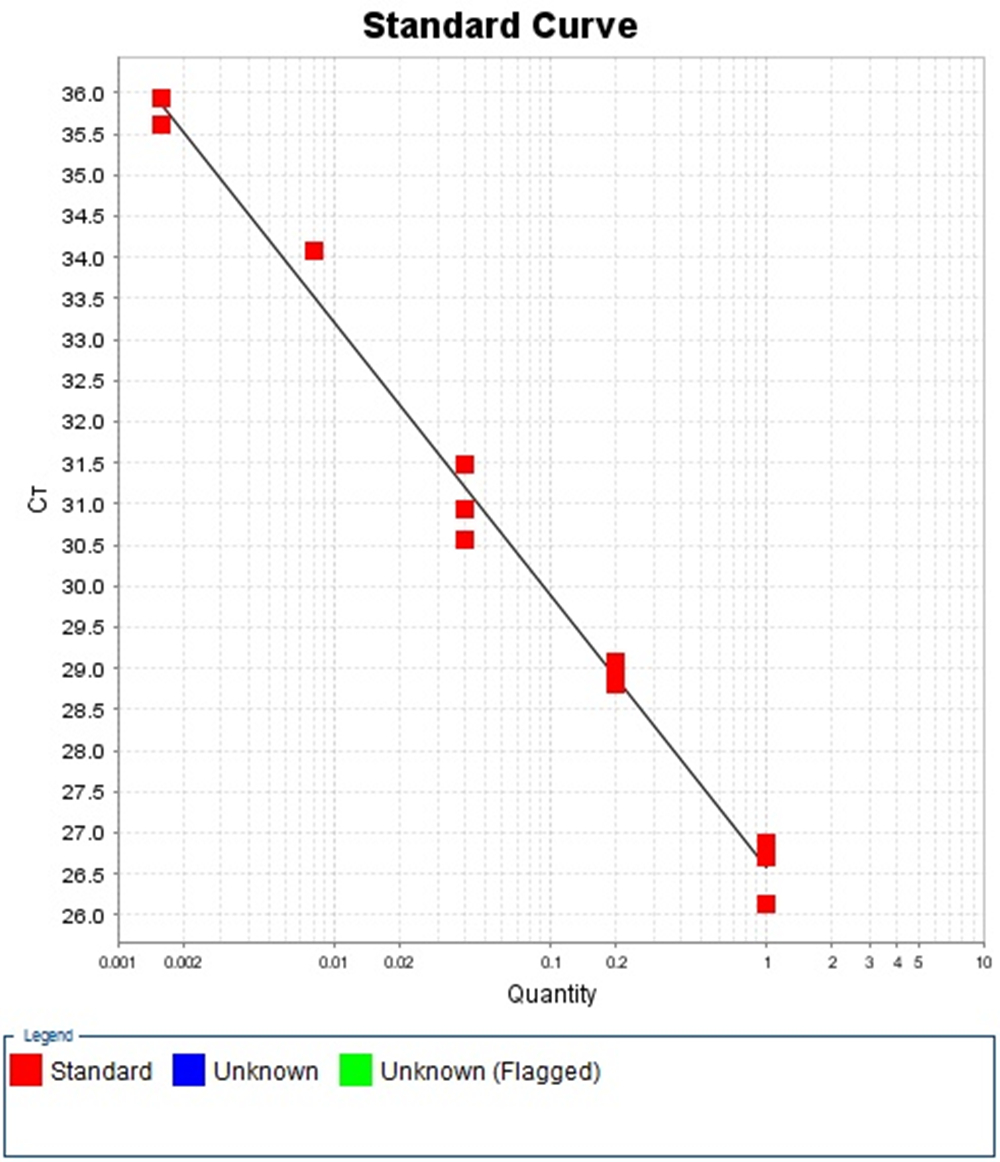** |
| ***EF1a*** | **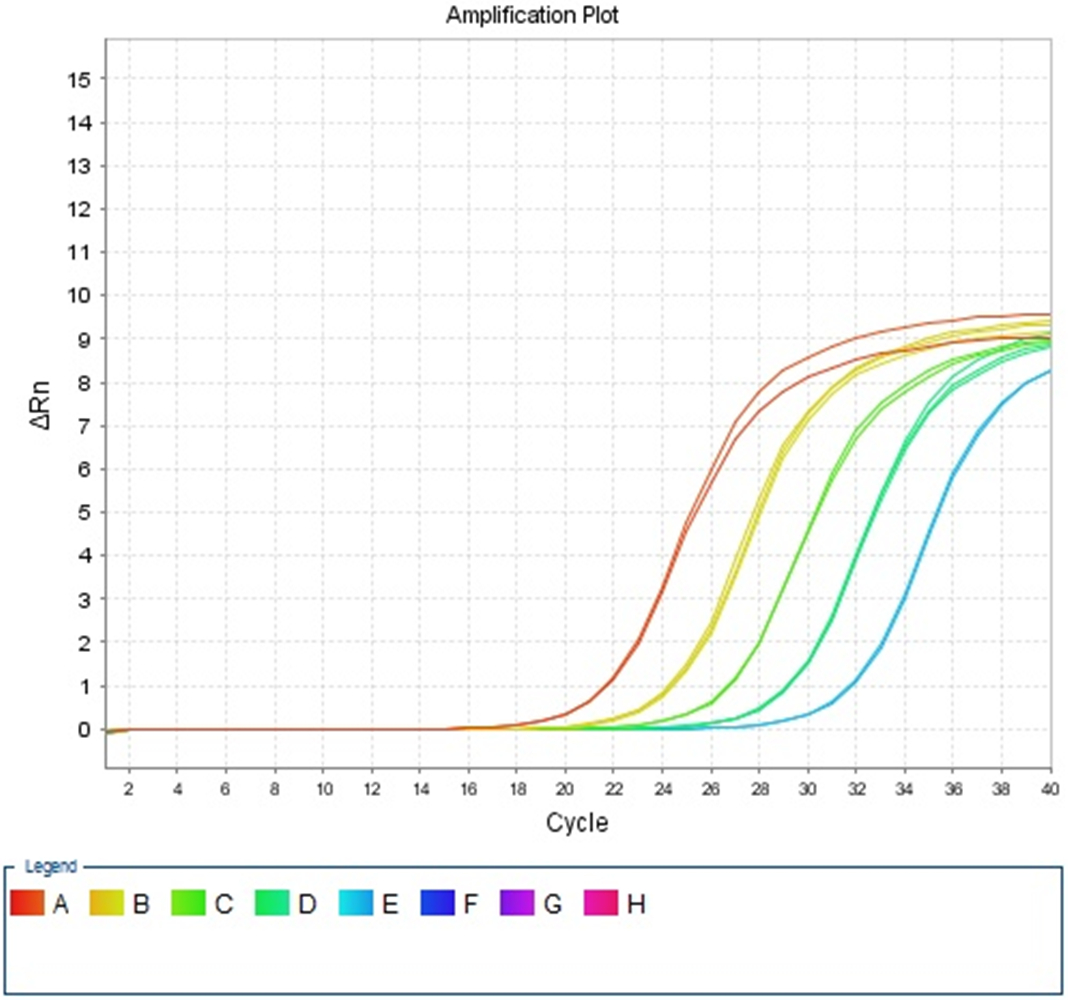** | **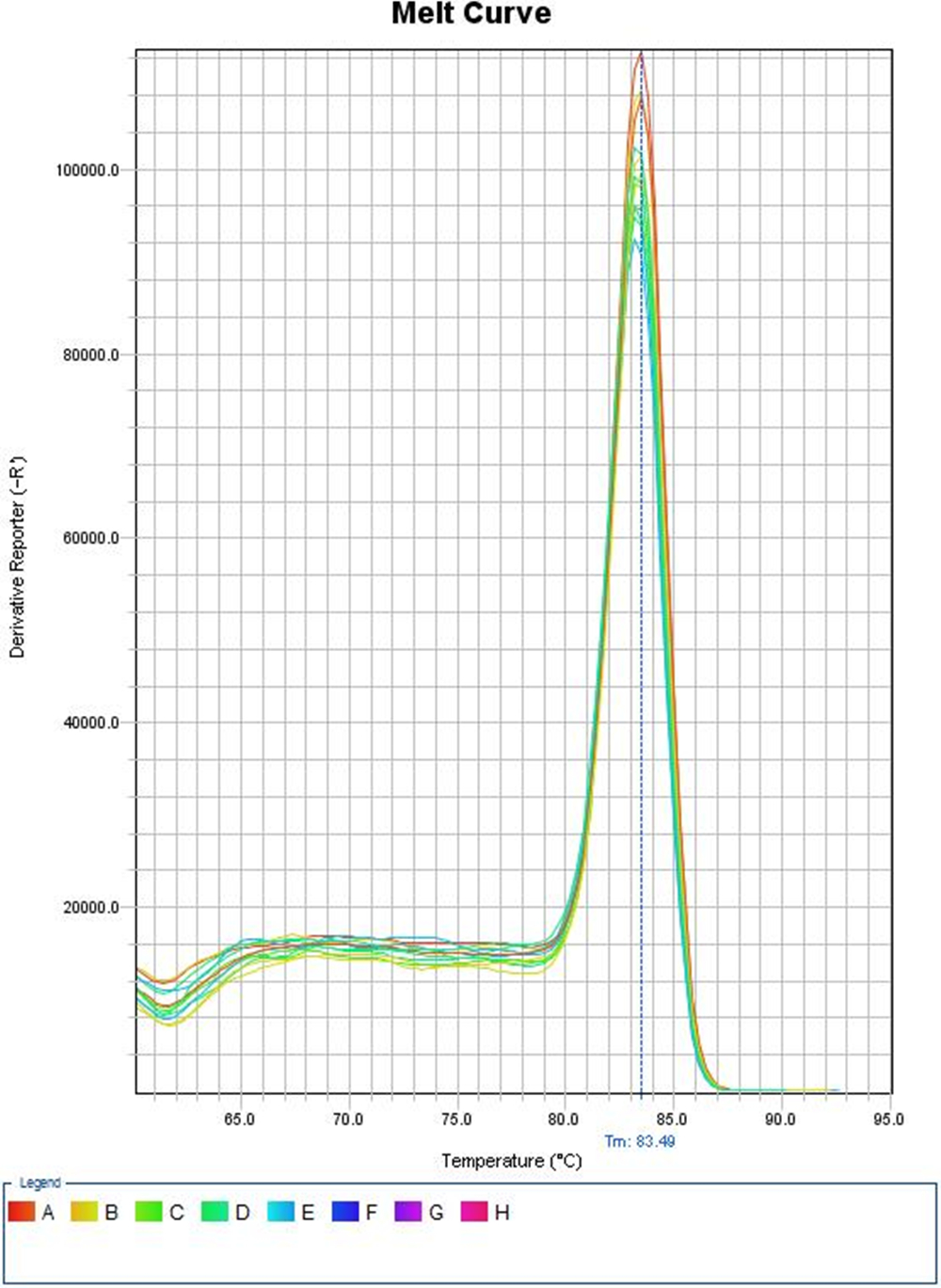** | **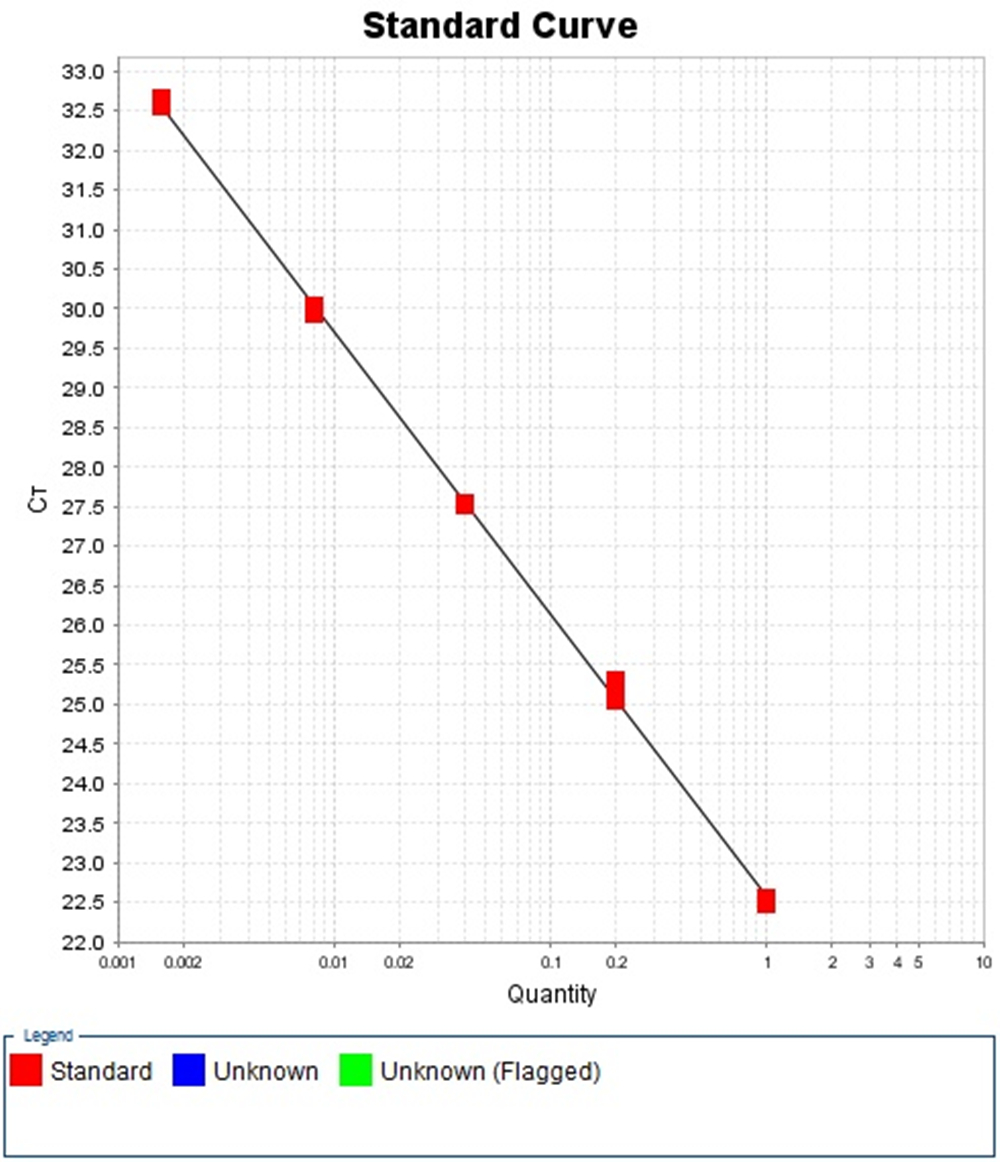** |
| ***EIF*** | **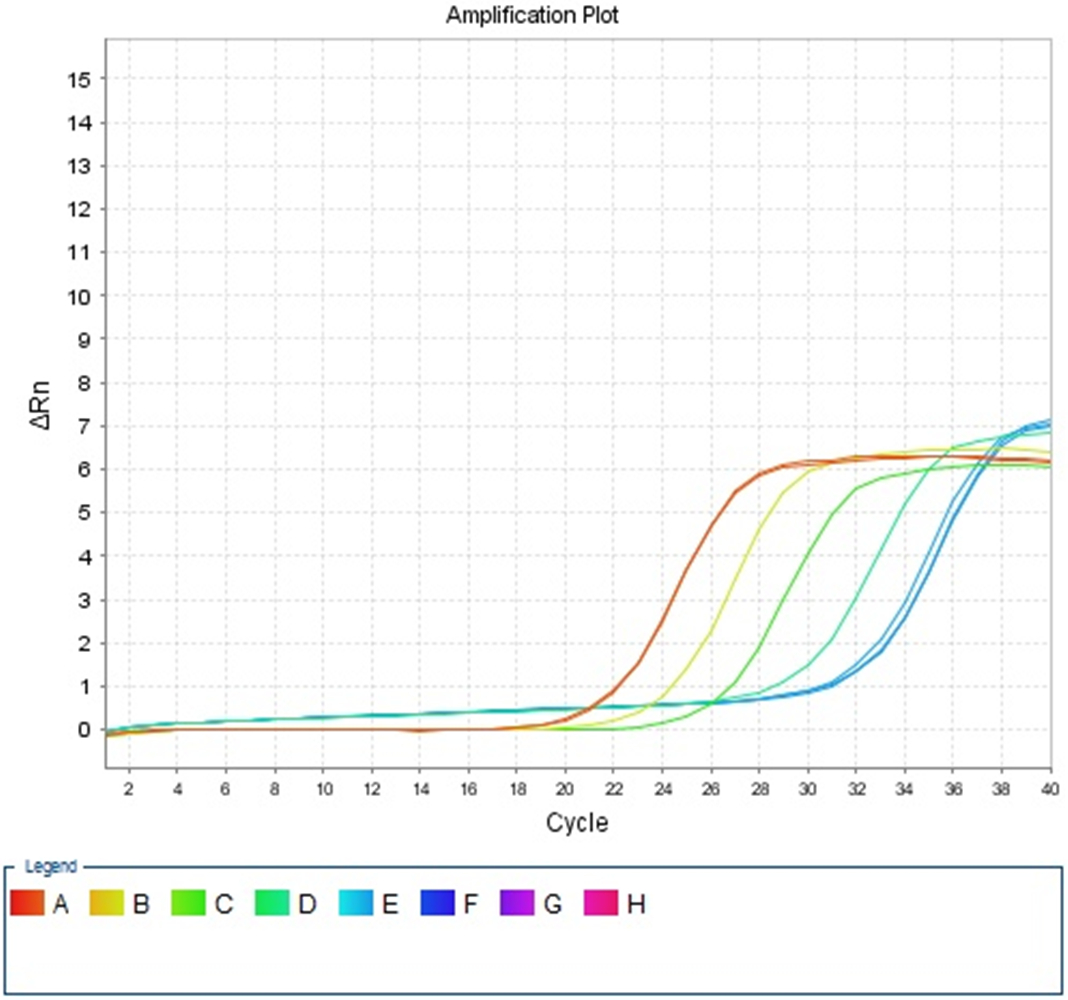** | **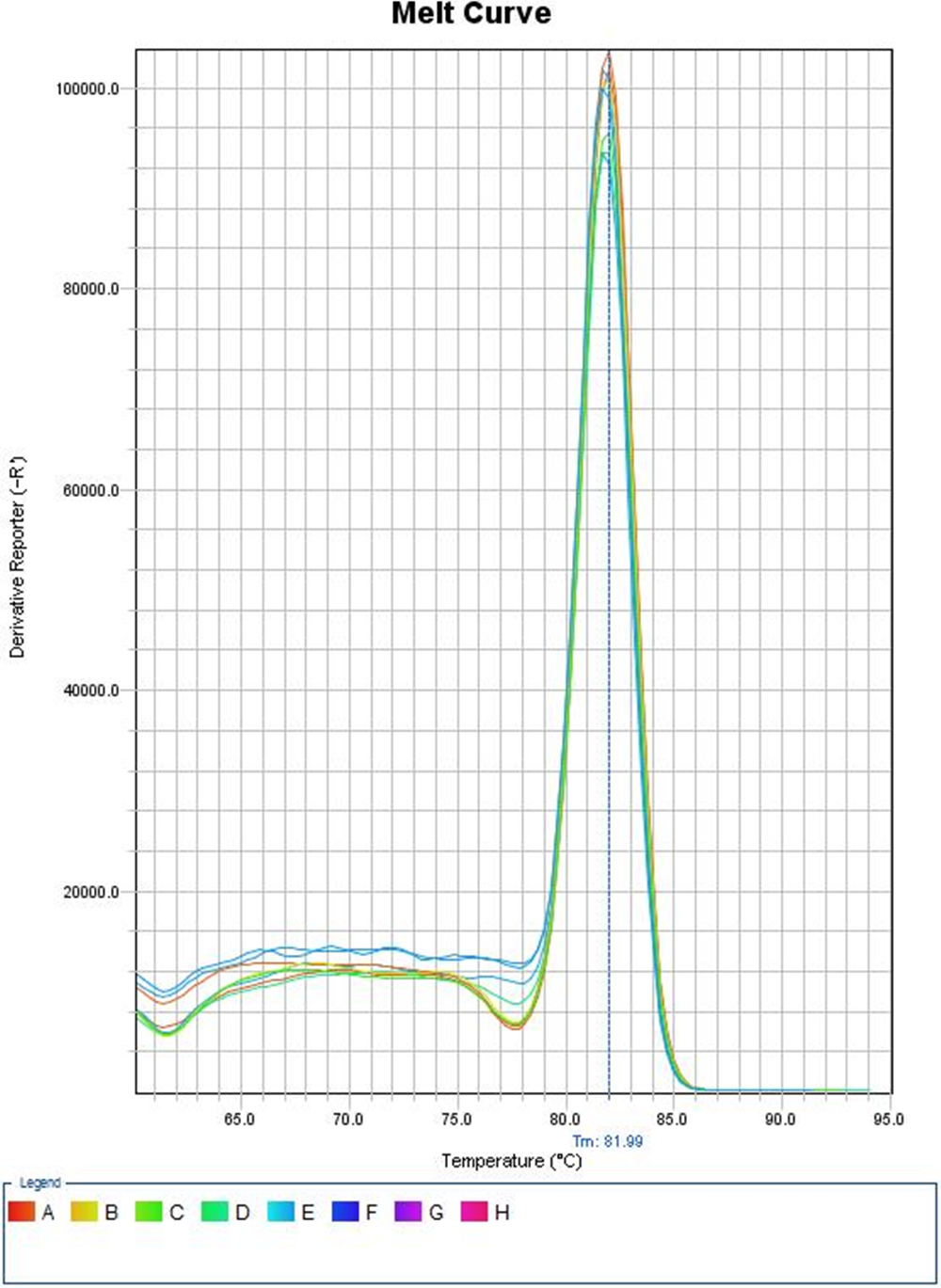** | **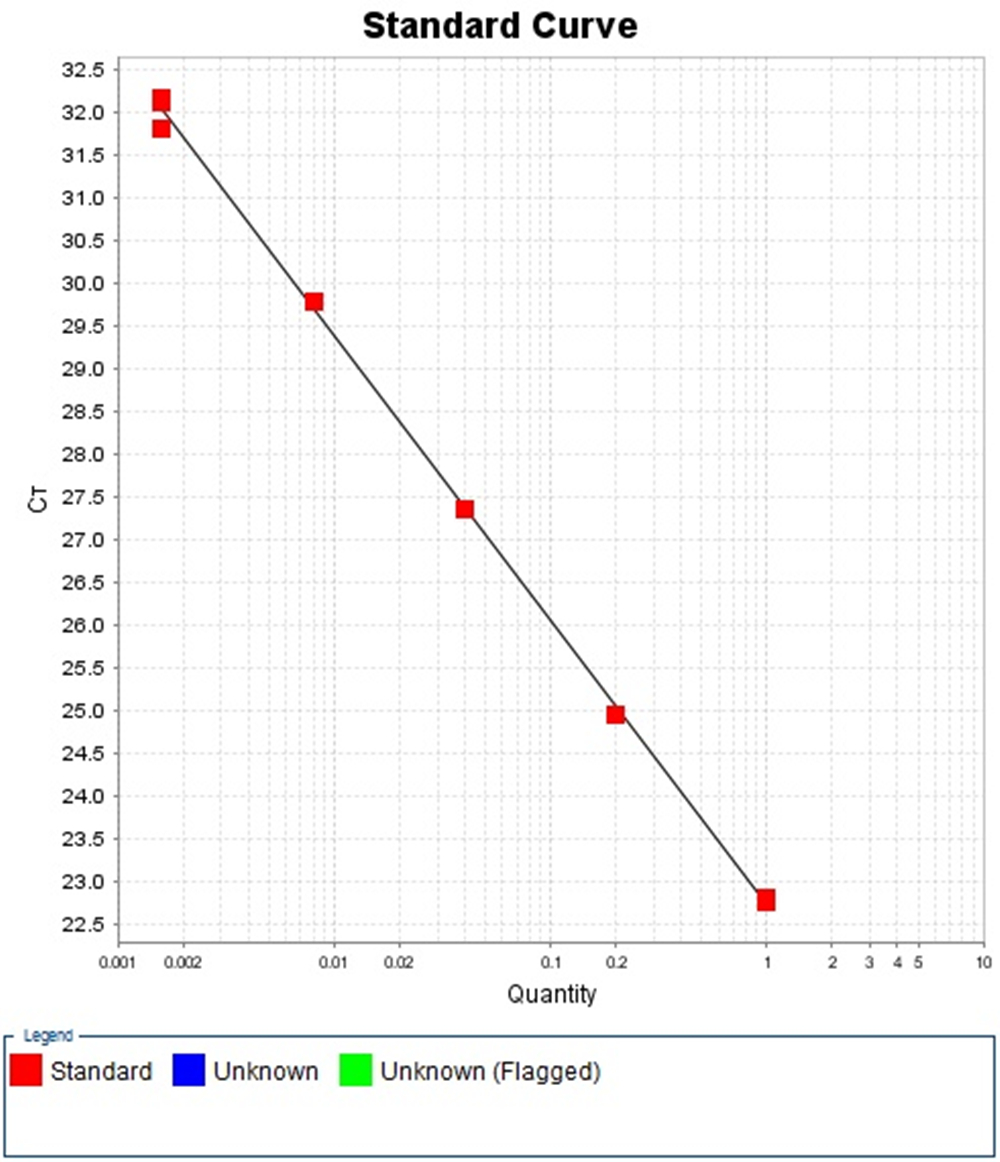** |
| ***Fbox*** | **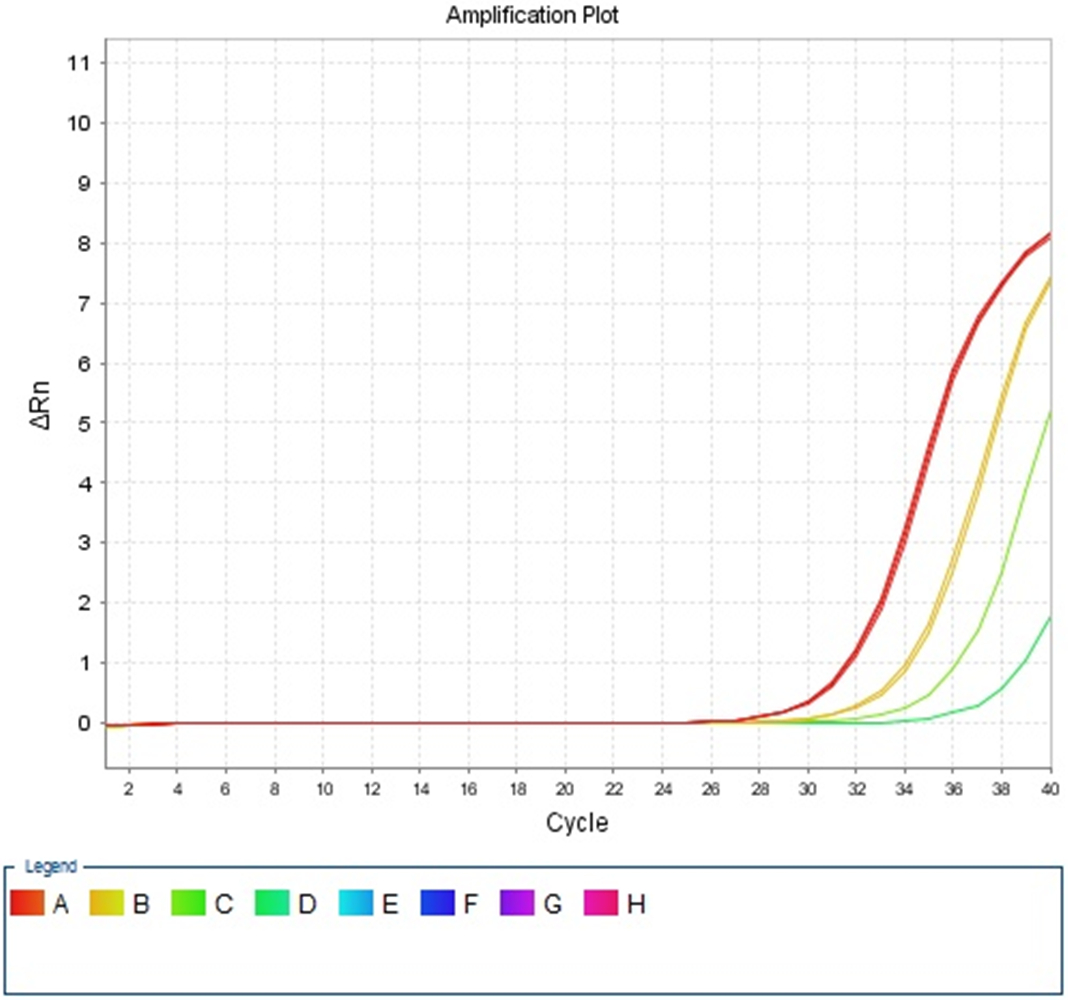** | **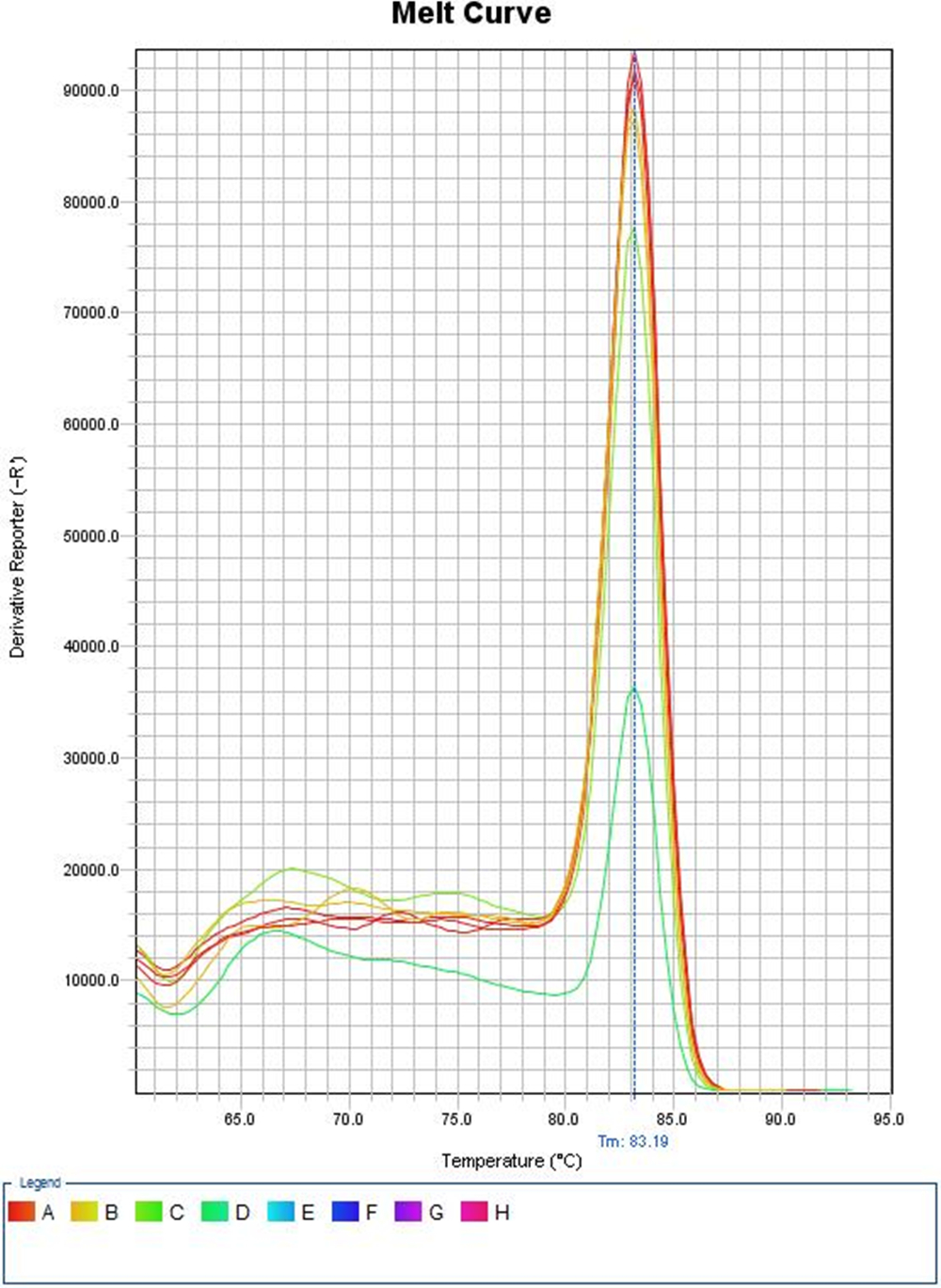** | **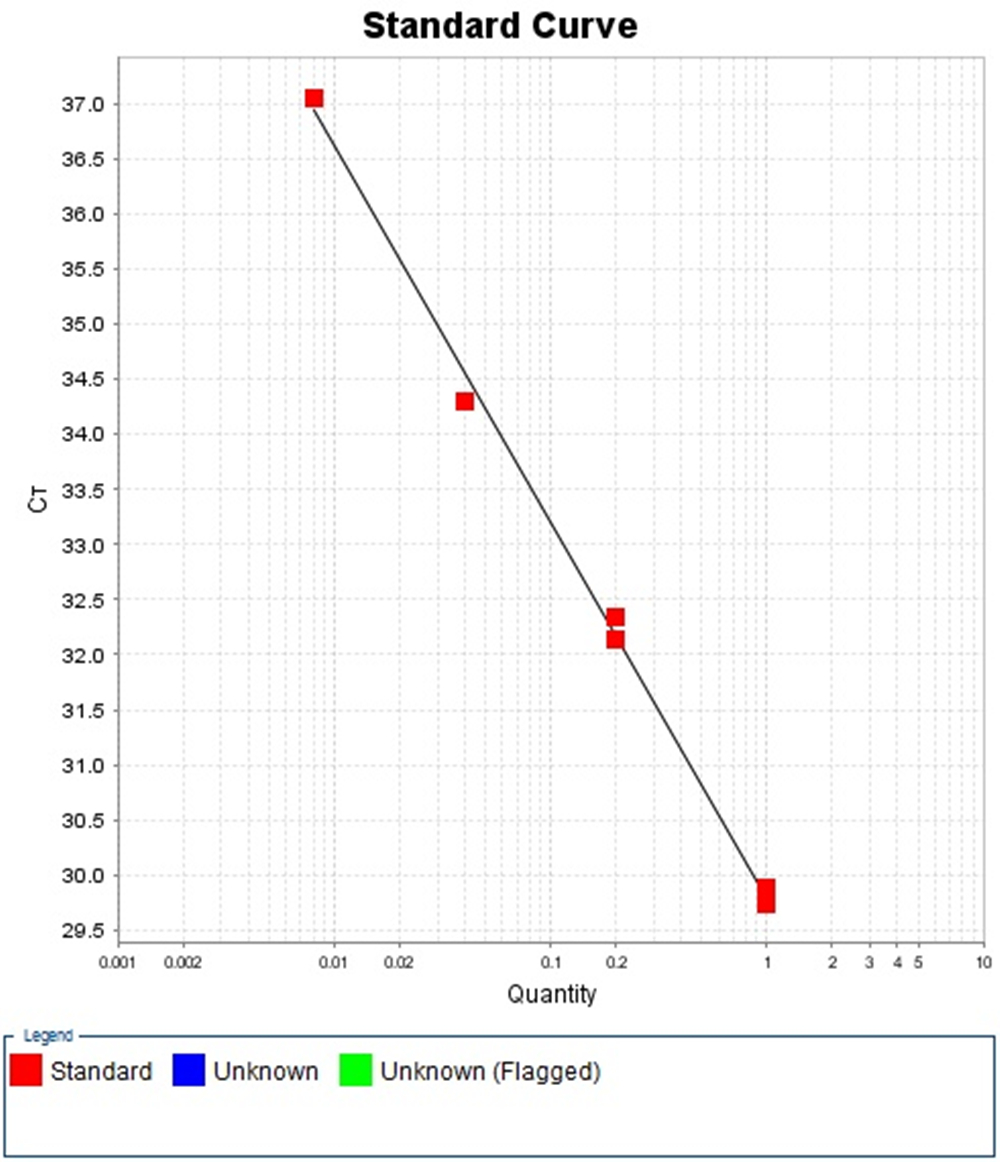** |
| ***FLD*** | **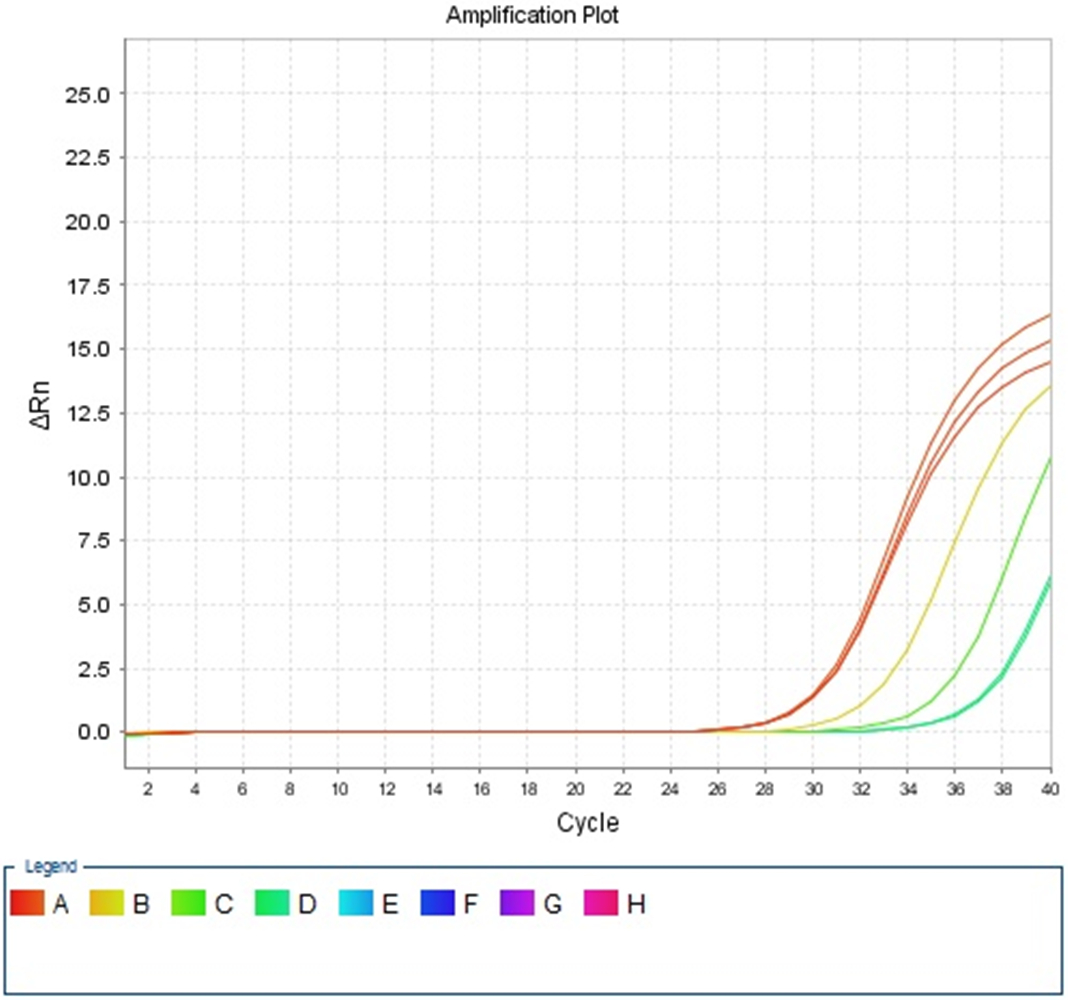** | **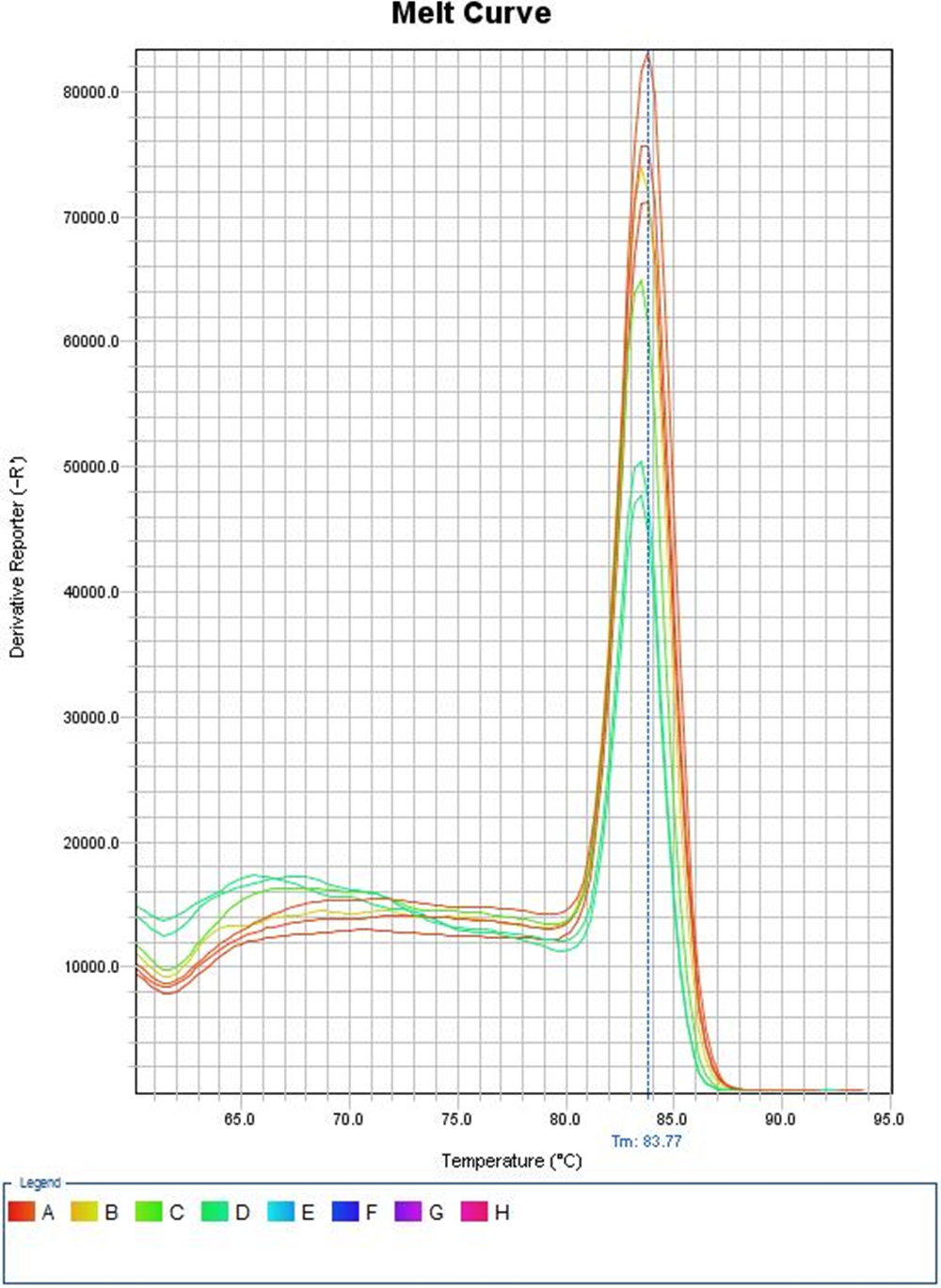** | **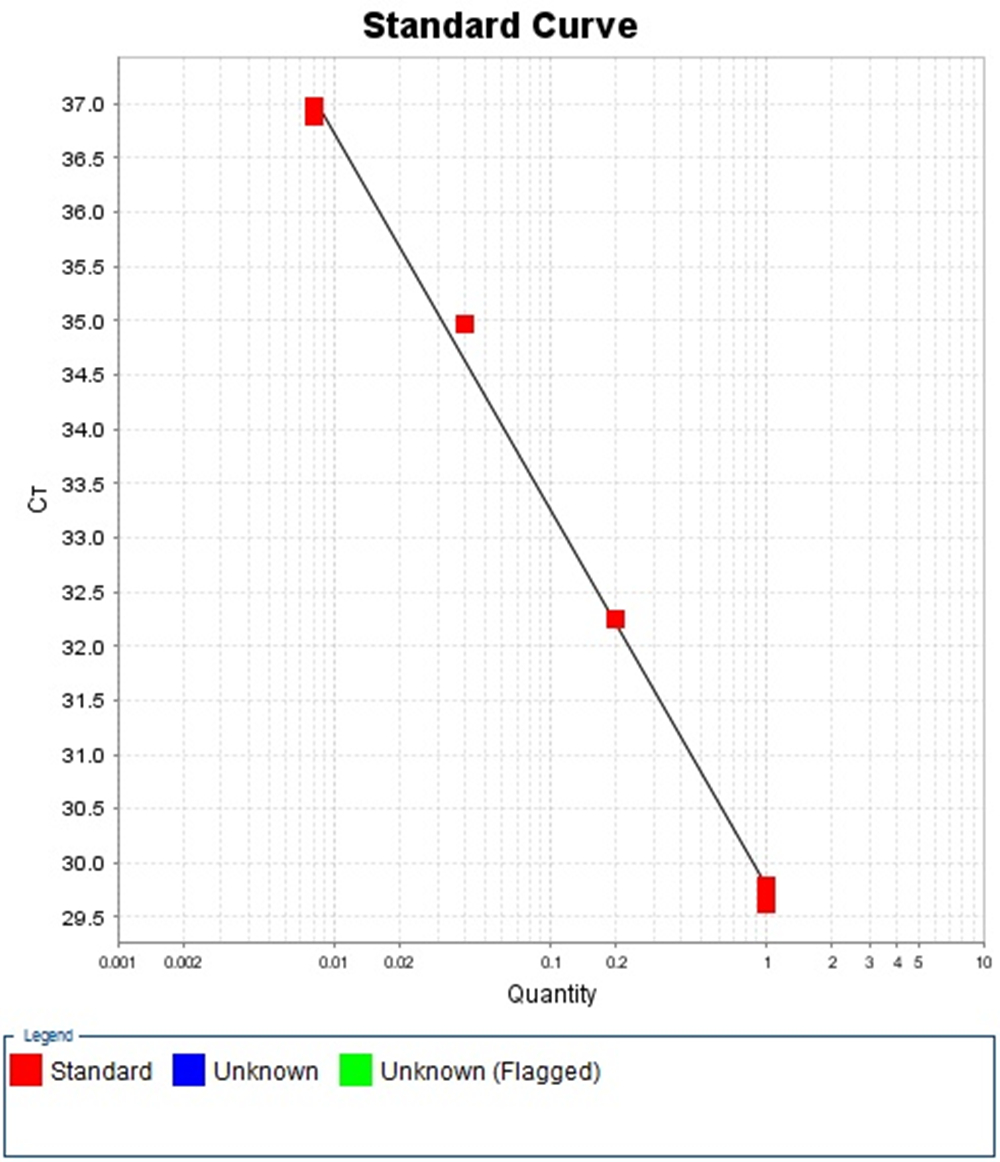** |
| ***GAPDH*** | **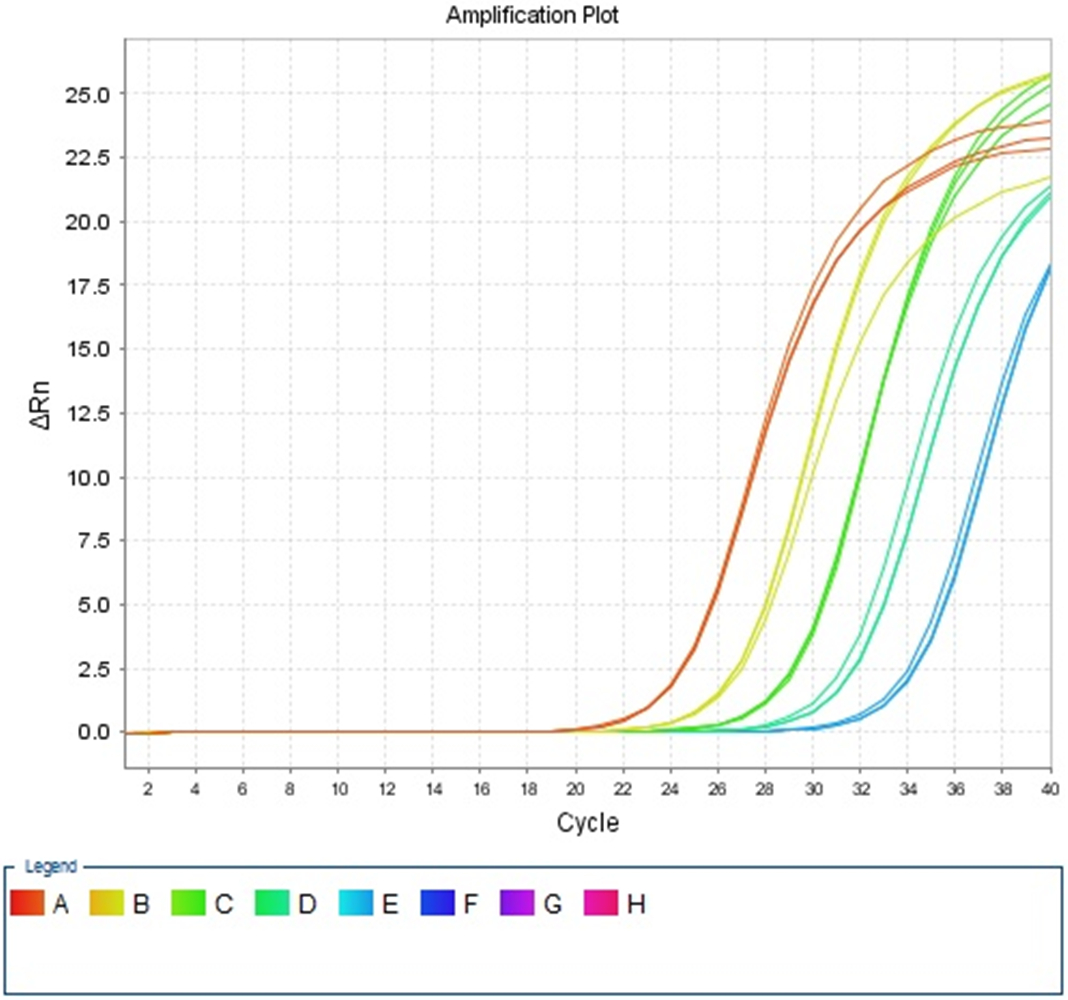** | **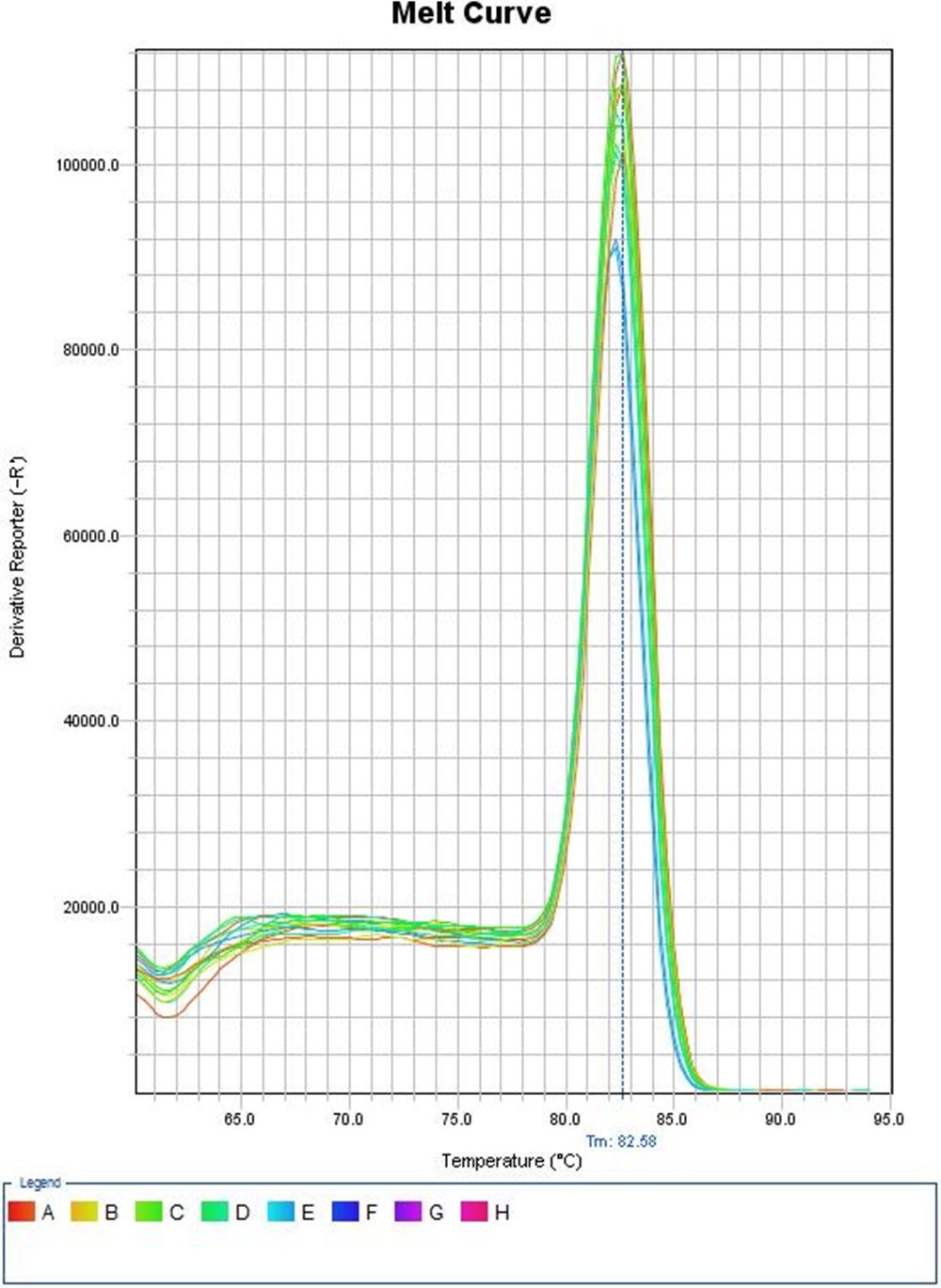** | **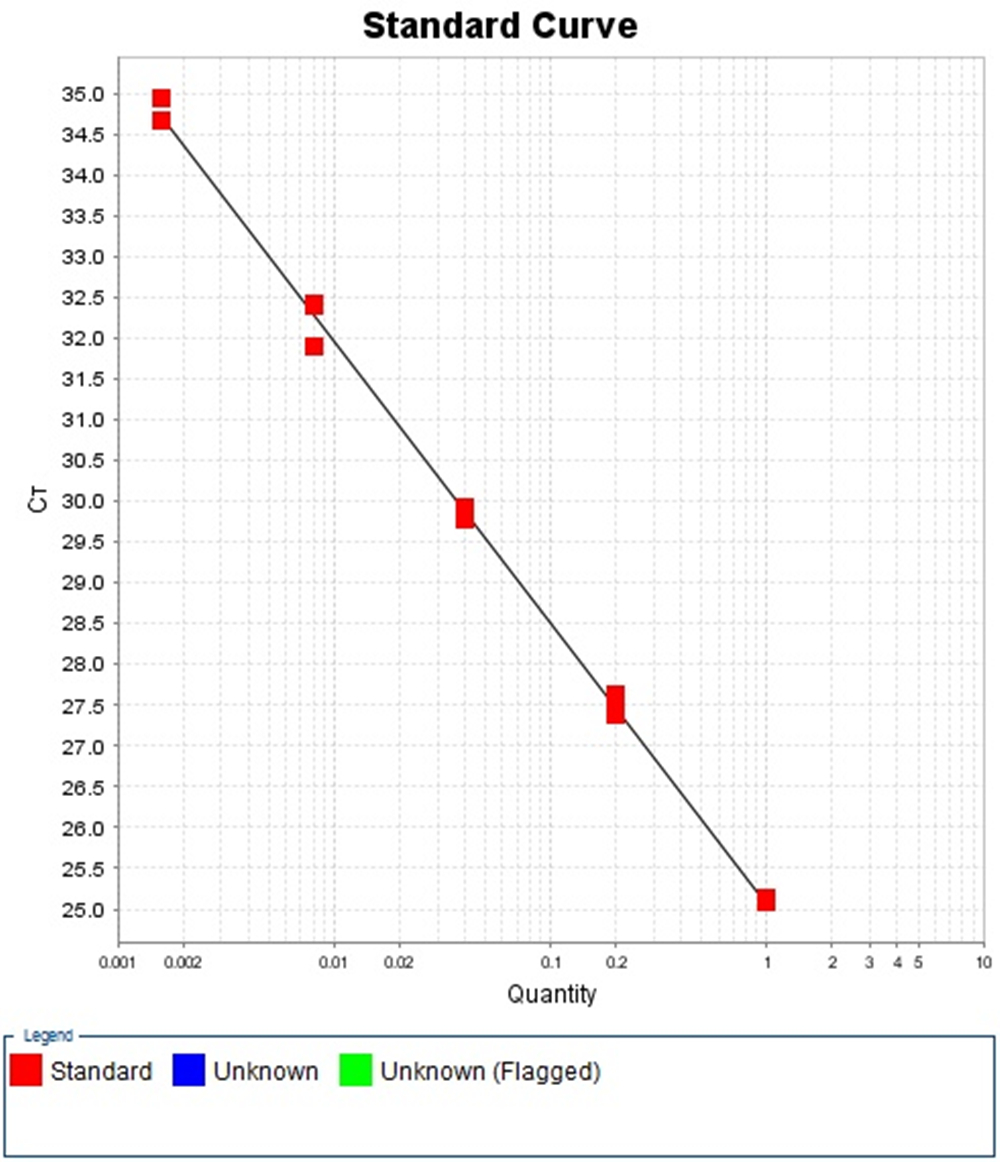** |
| ***HIS*** | **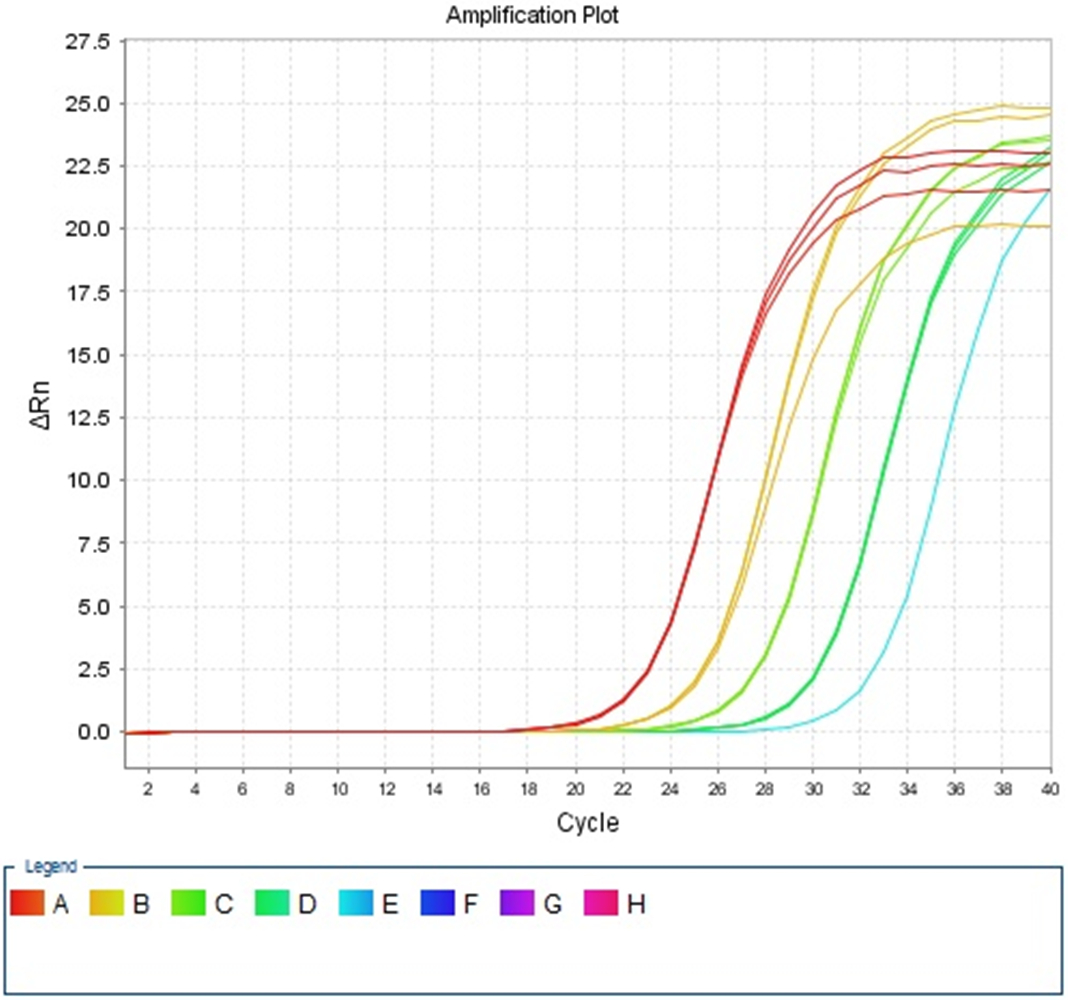** | **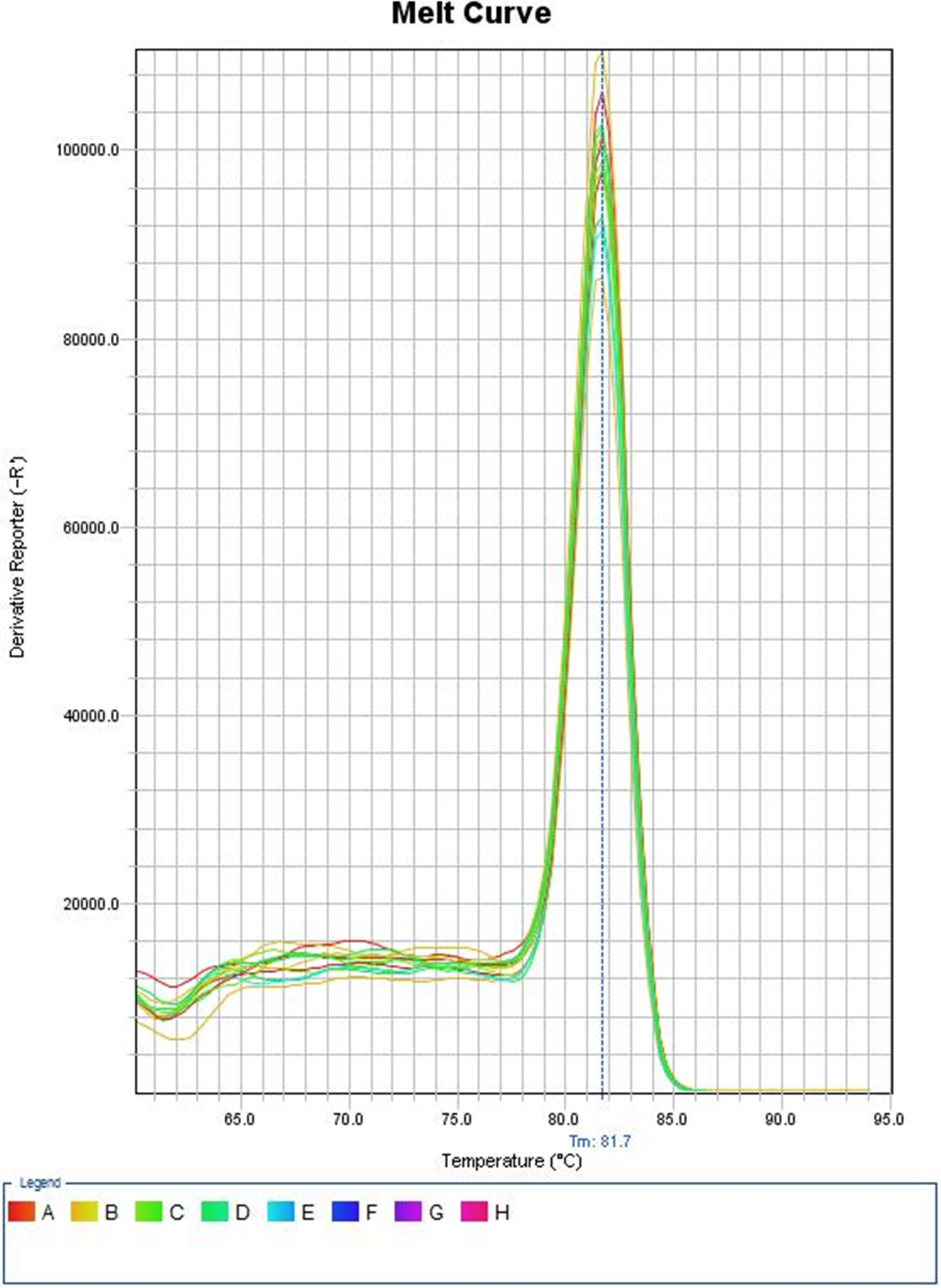** | **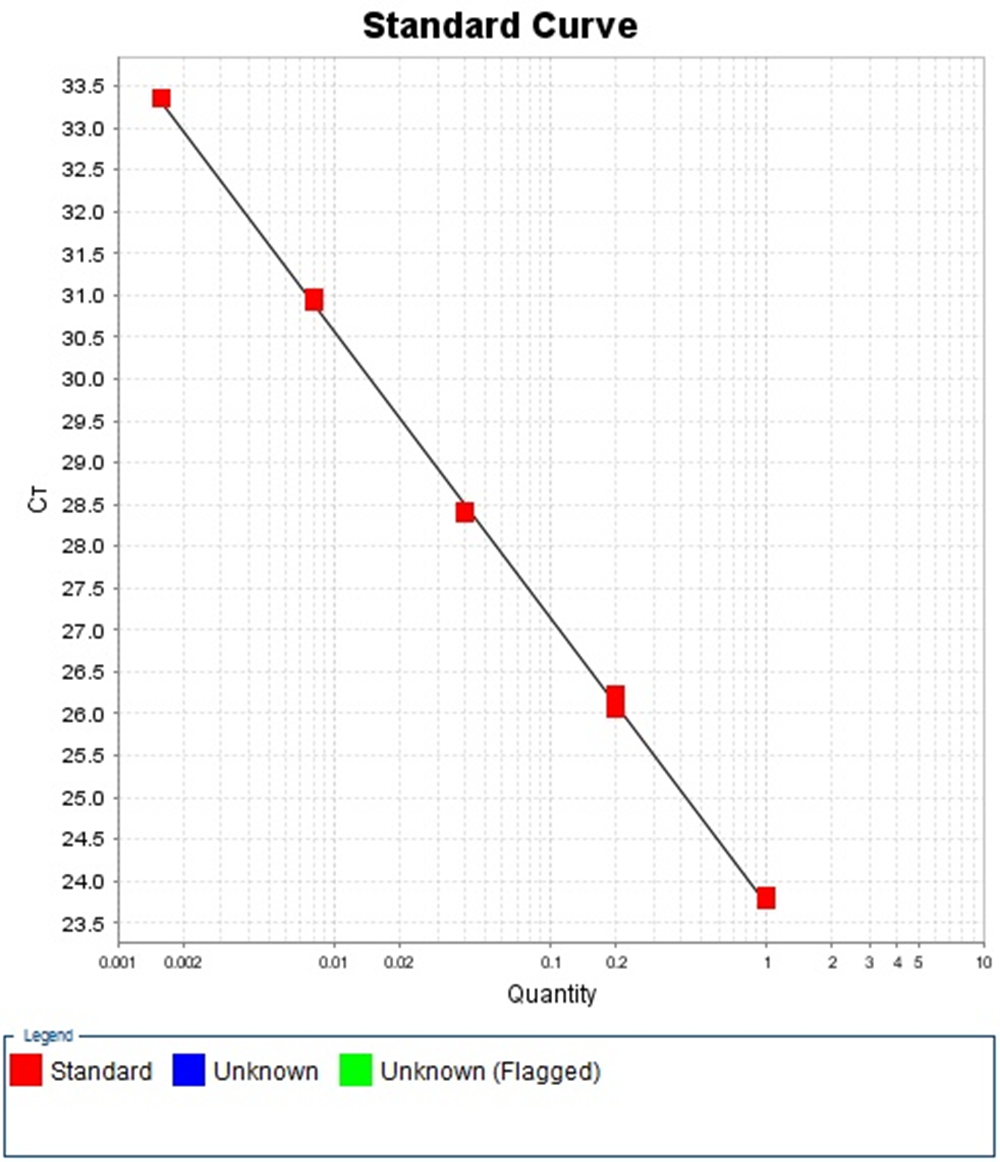** |
| ***PP2A*** | 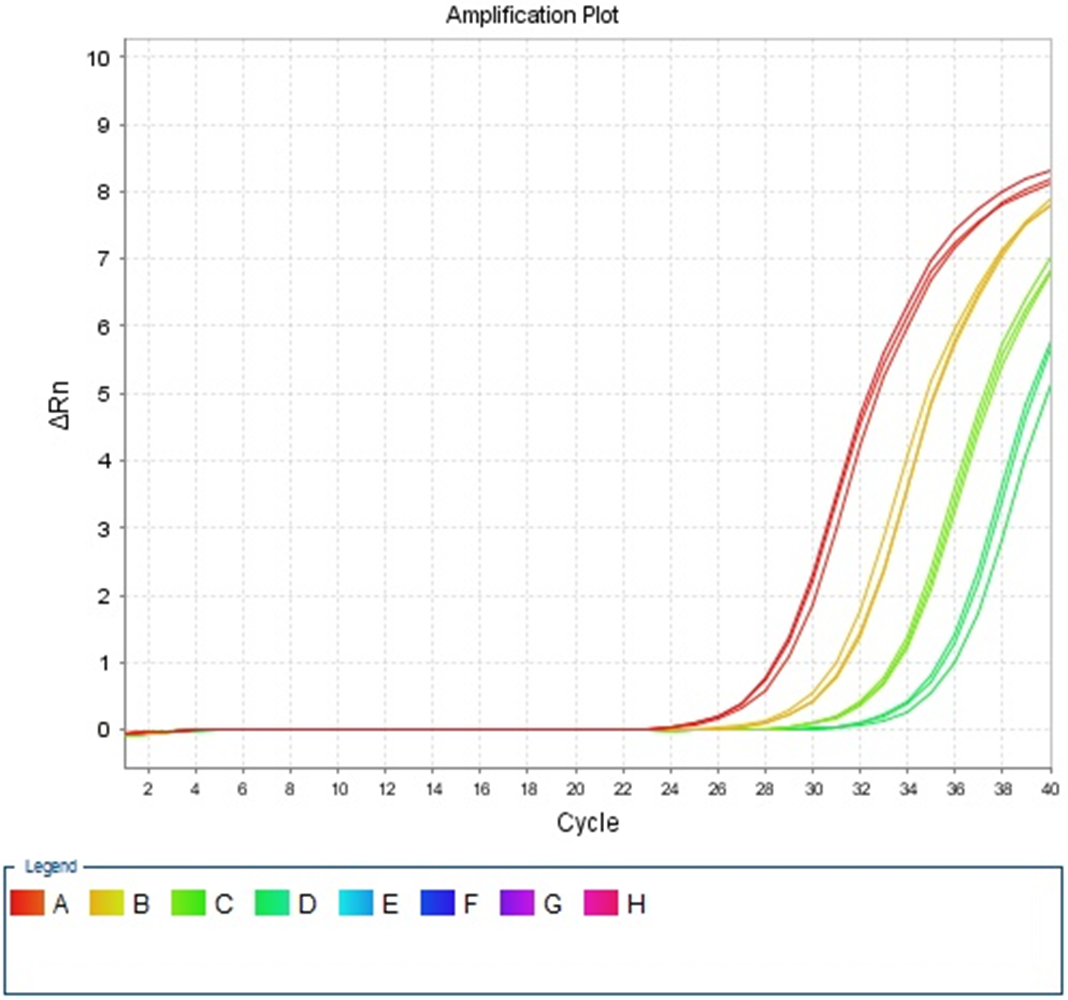 | 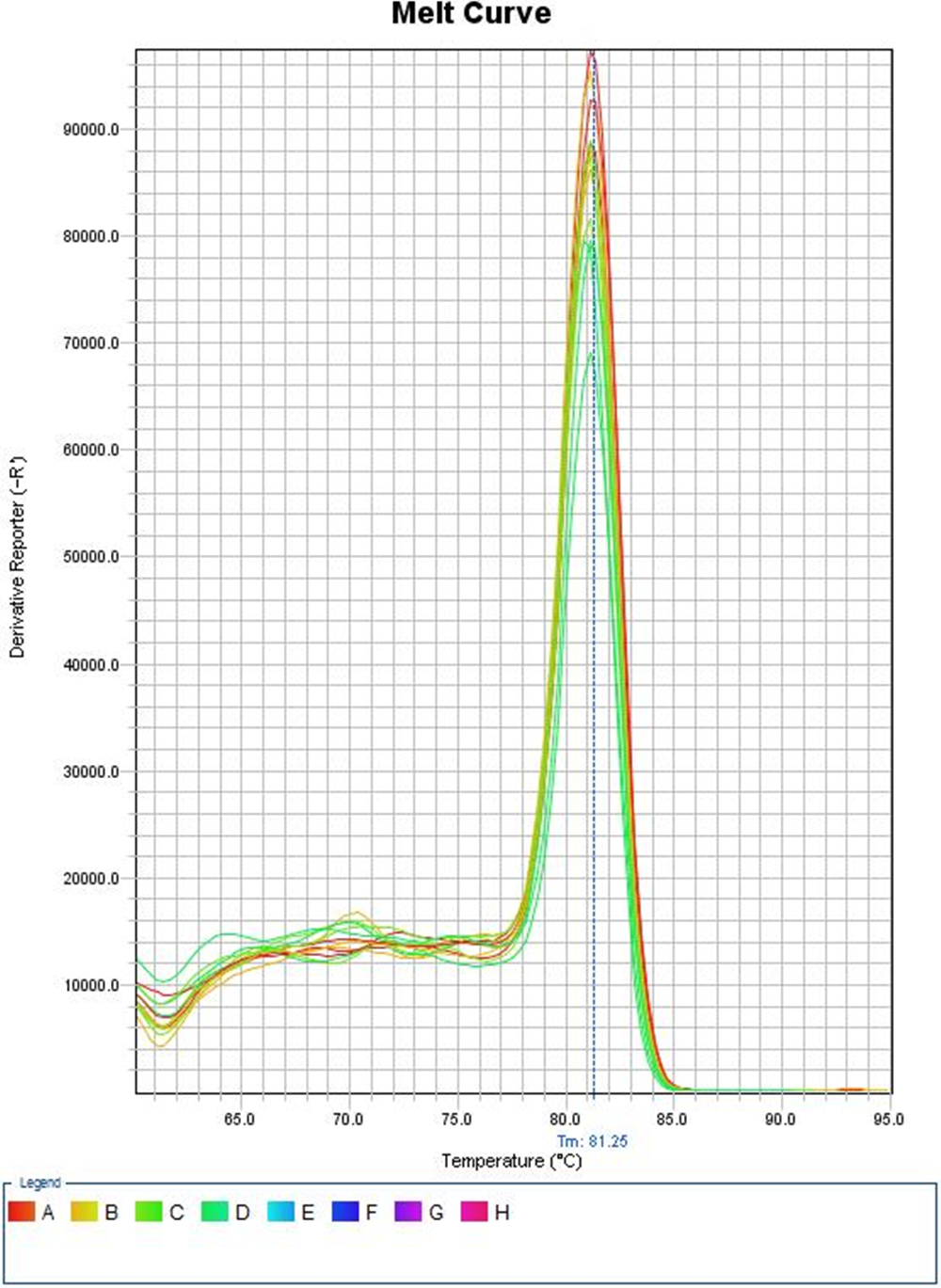 | 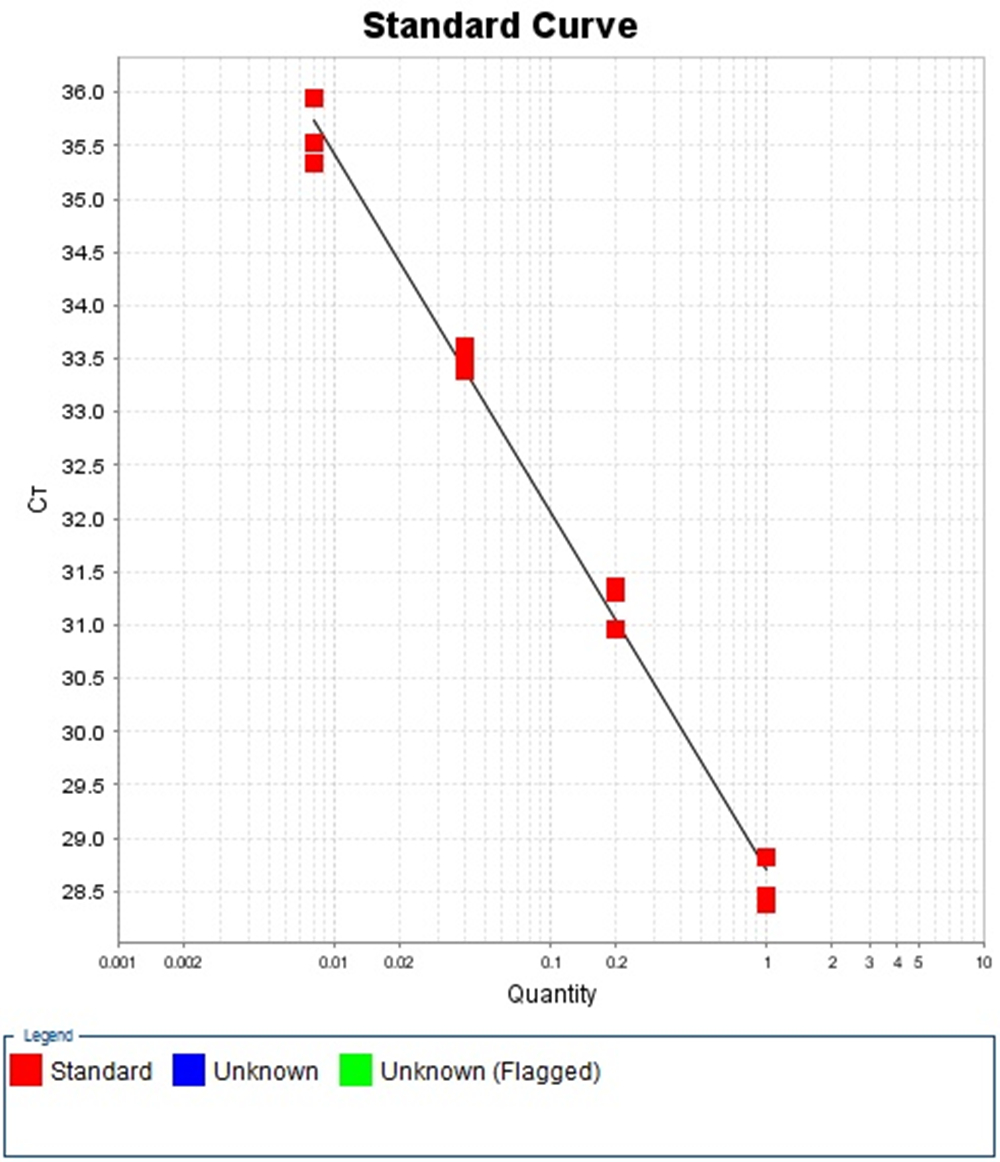 |
| ***RP*** | **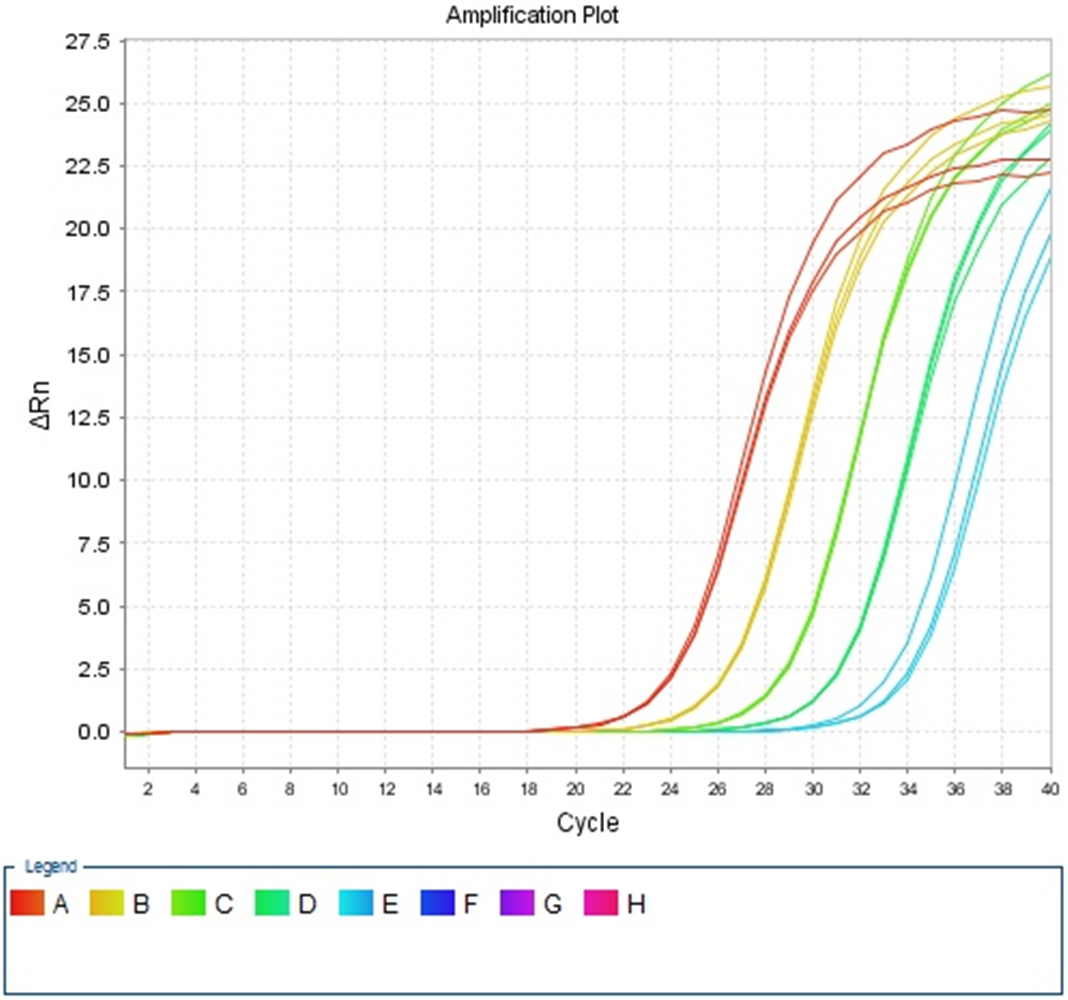** | **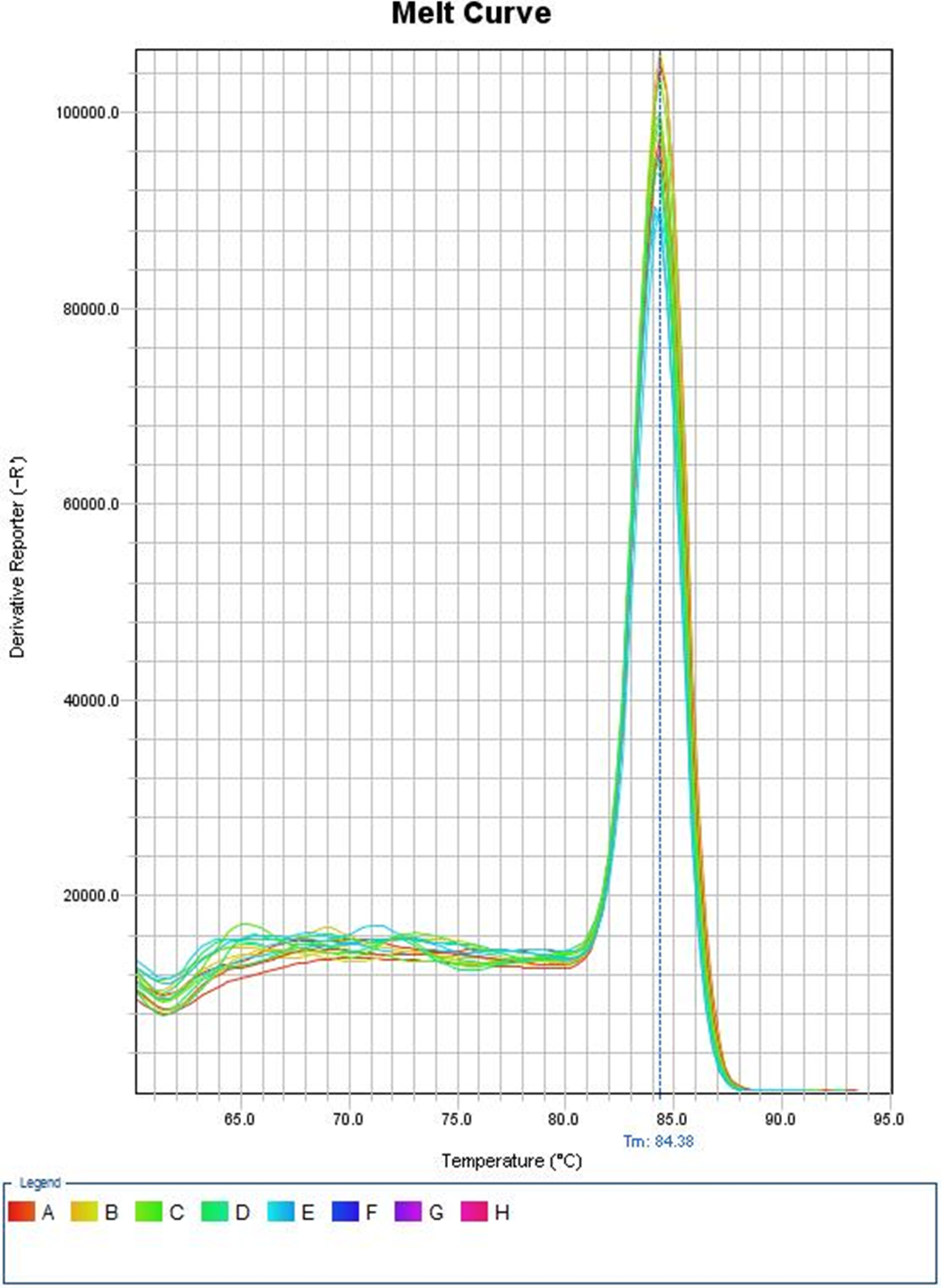** | **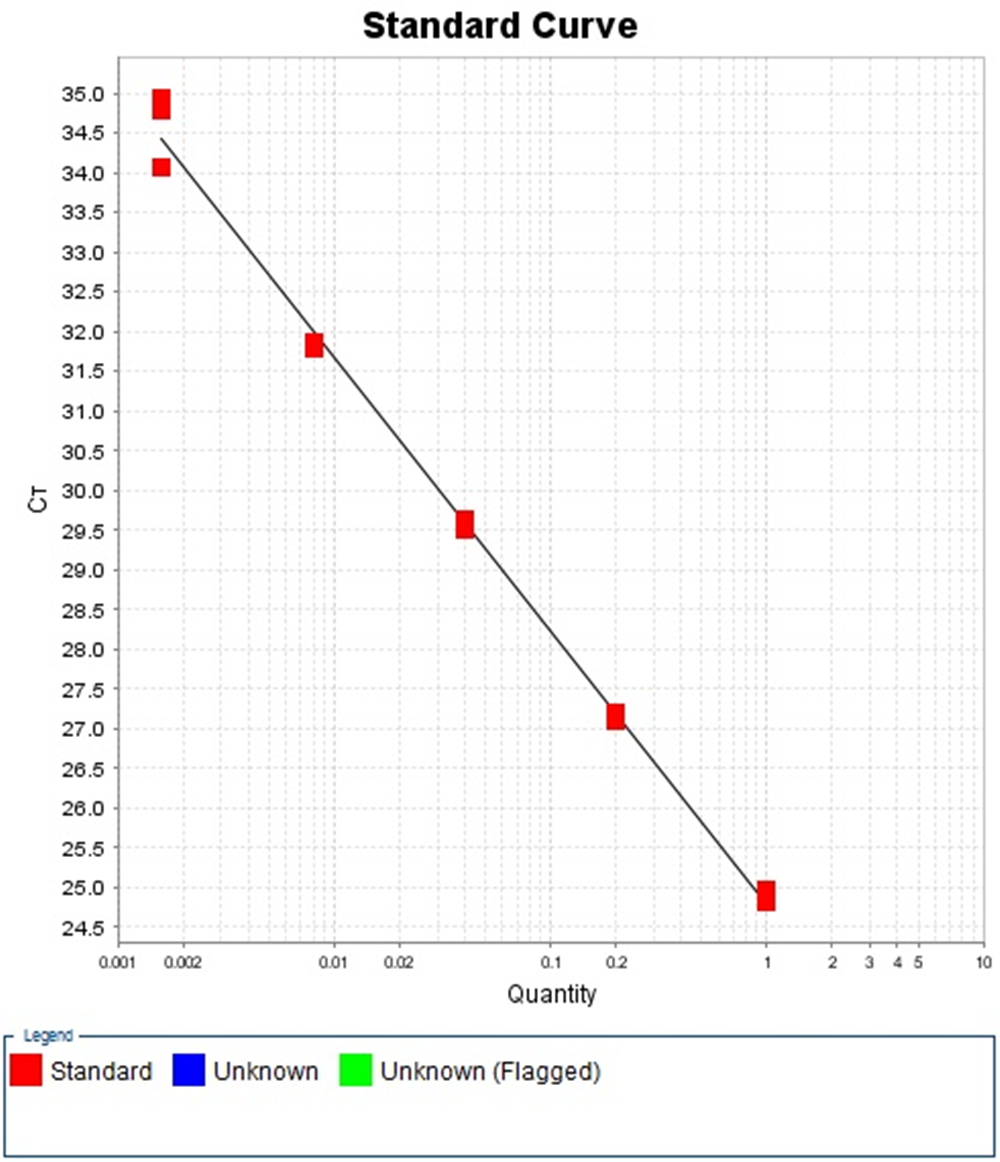** |
| ***SAND*** | **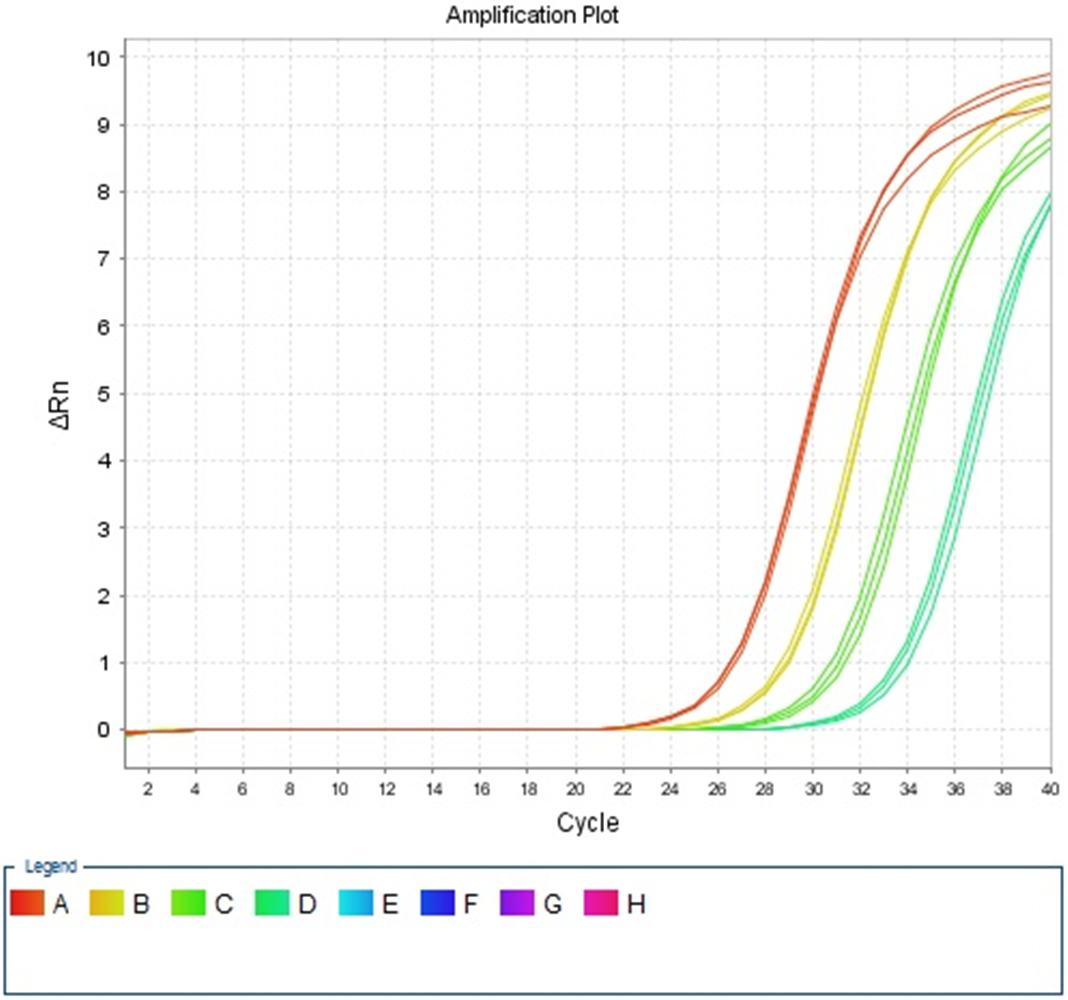** | **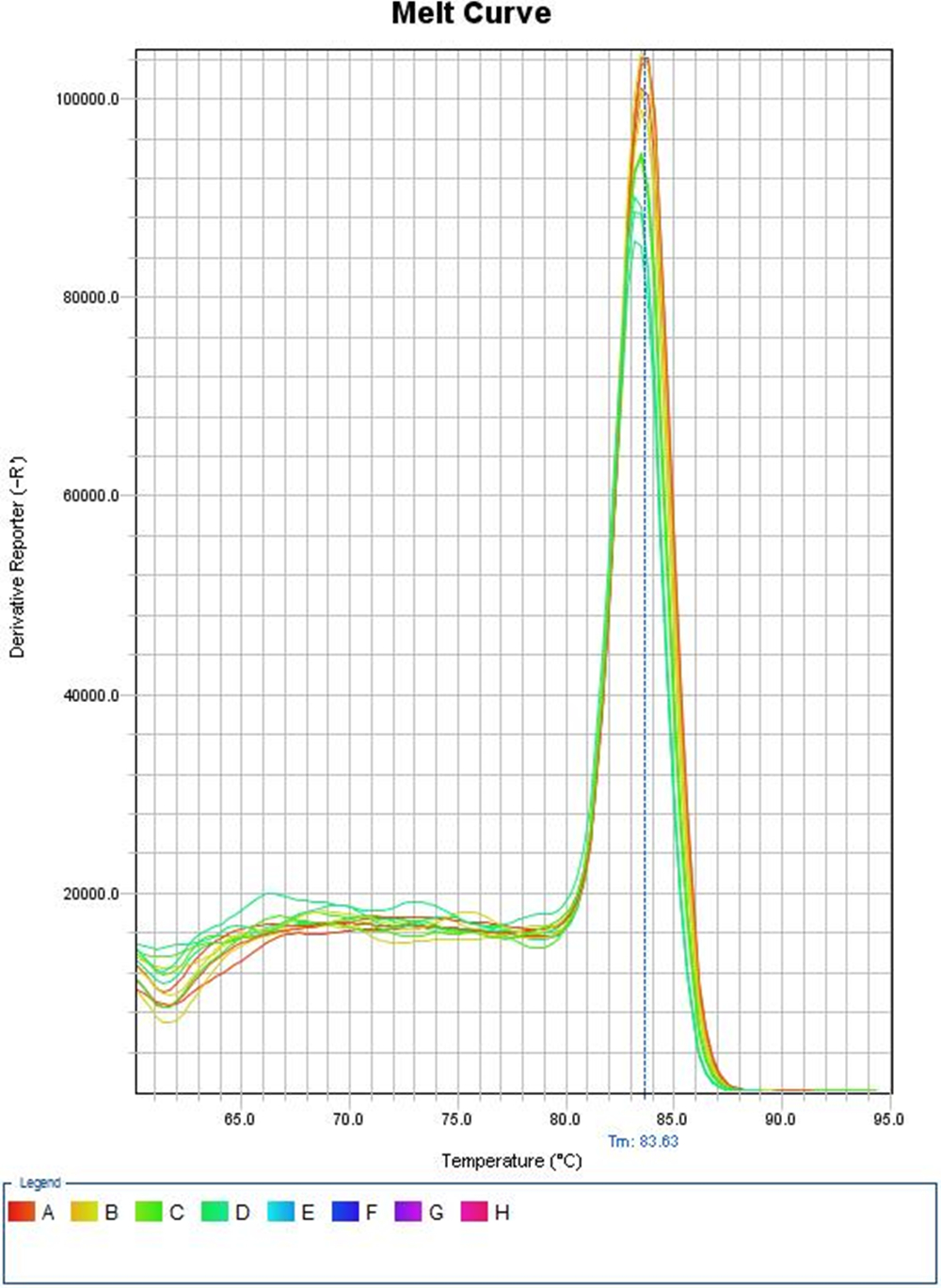** | **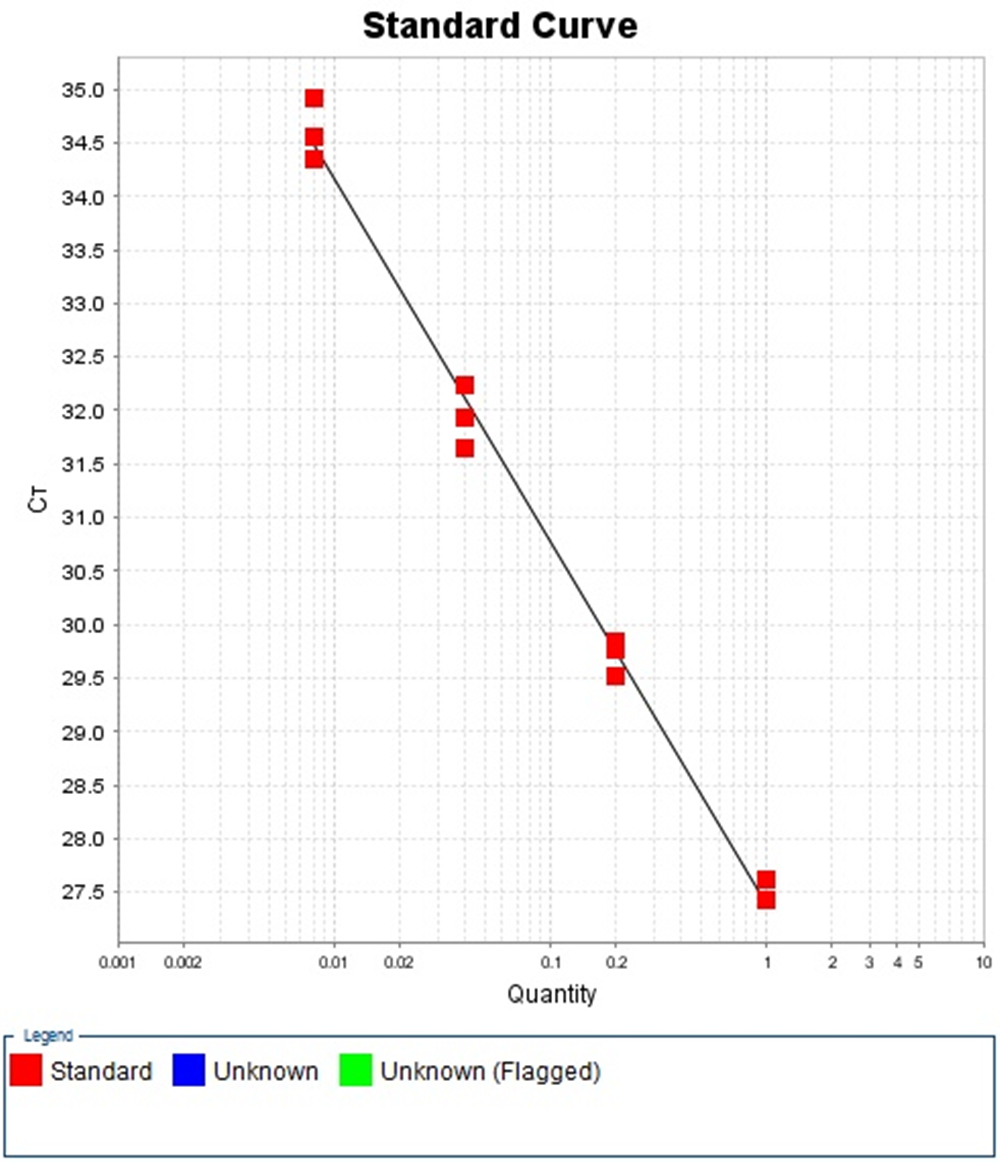** |
| ***TBP*** | **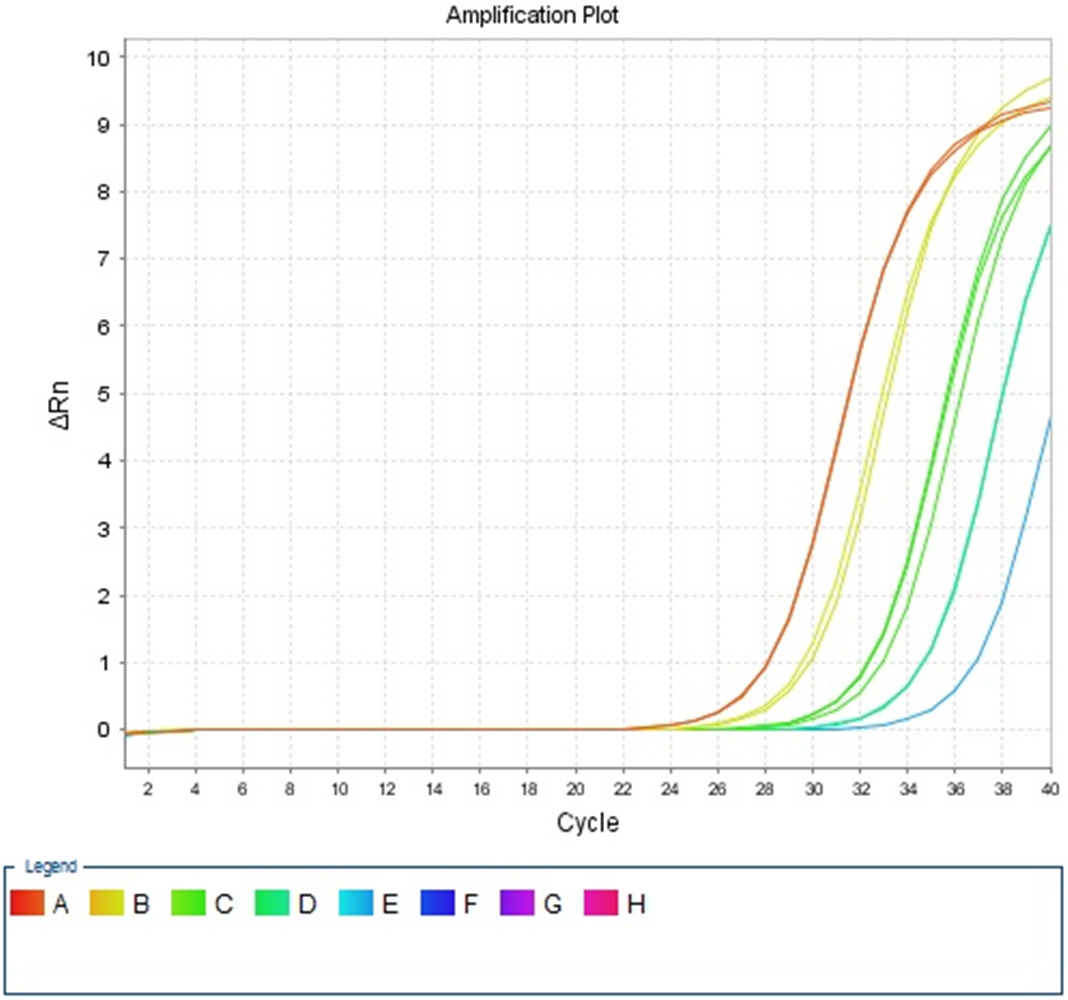** | **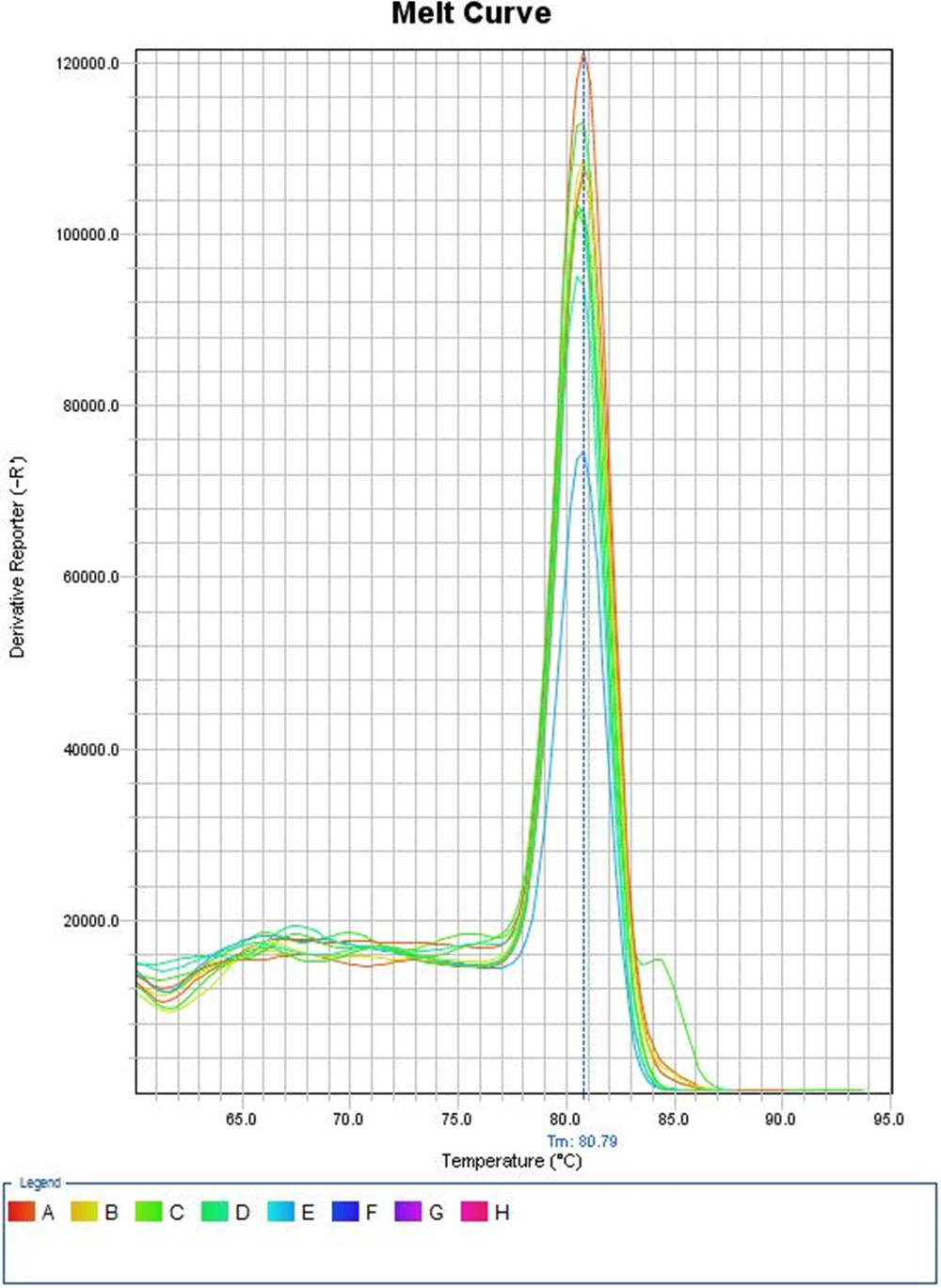** | **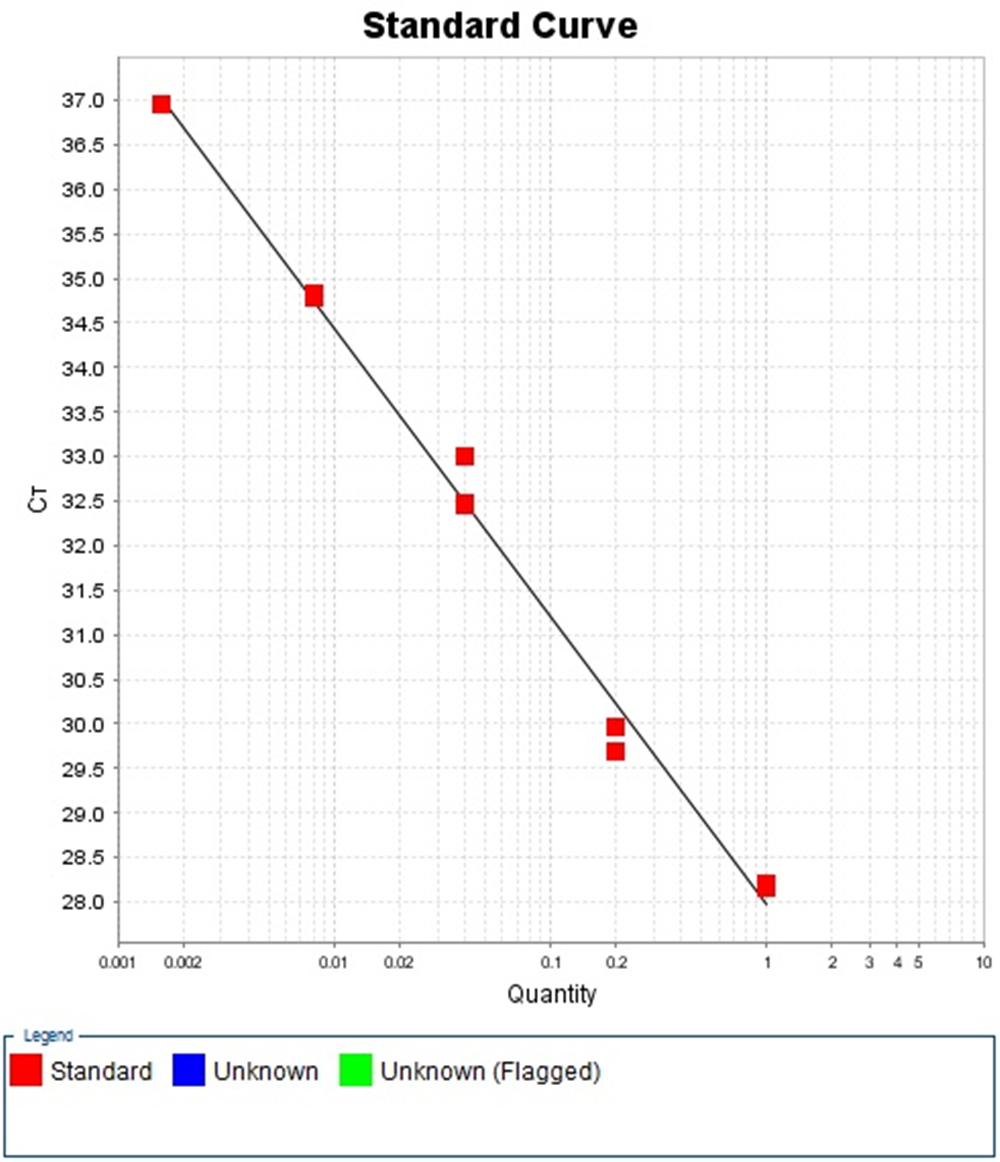** |
| ***TUB*** | **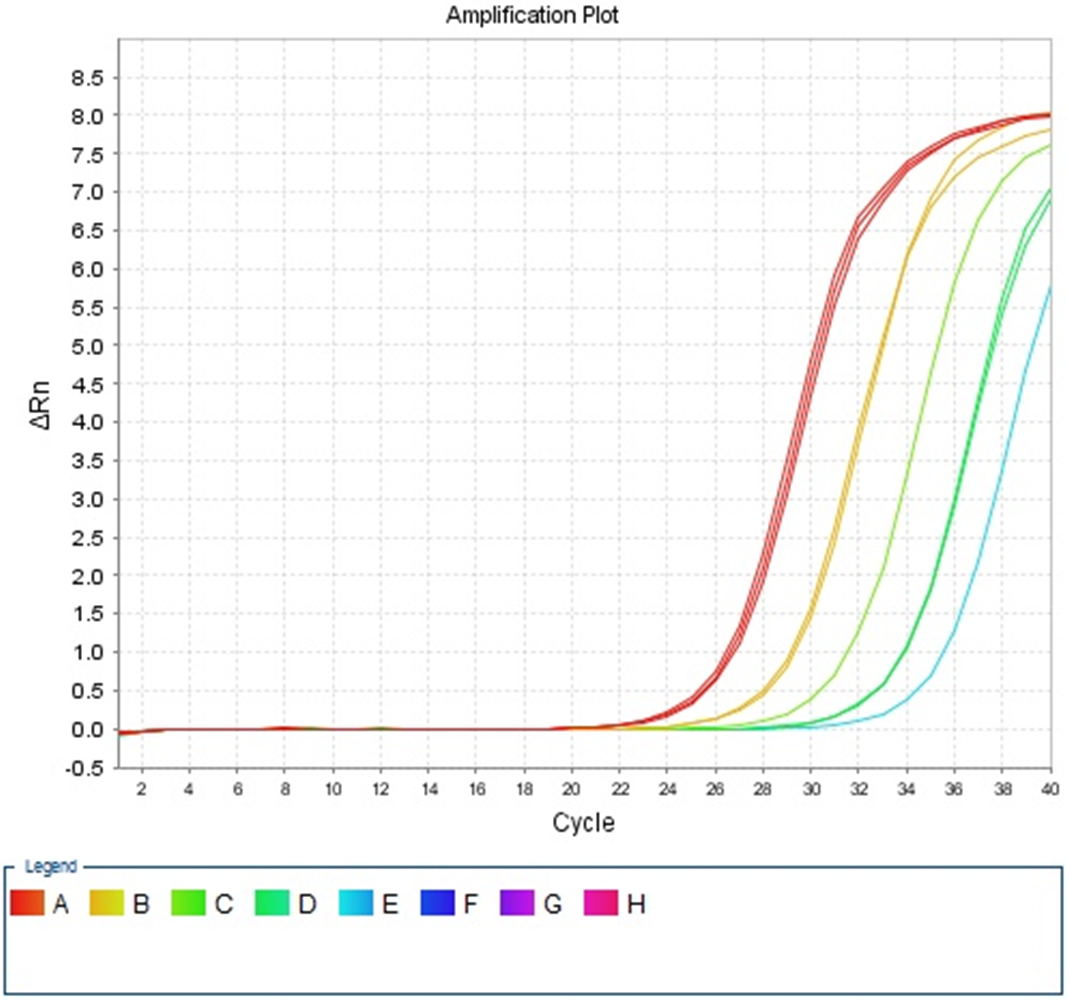** | **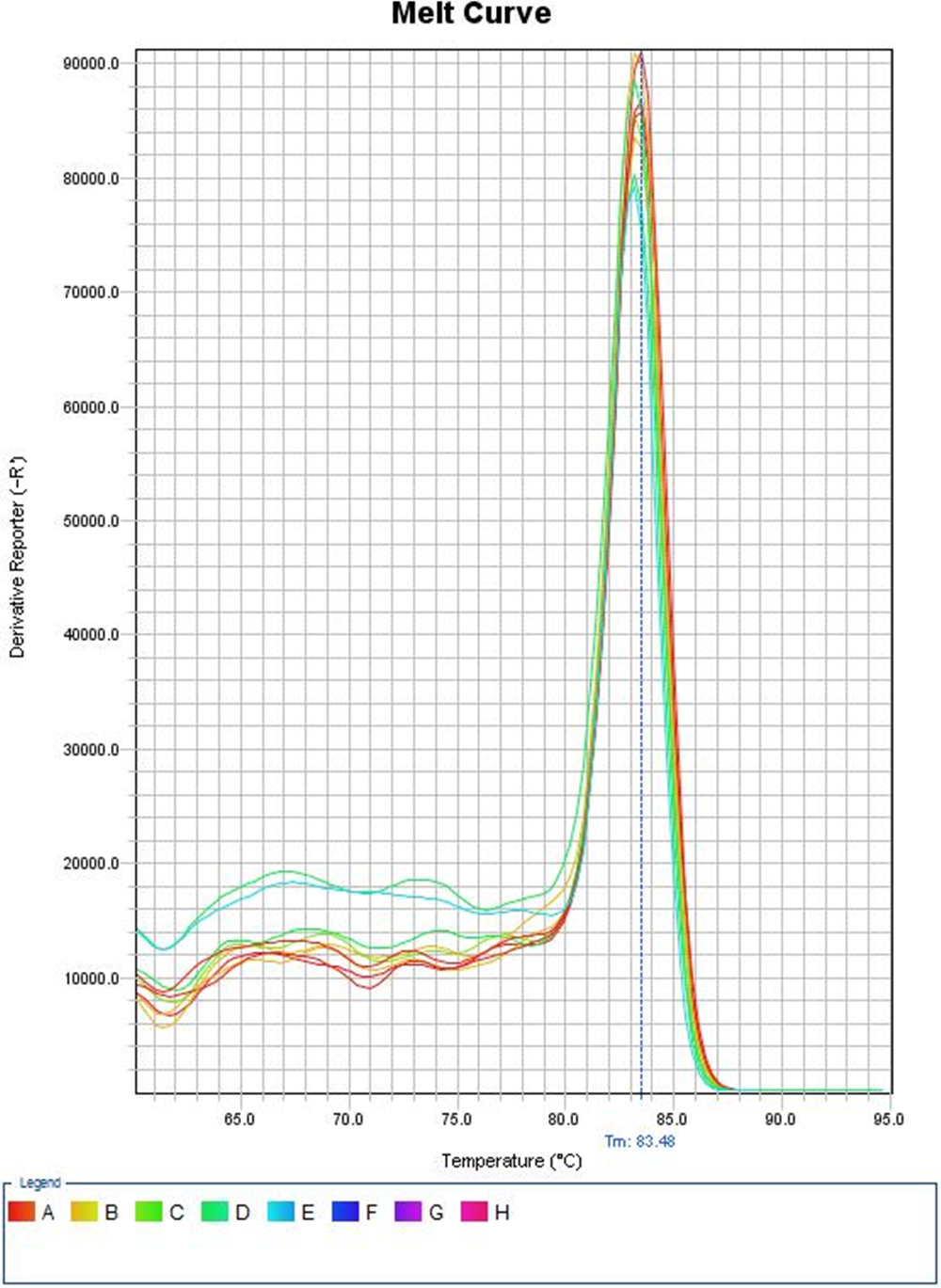** | **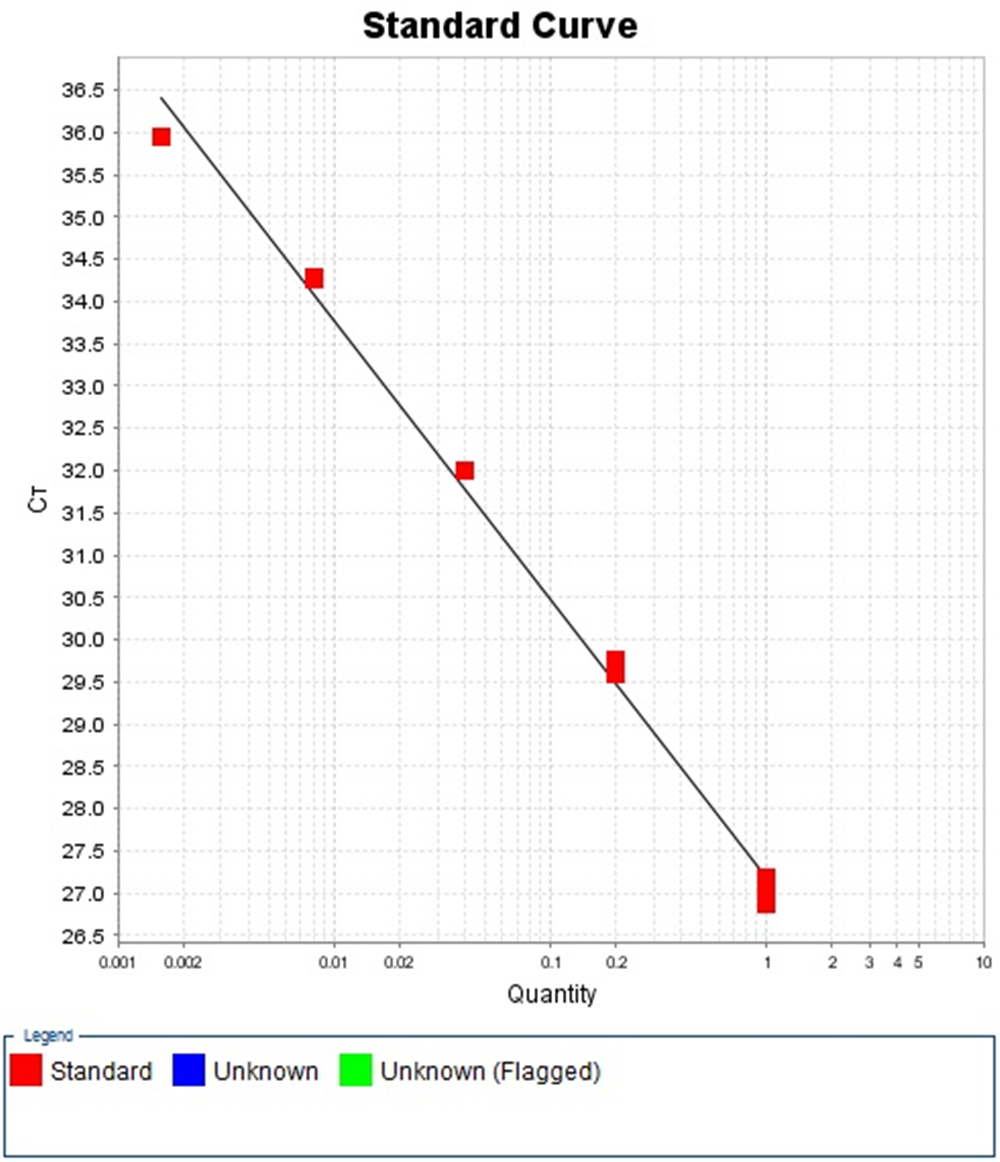** |
| ***UBCE*** | **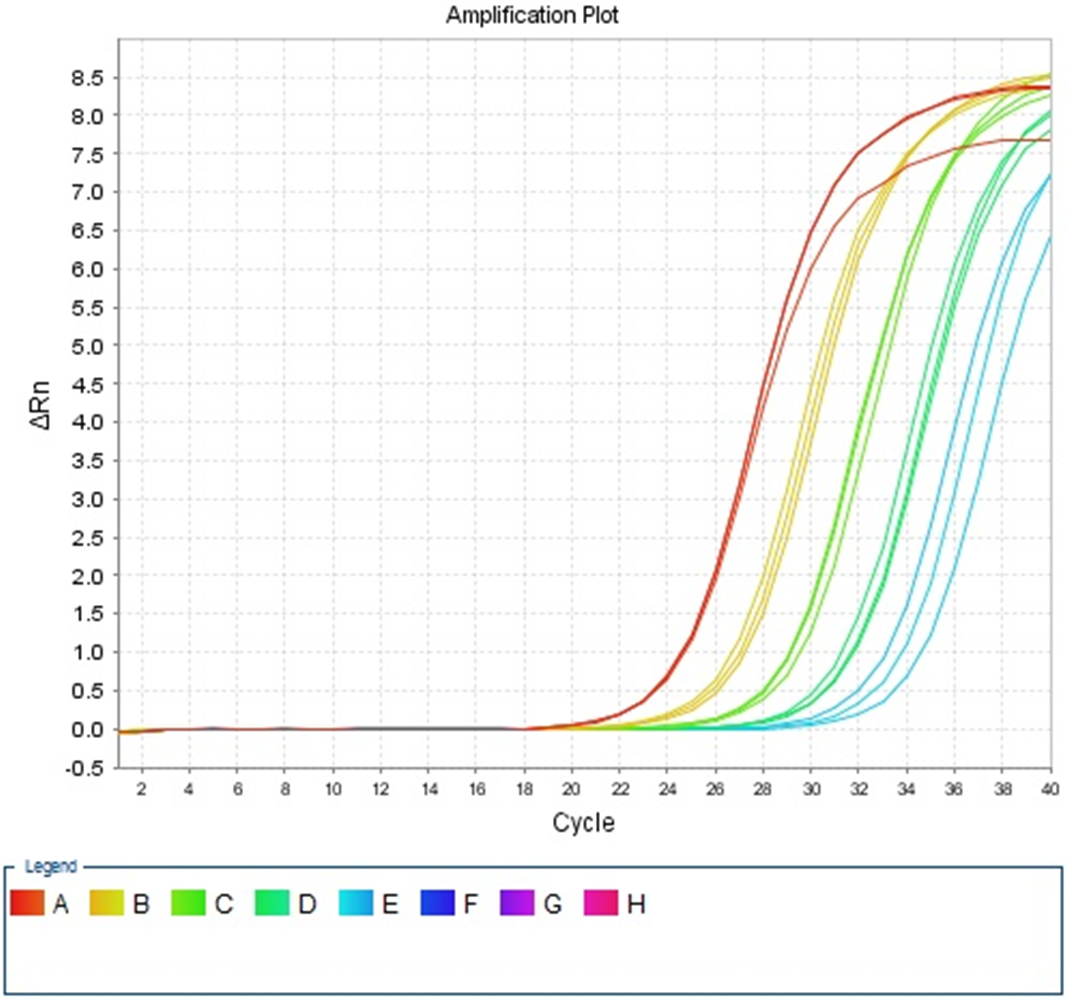** | **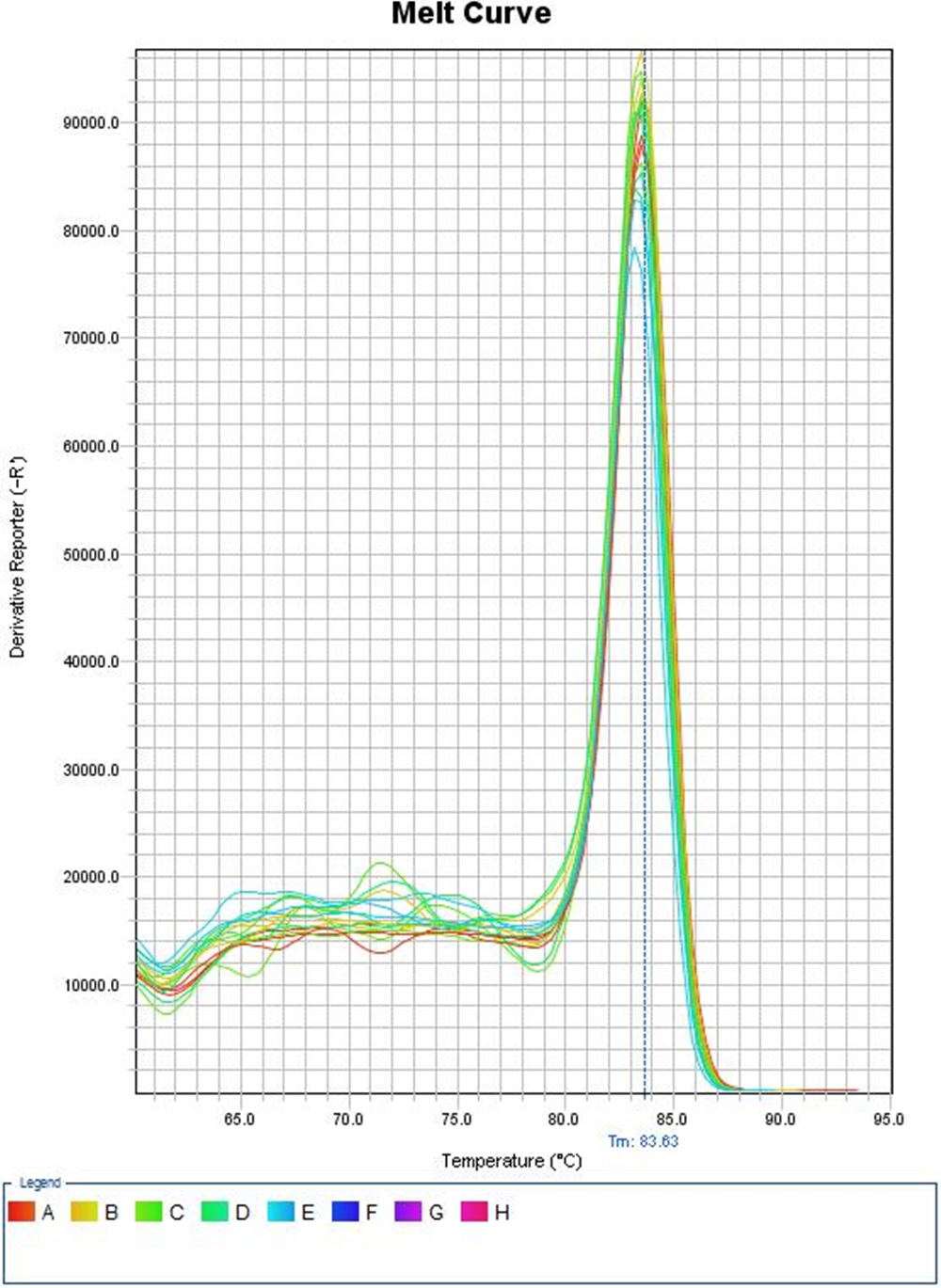** | **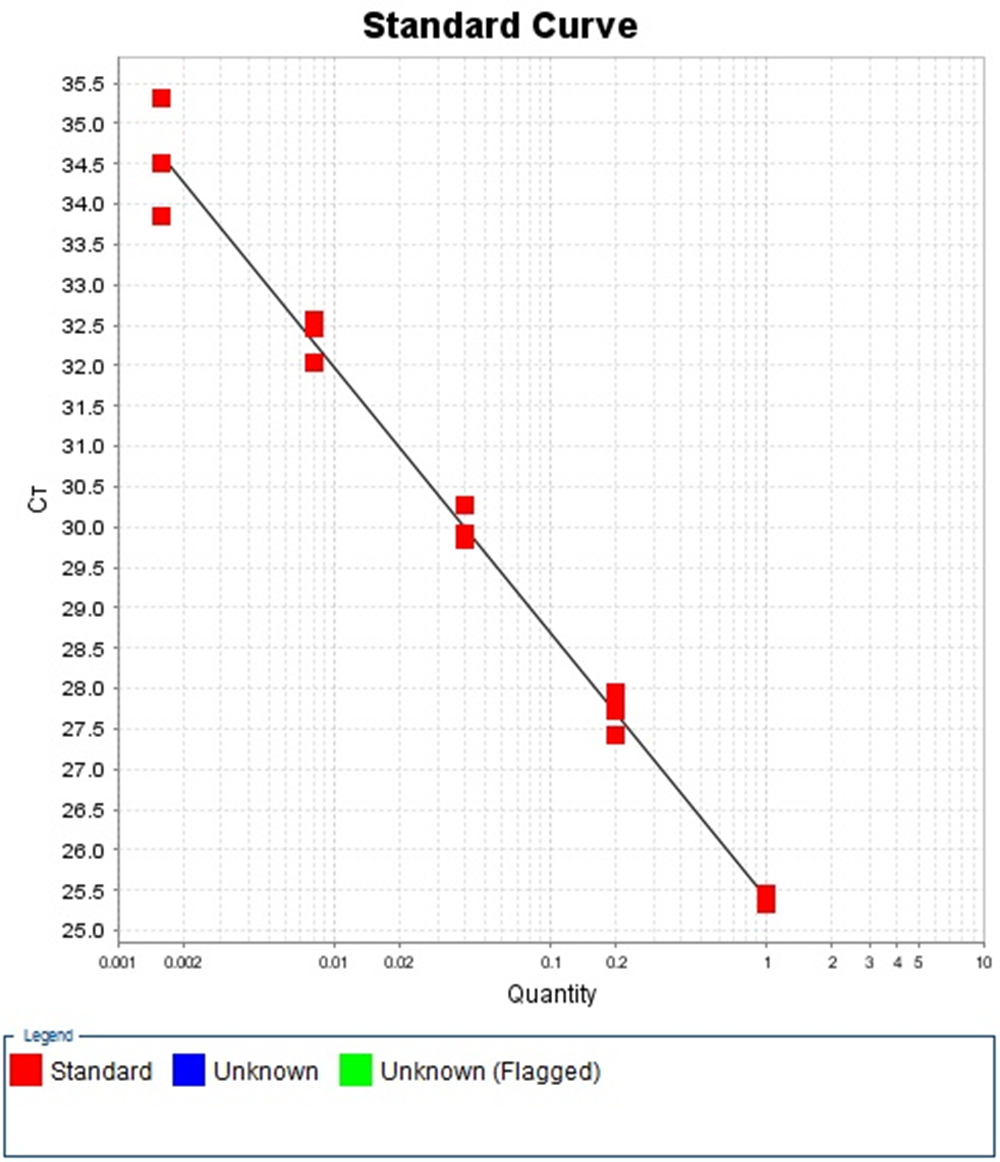** |
