# Supplementary material for: Selection of reference genes for RT‐qPCR normalization in blueberry (Vaccinium corymbosum × angustifolium) under various abiotic stresses
Source: FEBS Open Bio. 2020 Jun 23;10(8):1418–35. doi: 10.1002/2211-5463.12903 (PMC7396441; doi:10.1002/2211-5463.12903)
Supplement: Supplementary file 8 — Table S5. (A) Expression stability ranking of 14 candidate reference genes in leaves of blueberry under abiotic stresses by NormFinder. (B) Expression stability ranking of 14 candidate reference genes in leaves of blueberry under abiotic stresses by NormFinder. [file FEB4-10-1418-s008.doc]

**Table S5 (A). Expression stability ranking of 14 candidate reference genes in leaves of blueberry under abiotic stresses by NormFinder.**

| **Symbol** | **All stresses** | | **NaCl** | | **NaHCO_3_** | | **NaCl + NaHCO_3_** | | **Drought** | | **AlCl_3_** | |
| --- | --- | --- | --- | --- | --- | --- | --- | --- | --- | --- | --- | --- |
|  | **S** ^1^ | Rank | **S** | Rank | **S** | Rank | **S** | Rank | **S** | Rank | **S** | Rank |
| ***ACT*** | 0.352 | 6 | 0.274 | 6 | 0.383 | 10 | 0.442 | 9 | 0.431 | 11 | 0.216 | 6 |
| ***CYP*** | 0.839 | 13 | 1.118 | 13 | 0.660 | 14 | 0.915 | 13 | 0.676 | 14 | 0.494 | 14 |
| ***EF1α*** | 0.475 | 11 | 0.551 | 12 | 0.377 | 9 | 0.696 | 12 | 0.477 | 12 | 0.256 | 7 |
| ***EIF*** | 0.334 | 4 | 0.287 | 8 | 0.243 | 5 | 0.237 | 5 | 0.128 | 3 | 0.304 | 10 |
| ***Fbox*** | 0.342 | 5 | 0.380 | 10 | 0.200 | 3 | 0.213 | 4 | 0.361 | 9 | 0.377 | 12 |
| ***FLD*** | 0.400 | 7 | 0.279 | 7 | 0.168 | 2 | 0.085 | 1 | 0.630 | 13 | 0.186 | 5 |
| ***GAPDH*** | 0.495 | 12 | 0.092 | 1 | 0.588 | 13 | 0.469 | 10 | 0.084 | 1 | 0.114 | 2 |
| ***HIS*** | 0.443 | 10 | 0.167 | 2 | 0.418 | 11 | 0.346 | 8 | 0.428 | 10 | 0.484 | 13 |
| ***PP2A*** | 0.266 | 2 | 0.173 | 3 | 0.349 | 8 | 0.335 | 7 | 0.282 | 8 | 0.172 | 4 |
| ***RP*** | 0.234 | 1 | 0.258 | 5 | 0.228 | 4 | 0.195 | 3 | 0.184 | 6 | 0.028 | 1 |
| ***SAND*** | 1.415 | 14 | 1.748 | 14 | 0.126 | 1 | 1.589 | 14 | 0.134 | 4 | 0.332 | 11 |
| ***TBP*** | 0.401 | 8 | 0.207 | 4 | 0.459 | 12 | 0.552 | 11 | 0.158 | 5 | 0.127 | 3 |
| ***TUB*** | 0.292 | 3 | 0.348 | 9 | 0.313 | 6 | 0.126 | 2 | 0.228 | 7 | 0.266 | 9 |
| ***UBCE*** | 0.420 | 9 | 0.446 | 11 | 0.314 | 7 | 0.319 | 6 | 0.091 | 2 | 0.264 | 8 |

^1^ S: Stability values calculated by NormFinder. The lower S value is, the more stable of the gene has.

**Table S5 (B). Expression stability ranking of 14 candidate reference genes in leaves of blueberry under abiotic stresses by NormFinder.**

| **Symbol** | **All stresses** | | **NaCl** | | **NaHCO_3_** | | **NaCl + NaHCO_3_** | | **Drought** | | **AlCl_3_** | |
| --- | --- | --- | --- | --- | --- | --- | --- | --- | --- | --- | --- | --- |
|  | **S**^1^ | Rank | **S** | Rank | **S** | Rank | **S** | Rank | **S** | Rank | **S** | Rank |
| ***ACT*** | 0.852 | 14 | 0.220 | 5 | 0.405 | 11 | 0.361 | 11 | 1.827 | 14 | 0.511 | 8 |
| ***CYP*** | 0.594 | 13 | 0.688 | 14 | 0.569 | 13 | 0.572 | 14 | 0.061 | 2 | 0.184 | 3 |
| ***EF1α*** | 0.352 | 5 | 0.256 | 8 | 0.309 | 6 | 0.173 | 3 | 0.528 | 11 | 0.073 | 2 |
| ***EIF*** | 0.406 | 7 | 0.238 | 7 | 0.203 | 4 | 0.380 | 13 | 0.221 | 6 | 0.734 | 14 |
| ***Fbox*** | 0.504 | 11 | 0.538 | 12 | 0.156 | 3 | 0.214 | 5 | 0.455 | 9 | 0.574 | 10 |
| ***FLD*** | 0.527 | 12 | 0.361 | 10 | 0.737 | 14 | 0.347 | 10 | 0.557 | 12 | 0.670 | 13 |
| ***GAPDH*** | 0.337 | 4 | 0.148 | 3 | 0.314 | 7 | 0.294 | 8 | 0.421 | 7 | 0.224 | 4 |
| ***HIS*** | 0.268 | 2 | 0.338 | 9 | 0.332 | 8 | 0.176 | 4 | 0.192 | 4 | 0.261 | 5 |
| ***PP2A*** | 0.453 | 8 | 0.064 | 1 | 0.470 | 12 | 0.118 | 2 | 0.204 | 5 | 0.511 | 9 |
| ***RP*** | 0.503 | 10 | 0.437 | 11 | 0.380 | 10 | 0.370 | 12 | 0.688 | 13 | 0.641 | 11 |
| ***SAND*** | 0.389 | 6 | 0.077 | 2 | 0.334 | 9 | 0.282 | 6 | 0.511 | 10 | 0.481 | 7 |
| ***TBP*** | 0.189 | 1 | 0.149 | 4 | 0.284 | 5 | 0.116 | 1 | 0.061 | 2 | 0.072 | 1 |
| ***TUB*** | 0.290 | 3 | 0.225 | 6 | 0.030 | 1 | 0.319 | 9 | 0.053 | 1 | 0.451 | 6 |
| ***UBCE*** | 0.489 | 9 | 0.670 | 13 | 0.092 | 2 | 0.284 | 7 | 0.452 | 8 | 0.655 | 12 |

^1^ S: Stability values calculated by NormFinder. The lower S value is, the more stable of the gene has.
