# Supplementary material for: Selection of reference genes for RT‐qPCR normalization in blueberry (Vaccinium corymbosum × angustifolium) under various abiotic stresses
Source: FEBS Open Bio. 2020 Jun 23;10(8):1418–35. doi: 10.1002/2211-5463.12903 (PMC7396441; doi:10.1002/2211-5463.12903)
Supplement: Supplementary file 9 — Table S6. Expression stability ranking of 14 candidate reference genes in leaves and roots of blueberry under abiotic stresses by BestKeeper. [file FEB4-10-1418-s009.doc]

**Table S6. Expression stability ranking of 14 candidate reference genes in leaves / roots of blueberry under abiotic stresses by BestKeeper.**

| **Symbol** | **all stresses** | | | | | |
| --- | --- | --- | --- | --- | --- | --- |
|  | **Leaves** | | | **Roots** | | |
|  | **r** ^1^ | **SD** ^2^ | **Rank** | **r** | **SD** | **Rank** |
| ***ACT*** | 0.548 | 0.517409833 | 10 | - | - | (14) |
| ***CYP*** | - ^3^ | - | (13) | - | - | (13) |
| ***EF1α*** | - | - | (12) | - | - | (7) |
| ***EIF*** | 0.739 | 0.449253731 | 5 | 0.845 | 0.683208457 | 4 |
| ***Fbox*** | 0.739 | 0.712714889 | 5 | - | - | (11) |
| ***FLD*** | 0.845 | 0.740617655 | 2 | - | - | (12) |
| ***GAPDH*** | - | - | (11) | 0.89 | 0.823365908 | 3 |
| ***HIS*** | 0.88 | 0.644910311 | 1 | 0.942 | 0.716424074 | 2 |
| ***PP2A*** | 0.815 | 0.593129441 | 3 | - | - | (10) |
| ***RP*** | 0.757 | 0.325406226 | 4 | 0.752 | 0.729652148 | 6 |
| ***SAND*** | - | - | (14) | - | - | (8) |
| ***TBP*** | 0.694 | 0.664303847 | 9 | 0.97 | 0.92269396 | 1 |
| ***TUB*** | 0.708 | 0.471110691 | 8 | - | - | (9) |
| ***UBCE*** | 0.711 | 0.449842535 | 7 | 0.806 | 0.511118799 | 5 |

^1^ r: Pearson correlation coefficient calculated by BestKeeper. The higher M value is, the more stable of the gene has.

^2^ SD: The standard deviation calculated by BestKeeper. The value of SD should be less than 1.

^3^ -: The Pearson correlation coefficient which has *P* value more than 0.05 or the value of SD more than 1 was deleted.
