# Supplementary material for: Selection of reference genes for RT‐qPCR normalization in blueberry (Vaccinium corymbosum × angustifolium) under various abiotic stresses
Source: FEBS Open Bio. 2020 Jun 23;10(8):1418–35. doi: 10.1002/2211-5463.12903 (PMC7396441; doi:10.1002/2211-5463.12903)
Supplement: Supplementary file 10 — Table S7. Expression stability comprehensive ranking of 14 candidate reference genes in leaves and roots under abiotic stresses. (A) Expression stability comprehensive ranking of 14 candidate reference genes in leaves under abiotic stresses. (B) Expression stability comprehensive ranking of 14 candidate reference genes in roots under abiotic stresses. [file FEB4-10-1418-s010.doc]

**Table S7 (A). Expression stability comprehensive ranking of 14 candidate reference genes in leaves under** **abiotic stresses.**

| **Method** | **1** | **2** | **3** | **4** | **5** | **6** | **7** | **8** | **9** | **10** | **11** | **12** | **13** | **14** |
| --- | --- | --- | --- | --- | --- | --- | --- | --- | --- | --- | --- | --- | --- | --- |
| **(A) RANKING ORDER LEAVES UNDER ALL STRESS (BETTER-GOOD-AVERAGE)** | | | | | | | | | | | | | | |
| **geNorm** | *PP2A/TBP* |  | *HIS* | *UBCE* | *RP* | *EIF* | *FLD* | *Fbox* | *TUB* | *ACT* | *GAPDH* | *EF1α* | *CYP* | *SAND* |
| **NormFinder** | *RP* | *PP2A* | *TUB* | *EIF* | *Fbox* | *ACT* | *FLD* | *TBP* | *UBCE* | *HIS* | *EF1α* | *GAPDH* | *CYP* | *SAND* |
| **BestKeeper** | *HIS* | *FLD* | *PP2A* | *RP* | *Fbox* | *EIF* | *UBCE* | *TUB* | *TBP* | *ACT* | *GAPDH* | *EF1α* | *CYP* | *SAND* |
| **Comprehensive Ranking** | *PP2A* | *RP* | *HIS* | *EIF* | *FLD* | *Fbox* | *TBP* | *TUB* | *UBCE* | *ACT* | *GAPDH* | *EF1α* | *CYP* | *SAND* |
| **(B) RANKING ORDER LEAVES UNDER NaCl STRESS (BETTER-GOOD-AVERAGE)** | | | | | | | | | | | | | | |
| **geNorm** | *ACT/RP* |  | *GAPDH* | *TBP* | *PP2A* | *HIS* | *FLD* | *TUB* | *EIF* | *Fbox* | *UBCE* | *EF1α* | *CYP* | *SAND* |
| **NormFinder** | *GAPDH* | *HIS* | *PP2A* | *TBP* | *RP* | *ACT* | *FLD* | *EIF* | *TUB* | *Fbox* | *UBCE* | *EF1α* | *CYP* | *SAND* |
| **BestKeeper** | - | - | - | - | - | - | - | - | - | - | - | - | - | - |
| **Comprehensive Ranking** | *GAPDH* | *RP* | *ACT* | *HIS* | *PP2A* | *TBP* | *FLD* | *EIF* | *TUB* | *Fbox* | *UBCE* | *EF1A* | *CYP* | *SAND* |
| **(C) RANKING ORDER LEAVES UNDER NaHCO_3_ STRESS (BETTER-GOOD-AVERAGE)** | | | | | | | | | | | | | | |
| **geNorm** | *EIF/SAND* |  | *ACT* | *TUB* | *RP* | *Fbox* | *EF1α* | *FLD* | *UBCE* | *PP2A* | *HIS* | *TBP* | *GAPDH* | *CYP* |
| **NormFinder** | *SAND* | *FLD* | *Fbox* | *RP* | *EIF* | *TUB* | *UBCE* | *PP2A* | *EF1α* | *ACT* | *HIS* | *TBP* | *GAPDH* | *CYP* |
| **BestKeeper** | - | - | - | - | - | - | - | - | - | - | - | - | - | - |
| **Comprehensive Ranking** | *SAND* | *EIF* | *Fbox* | *RP* | *FLD* | *UBCE* | *ACT* | *TUB* | *EF1A* | *PP2A* | *HIS* | *TBP* | *GAPDH* | *CYP* |
| **(D) RANKING ORDER LEAVES UNDER NaCl + NaHCO_3_ STRESS (BETTER-GOOD-AVERAGE)** | | | | | | | | | | | | | | |
| **geNorm** | *HIS/PP2A* |  | *UBCE* | *RP* | *FLD* | *TUB* | *Fbox* | *EIF* | *TBP* | *GAPDH* | *ACT* | *EF1α* | *CYP* | *SAND* |
| **NormFinder** | *FLD* | *TUB* | *RP* | *Fbox* | *EIF* | *UBCE* | *PP2A* | *HIS* | *ACT* | *GAPDH* | *TBP* | *EF1α* | *CYP* | *SAND* |
| **BestKeeper** | - | - | - | - | - | - | - | - | - | - | - | - | - | - |
| **Comprehensive Ranking** | *FLD* | *RP* | *PP2A* | *TUB* | *HIS* | *UBCE* | *Fbox* | *EIF* | *ACT* | *GAPDH* | *TBP* | *EF1A* | *CYP* | *SAND* |
| **(E) RANKING ORDER LEAVES UNDER DROUGHT STRESS (BETTER-GOOD-AVERAGE)** | | | | | | | | | | | | | | |
| **geNorm** | *GAPDH/UBCE* |  | *TBP* | *SAND* | *EIF* | *RP* | *PP2A* | *TUB* | *Fbox* | *HIS* | *ACT* | *EF1α* | *FLD* | *CYP* |
| **NormFinder** | *GAPDH* | *UBCE* | *EIF* | *SAND* | *TBP* | *RP* | *TUB* | *PP2A* | *Fbox* | *HIS* | *ACT* | *EF1α* | *FLD* | *CYP* |
| **BestKeeper** | - | - | - | - | - | - | - | - | - | - | - | - | - | - |
| **Comprehensive Ranking** | *GAPDH* | *UBCE* | *EIF* | *SAND* | *TBP* | *RP* | *PP2A* | *TUB* | *Fbox* | *HIS* | *ACT* | *EF1A* | *FLD* | *CYP* |
| **(F) RANKING ORDER LEAVES UNDER AlCl_3_ STRESSES (BETTER-GOOD-AVERAGE)** | | | | | | | | | | | | | | |
| **geNorm** | *FLD/PP2A* |  | *RP* | *TBP* | *GAPDH* | *UBCE* | *ACT* | *EIF* | *EF1α* | *TUB* | *SAND* | *Fbox* | *HIS* | *CYP* |
| **NormFinder** | *RP* | *GAPDH* | *TBP* | *PP2A* | *FLD* | *ACT* | *EF1α* | *UBCE* | *TUB* | *EIF* | *SAND* | *Fbox* | *HIS* | *CYP* |
| **BestKeeper** | - | - | - | - | - | - | - | - | - | - | - | - | - | - |
| **Comprehensive Ranking** | *RP* | *PP2A* | *FLD* | *GAPDH* | *TBP* | *ACT* | *UBCE* | *EF1A* | *EIF* | *TUB* | *SAND* | *Fbox* | *HIS* | *CYP* |

-: The Pearson correlation coefficient which has *P* value more than 0.05 or the value of SD more than 1 was deleted.

**Table S7 (B). Expression stability comprehensive ranking of 14 candidate reference genes in roots under abiotic stresses.**

| **Method** | **1** | **2** | **3** | **4** | **5** | **6** | **7** | **8** | **9** | **10** | **11** | **12** | **13** | **14** |
| --- | --- | --- | --- | --- | --- | --- | --- | --- | --- | --- | --- | --- | --- | --- |
| **(A) RANKING ORDER ROOTS UNDER ALL STRESS (BETTER-GOOD-AVERAGE)** | | | | | | | | | | | | | | |
| **geNorm** | *SAND/TBP* |  | *TUB* | *EF1α* | *PP2A* | *HIS* | *GAPDH* | *EIF* | *UBCE* | *RP* | *Fbox* | *FLD* | *CYP* | *ACT* |
| **NormFinder** | *TBP* | *HIS* | *TUB* | *GAPDH* | *EF1α* | *SAND* | *EIF* | *PP2A* | *UBCE* | *RP* | *Fbox* | *FLD* | *CYP* | *ACT* |
| **BestKeeper** | *TBP* | *HIS* | *GAPDH* | *EIF* | *UBCE* | *RP* | *EF1α* | *SAND* | *TUB* | *PP2A* | *Fbox* | *FLD* | *CYP* | *ACT* |
| **Comprehensive Ranking** | *TBP* | *HIS* | *GAPDH* | *TUB* | *SAND* | *EF1α* | *EIF* | *PP2A* | *UBCE* | *RP* | *Fbox* | *FLD* | *CYP* | *ACT* |
| **(B) RANKING ORDER ROOTS UNDER NaCl STRESS (BETTER-GOOD-AVERAGE)** | | | | | | | | | | | | | | |
| **geNorm** | *PP2A/TBP* |  | *SAND* | *GAPDH* | *ACT* | *EIF* | *TUB* | *EF1α* | *FLD* | *HIS* | *RP* | *Fbox* | *UBCE* | *CYP* |
| **NormFinder** | *PP2A* | *SAND* | *GAPDH* | *TBP* | *ACT* | *TUB* | *EIF* | *EF1α* | *HIS* | *FLD* | *RP* | *Fbox* | *UBCE* | *CYP* |
| **BestKeeper** | - | - | - | - | - | - | - | - | - | - | - | - | - | - |
| **Comprehensive Ranking** | *PP2A* | *SAND* | *TBP* | *GAPDH* | *ACT* | *EIF* | *TUB* | *EF1A* | *FLD* | *HIS* | *RP* | *Fbox* | *UBCE* | *CYP* |
| **(C) RANKING ORDER ROOTS UNDER NaHCO_3_ STRESS (BETTER-GOOD-AVERAGE)** | | | | | | | | | | | | | | |
| **geNorm** | *GAPDH/HIS* |  | *UBCE* | *TUB* | *Fbox* | *EIF* | *EF1α* | *SAND* | *TBP* | *RP* | *ACT* | *PP2A* | *CYP* | *FLD* |
| **NormFinder** | *TUB* | *UBCE* | *Fbox* | *EIF* | *TBP* | *EF1α* | *GAPDH* | *HIS* | *SAND* | *RP* | *ACT* | *PP2A* | *CYP* | *FLD* |
| **BestKeeper** | - | - | - | - | - | - | - | - | - | - | - | - | - | - |
| **Comprehensive Ranking** | *TUB* | *UBCE* | *Fbox* | *GAPDH* | *HIS* | *EIF* | *EF1A* | *TBP* | *SAND* | *RP* | *ACT* | *PP2A* | *CYP* | *FLD* |
| **(D) RANKING ORDER ROOTS UNDER NaCl + NaHCO_3_ STRESS (BETTER-GOOD-AVERAGE)** | | | | | | | | | | | | | | |
| **geNorm** | *SAND/TUB* |  | *Fbox* | *PP2A* | *TBP* | *FLD* | *EF1α* | *HIS* | *UBCE* | *GAPDH* | *ACT* | *EIF* | *RP* | *CYP* |
| **NormFinder** | *TBP* | *PP2A* | *EF1α* | *HIS* | *Fbox* | *SAND* | *UBCE* | *GAPDH* | *TUB* | *FLD* | *ACT* | *RP* | *EIF* | *CYP* |
| **BestKeeper** | - | - | - | - | - | - | - | - | - | - | - | - | - | - |
| **Comprehensive Ranking** | *PP2A* | *TBP* | *SAND* | *Fbox* | *EF1A* | *TUB* | *HIS* | *FLD* | *UBCE* | *GAPDH* | *ACT* | *EIF* | *RP* | *CYP* |
| **(E) RANKING ORDER ROOTS UNDER DROUGHT STRESS (BETTER-GOOD-AVERAGE)** | | | | | | | | | | | | | | |
| **geNorm** | *FLD/SAND* |  | *Fbox* | *EF1α* | *PP2A* | *TUB* | *TBP* | *CYP* | *HIS* | *EIF* | *GAPDH* | *UBCE* | *RP* | *ACT* |
| **NormFinder** | *TUB* | *TBP* | *CYP* | *HIS* | *PP2A* | *EIF* | *GAPDH* | *UBCE* | *Fbox* | *SAND* | *EF1α* | *FLD* | *RP* | *ACT* |
| **BestKeeper** | - | - | - | - | - | - | - | - | - | - | - | - | - | - |
| **Comprehensive Ranking** | *TUB* | *TBP* | *PP2A* | *CYP* | *SAND* | *Fbox* | *FLD* | *HIS* | *EF1A* | *EIF* | *GAPDH* | *UBCE* | *RP* | *ACT* |
| **(F) RANKING ORDER ROOTS UNDER AlCl_3_ STRESSES (BETTER-GOOD-AVERAGE)** | | | | | | | | | | | | | | |
| **geNorm** | *PP2A/SAND* |  | *TUB* | *TBP* | *CYP* | *EF1α* | *GAPDH* | *FLD* | *Fbox* | *HIS* | *ACT* | *RP* | *UBCE* | *EIF* |
| **NormFinder** | *TBP* | *EF1α* | *CYP* | *GAPDH* | *HIS* | *TUB* | *SAND* | *ACT* | *PP2A* | *Fbox* | *RP* | *UBCE* | *FLD* | *EIF* |
| **BestKeeper** | - | - | - | - | - | - | - | - | - | - | - | - | - | - |
| **Comprehensive Ranking** | *TBP* | *CYP* | *EF1A* | *SAND* | *TUB* | *PP2A* | *GAPDH* | *HIS* | *ACT* | *Fbox* | *FLD* | *RP* | *UBCE* | *EIF* |

-: The Pearson correlation coefficient which has *P* value more than 0.05 or the value of SD more than 1 was deleted.
