# Supplementary material for: Selection of reference genes for RT‐qPCR normalization in blueberry (Vaccinium corymbosum × angustifolium) under various abiotic stresses
Source: FEBS Open Bio. 2020 Jun 23;10(8):1418–35. doi: 10.1002/2211-5463.12903 (PMC7396441; doi:10.1002/2211-5463.12903)
Supplement: Supplementary file 11 — Data S1. Primer pair annealing locations on their respective transcripts. [file FEB4-10-1418-s011.doc]

**Data S1. The primer pairs location on respective transcript sequence**

Primer sequences were in gray boxes.

***Actin*  Amplicon length 112 bp**

GCTGGCCGTGACCTAACTGATGCCCTCATGAAAATCCTTACGGAGCGTGGGTACTCTTTCACCACCACAGCCGAGCGTGAAATCGTGAGGGACATGAAGGAGAAGCTAGCCTACATAGCCCTGGACTACGAGCAAGAGTTGGAAACTTCCAAGACTAGCTCTTCCGTTGAGAAAAGCTACGAGTTGCCTGATGGACAGGTGATCACCATCGGGGCTGAGCGTTTCCGCTGCCCGGAAGTCCTTTTCCAGCCATCCATGATTGGAATGGAGGCAGCGGGCATCCATGAGACCACATACAATTCCATCATGAAGTGTGACGTGGATATTAGGAAGGACCTTTACGGAAACATCGTTCTCAGTGGTGGGTCAACCATGTTCCCTGGAATTGCTGATAGAATGAGCAAGGAAATAACAGCGTTGGCCCCGAGCAGCATGAAAATAAAGGTGGTGGCTCCACCAGAGAGGAAGTACAGTGTTTGGATCGGAGGATCCATCCTCGCATCCCTCAGTACCTTCCAGCAGATGTGGATTGCAAAGGCAGAGTACGATGAATCTGGACCGTCCATTGTGCACAGGAAATGCTTC

***CYP*  Amplicon length 101 bp**

TTTGACATACTGATCGGAAAAATGAAAGCCGGAAGGATTGTGATGGAGCTATTTGCTGATACCACGCCCAAAACTGCTGAAAACTTCCGTGCCCTCTGCACCGGGGAGAAAGGGATTGGAAAAGCCGGGAAGCCATTGCACTACAAAGGGTCTTCGTTTCACCGCATAATCCCAAACTTCATGTGTCAGGGTGGGGATTTCACGAGGGGTAATGGAACCGGCGGGGAATCGATCTACGGAGAGAAATTCGAAGATGAAAACTTCAAGCTGAGGCATACGGGTCCTGGTATTCTTTCAATGGCAAATGCTGGGGGTAACACTAATGGGTCCCAGTTCTTCATTTGCACCGAGAAGACACCGTGGCTTGATGGAAAGCACGTGGTGTTTGGAAAGGTCGTGGATGGGTATAGCGTGGTTAAGGAGATGGAGAAGGTGGGGTCCGATAGCGGGACGACTTCGGCAACGGTCGTGATCGAGGATTCTGGTGAGGTAACG

***EF1******α*  Amplicon length 147 bp**

GCAAATTTCACGTCTCAGGTTATTATCATGAATCATCCAGGTCAAATTGGAAATGGGTATGCCCCAGTTCTTGACTGCCACACTTCCCACATCGCTGTCAAGTTTGCTGAGATCTTGACCAAGATTGACCGACGATCTGGCAAGGAGATTGAGAAGGAACCCAAGTTCTTGAAGAATGGAGATGCCGGTATGGTGAAGATGATTCCGACCAAGCCTATGGTTGTGGAGACATTCTCCGAGTACCCACCTTTGGGAAGG

***EIF*  Amplicon length 117 bp**

TTTGGGAGGAAAGGTGTTGCCATCAATTTCGTCACAAAAGATGATGAGAGGATGCTGTTTGACATCCAGAAGTTCTACAATGTGGTAGTTGAGGAGCTCCCAGCAAACGTTGCTGATCTCCTT

***F box* Amplicon length 109 bp**

ACCTCACCGGCGGCGGAATTAATTGCCAACAACTTCGATCTTCTTACAGAAATCCTTATACGTTTGCCCTCGAAATCCACAACCCGATTCAAGAGCGTGTCAAAGCATTGGCTCTATATCCTCTCCGACTCCCCCAAATTTGCCAGAAACCCCTCAAGCCGAAACCCTAGACCCACCGTCTCAGGTTTGCATTTCTACATCGACGAAACACTCAACTTCGTCTCCCTTGACGGCCTCCGCGGTAATCTCCCTTCTCTCTCCTTCCTCGATTGTCTTCTTAGAGAACAATCTACTATTAGGGTTACACACTCCTGCAATGGCTTGCTGTTGTGCTATATTAGGCACTTACGTAGTTTTGAACGGAGATATGTTGTTTGTAATGTCACCACAAGGCGATACACAATAATCCCTGAACCTAATGGATCAAATCATGGTCCTGCTTACTTGGCTTTTGATCCTTCCAAATCCCCACACTACAAAGTTTTACTGGTTTGTAGCAGCTTTTATCAAGTTGATGTCTACTGTTCACAGAGTGCATGTTGGAGAGAGGTTTTCCCAAACCAAAAATGTTATGGCCACAGTGTCTTTTGGAATGGAGCAATCCATTGG

***FLD*** **Amplicon length 100 bp**

GGGAGATTTTCTATAATTTTTAGTCGGAAGAATTCAGATCCCAAGTCTACGGCAGTTTTGAGGGTGACATTTTGTGGACAACGAAAGAGGAATCAGGAAGGCTCAAAGCCAAACCAACCGCACTCCANNNNNNAATTACTATTCCAACAGCTTCAGTCCCATTTTAATCAGCAGCAGGAGTTTCATGTTTACGCTTTGCTATCAAGGGAACAGGCGCTTGAGCTGAGGGAAGTAAGAGGGGGTGATGATATGAGATTGAATCACCTATGTGAAAAGCTTGGAGTGAAGCTGGTTGGGAGAAAAGGTCTGGGGCCTACCGCGGATTCTGTTATTGCTTCCATCAAGGCCGAGAGGGGAAACCGCAAGTCTGCTTCAACTTCTTTGTTACTTAAATCTGGAACATCCAAGCCAAGAGCAGCTATTTTGAAGAAAAAGTTTGTTAGGAAGGCTAAA

***GAPDH*  Amplicon length 105 bp**

AAGAAGATCAAGATCGGGATCAATGGCTTTGGAAGGATCGGTCGTCTTGTAGCTAGGGTTGCTCTCCAGAGAGACGATGTTGAACTCGTTGCTGTTAACGATCCCTTCATTACCACTGATTACATGACATATATGTTCAAGTATGACAGTGTTCACGGCCAGTGGAAGCATCATGAGCTTAAGGTTAAGGACTCTAAGACCCTTCTTTTCGGTGAGAAACCAGTTACTGTTTTTGGCCACAGGAACCCGGAAGAAATCCCTTGGGGTGAAACCGGAGCTGAGTTTGTTGTTGAGTCTACTGGAGTGTTCACTGACAAGGACAAGGCTGCTGCCCACTTAAAGGGTGGTGCAAAGAAGGTGGTCATCTCTGCCCCAAGTAAGGATGCTCCTATGTTTGTTATGGGTGTTAATGAGAAAGAATACAAGCCCGATCTTCACATTGTCTCCAATGCTAGCTGCACTACTAACTGTCTTGCTCCGCTTGCCAAGGTCATCAATGATAGGTTTGGCATTGTTGAGGGTCTTATGACAACCGTTCACTCCATCACGGCCACTCAGAAGACTGTTGATGGACCATCAAGC

***HIS*  Amplicon length 127 bp**

TTATTACAGAAGAAGAGTAGTTTCCTTGCATTCGAAAGCGATCTCGGATTCTCCAATTCAATGGCTCGTACCAAGCAAACTGCTCGTAAGTCTACTGGAGGCAAGGCGCCAAGGAAGCAGCTTGCTACCAAGGCTGCTCGGAAGTCTGCCCCTACTACTGGAGGAGTCAAGAAGCCCCACAGATACAGACCTGGAACTGTTGCTCTTCGTGAAATTCGCAAGTATCAGAAAAGTACTGAACTCCTAATCAGGAAGTTACCATTCCAGAGGCTTGTTCGTGAGATTGCTCAAGATTTCAAGACTGACCTGAGGTTCCAGAGCCATGCAGTCTTGGCACTGCAGGAGGCAGCAGAGGCATACCTTGTTGGGTTGTTTGAAGATACTAATCTGTGTGCAATTCACGCCAAGCGTGTCACTATTATGCCCAAGGATATTCAGCTTGCCAGGAGAATTAGGGGGGAGAGGGCT

***PP2A*  Amplicon length 103 bp**

ATGATCAAACAGATACTTAATAGGCTCCCAAGGAAGCCGTCTAAGTCAGCAGAAAATCGCGACGGGGGAACCTCTACCTCCTCTTCAAATGCTTCTTCCGGTTTACGAAGCAGTGATCTAGCAAGCAATCCGTATGGGAACTGGAATGCTGCATCTCTTCCGGGTCAAAATCCTACTTCAAATTCAGGAATAAGTCATGGAAATAAGGTCCCTCAAGCTTTCAACTCAAAGCCAAATGGCAATTCAACAGTTTCCTCCTATGAGTTATTGCCTAGTTTTAGAGATGTTTCAAACTCTGAGAAGCAGAACTTGTTCATCAGAAAGCTGAACCTGTGCTGTGTCGTGTTTGACTTTTCTGACCCAGCAAAGAATTTGAAAGAAAAGGAAATCAAGCGGCAGACTTTGGTAGAGCTTGTTGATTATGTTACTTCAGCTGATGGGAAATTCGCAGAGCCTGTTGTGCAAGAAATGGTGAAGATGGTGTCCATAAACTTGTTCCGGACACTCACTCCTCAGCCTCGTGAGAACAAAGTTTTAGAAGCAGTTGATATGGAGGAGGATGAGCCAATGATGGAACCAGCATGGCCTCATCTGCAAATTGTATATGAATTCTTCCTGAGATTCGTGGCATCACCAGGGATGGATGCAAAATTGGCTAAGCGCTATGTTGATCACTCTTTTGTCGTAAGGTTGTTGGATCTTTTCGATTCCGAGGATCCTAGAGAAAGGGAATACTCGAAAACTGTTTTACACCGGATTTATGGAAAGTTTATGGTGCACCGTCCATTTATTAGGAAAACAATCAACAACATATTTTATCGTTTCATTTTTGAAACTGAGAAGCATAACGGAATTGCAGAGCTGTTAGAAATATTGGGCAGTATAATTAACGGATTTGCTTTGCCACTGAAAGAAGAGCACAAACTCTTCCTTGTACGTGCACTGATTCCACTTCACAAACCGAAATGCATAGCGATGTACCATCAGCAGTTATCATATTGCATCACACAATTTGTGGAGAAAGACTGTAAGCTTGCTGACACTGTTATAAGGGGCTTATTACGGTACTGGCCAATCACAAATAGTTCAAAGGAGGTCATGTTTTTAGGTGAGCTGGAGGAGGTTTTAGAAGCGACTCAGCCTCCGGAGTTCCAGCGTTGTATGGTACCCTTATTCCGCCGCATCGCTCGTTGCTTGAGCAGTTCACACTTCCAAGTGGCTGAAAGGGCTCTGTTCTTATGGAACAACGATCACATTGACAACTTAATCAAACAAAATCGGAAGGTTATTTTGCCAATTATATTCCCGGCTTTGGAAAAAAATGCAACAAAACACTGGAACCAGGCAGTTCAGAGCTTGACACTAAACGTCCGCAAGATCTTCTCTGATCTTGATTCGGAGCTATTTGAGGAGTGCTTACTTAAATTTCAAGAAGATAAAGCCCAAGAGGAAGAGATTGAGTTGAAACGTGAATCCGCATGGAAACGCTTAGAGGAGATTGCAGCCATGAAATCTGCG

***RP*  Amplicon length 142 bp**

ATGGAGGCGATGGAGGACGATACGCAAGGGCAGGAGCAGCAACAGCAGAGGCCGCAGCAAGACATGGATTTGAACGGGGAGCCCTACAGCCGGTGCCTGAGCAGCTACGTCGACGAAGGTAGCACCGAGAGTCACAGATACTACCTGTCTCGGAGAACTCTCCTGGAGATGCTCAGAGACCGTGGCTACTCCGTTCCCACCGCAGAAATCGAACTCTCTCTTCAGGATTTCAGGTCCATTCACGGCCAAACACCGGACATCGAACGACTAAAGATCTCCGCTTCTCGCCGATCCAACCCCGATGATAAGATTCTTGTAATTTTCTGTGGACCAGGTGTGGTAAAAGTAAATGTCATTCGCATCATTGGAACTATGATAGGTAACAAAGACTCTTTGGGTCGGCTGATATTAGTAGTGCAAAATCGAGTGACAAACCAGGCTCTGAAAGCTATGGACCTTTTTAGCTTCAAGGTGGAAGTTTTCCAAATTACGGAATTGCTTATTAACATCACAAAGCATGCGTTAAAGCCAAAGCACCGTGTACTGACTGATGCAGAGAAAAAGAAGCTCTTGAAGAAGTTTAGTTTGGAGGAAAAACAGCTTCCTCGGATGTTACAGAAAGATGCAATTTCACGATATTATGGACTGGAGAAGGGGCAAGTGGTGAAAGTTACTTACAGCGGCGACATCACCGAGTCGCATGTCACTTACCGCTGCGTTTGG

***SAND*  Amplicon length 101 bp**

TCCGATTCGGAATCTCCAACGTCCTCCGACGATTCCACGTCCCAAAACCCTAATCCAAACCCTCACCCGAATTCCACTTCAATTGACCAGTCCCTCGACGCCATCGAGGACCAATTAACCTCTATCGCACTCGCCCAACCTAACGACCTTGCATCCGATAATCCTGCCTCCTCCTCCGAAGAGGAAGAAGTGTTCAAGGATGCTCCCAATGGTTCTGTATTGGAAGAAGAAAACCAGAAGGAGAGAAATGAAGGTGAGATAGTAGAAGAGGAGGTAGGAGTTGTTGCTGCGGAAGAGGAGGTTAGGGAGGAGGGGAGGTTGGGTTGGAGGAGGGTGAATTCGGAGGTGGAAGTGGACCGCTTGTCGAGCCCGAGCAGCAGTGGGTATGCGGGTGAGAGAGGGAGCAGTGGTGGTGCCAGTACTGCTAGTGGAATTGAGGAAATTGGTGAAGATCGAGGTTTTGATGGGAATGGGAGGGGTGAGTTTTTTGATGGAGCTTCAGATTCTCAGGCGCCGCCGTGGGTTCCGGGAAAGCGGCATGTCGATGAAGATGATGCTTCTGTTTCGTGGAGGAAAAGGAAAAAGCATTTCTTTATTTTGAGTCACTCAGGCAAACCTATATATTCCAGGTATGGAGATGAACATAAGTTAGCAGGTTTTTCAGCAACTTTGCAAGCAATCATTTCGTTTGTGGAGAATGGGGGAGATCGTGTTAAATTGGTAAGGGCAGGCAAACACCAGGTGGTTTTTCTTGTGAAAGGGCCAATCTACTTGGTTTGCATAAGCTGTACGGAAGAGCCTTACGAGTCGTTAAGGGGGCAGTTGGAGCTTATTTATGGCCAGATGATACTTATTCTTACAAAGTCTGTAAATAGATGTTTTGAGAAGAATCCAAAGTTTGATATGACACCTTTGCTTGGAGGAACGGATGTTGTCTTCTCTTCTCTCGTCCATTCTTTCAGTTGGAACCCGGCCACTTTTCTTCATGCATATACCTGTCTTCCCCTTGCTTATGTAACAAGACAAGCCGCAGGTGCTATATTGCATGACGTAGCTGATTCTGGTGTCCTCTTTGCAATATTAATGTGTAAACACAAGGTAGTCAGTCTTGTTGGTGCACAAAAAGCATCTCTTCATCCTGATGATATGCTCCTACTTTCCAATTTTGTTACGTCATCTGAATCATTTAGGACATCTGAATCTTTCTCACCAATTTGCCTGCCAAGATACAATCCCATGGCATTTTTGTATGCTTATGTCCATTATCTTGATGTGGACACCTACTTAATGTTGCTTACTACCAGTTCAGATGCCTTCTATCATCTAAAAGATTGCAGGATTCGTATCGAAATGGTCCTTCTGAAGTCAAACGTTCTTAGTGAAGTTCAGAGATCCATGCTGGATGGTGGCATGCGCGTTGAGGATTTGCCCGTTGATCCATCTCCTCGTTCTGAATTTTCATCACCTCATTTAGGTCAGTCCCGAATTGCGACAGACTCGCCGGATAGATTCCGAGAAGCATTTGCTGGTATTGGCGGTCCTGCTGGACTTTGGCATTTCATATACCGTAGTATTTTCCTGGACCAATATGTTTCTTCCGAGTTCGCATCACCGATCAACAGTTCGCAACAGCAGAAAAGATTGTATAGAGCTTACCAAAAGCTTTATGCTTCTATGCATGATAAAGTAATTGGACCCCACAAAACTCAGTTCAGAAGAGATGAGAACTATGTTTTACTCTGCTGGGTCACCCCGGACTTCGAACTCTATGCGGCATTTGATCCTCTTGCAGACAAGGCTTTGGCAATAAGTACTTGCAACCGGGTGTGTCAATGGGTGAAGGATGTGGAAAATGAAATTTTCCTGTTGGGAGCTAGCCCCTTTTCATGG

***TBP*  Amplicon length 108 bp**

ATGGCAGATCTATTGGTGGAAGGGAGCCAGAGCCAACCGGTGGATCTTTCTAAGCATCCTTCTGGAATAGTTCCAACTCTCCAGAACATTGTGTCAACAGTGAACTTAGACTGCAAGTTGGAACTTAAGGCCATTGCACTTCAAGCTCGAAATGCAGAATACAATCCCAAGCGTTTTGCTGCTGTAATTATGAGGATAAGAGAACCAAAAACAACAGCATTAATTTTTGCTTCTGGAAAGATGGTTTGTACTGGAGCGAAGAGTGAACAACAGTCAAAACTGGCAGCACGGAAGTATGCTCGAATCATACAAAAGCTTGGTTTTCCAGCTAAATTCAAGGATTTCAAAATTCAGAACATAGTTGGCTCATGCGATGTTAAATTTCCAATTAGACTTGAAGGCCTTGCATACTCCCATGGTGCTTTTTCAAGTTATGAACCAGAGCTGTTCCCTGGATTGATATATCGGATGAAACAACCAAAAATCGTGCTGCTTATATTTGTTTCTGGGAAAATTGTTCTAACTGGAGCGAAGGTGAGAGATGAGACTTATACTGCCTTCGAGAATATATACCCAGTTCTCACCGAGTTCAGAAAAGTTCAGCAA

***TUB*  Amplicon length 101 bp**

GGCGACTCCGATCTCCAGCTTGAGCGTGTCAATGTCTACTACAACGAGGCCAGTTGCGGCCGGTTCGTCCCGCGTGCCGTCCTCATGGACCTCGAGCCCGGTACCATGGACAGCGTCCGCTCCGGCCCCTACGGCCAGATCTTCCGCCCCGATAACTTCGTGTTTGGACAGTCTGGTGCTGGGAATAATTGGGCGAAAGGGCATTATACCGAGGGCGCCGAGCTTATTGATTCGGTTCTCGATGTCGTTCGAAAAGAGGCTGAAAATTGTGACTGCCTACAAGGGTTTCAGGTGTGCCATTCTCTGGGAGGAGGGACAGGATCGGGGATGGGGACGCTGTTGATATCGAAGATAAGGGAGGAGTATCCGGACCGAATGATGCTGACTTTCTCTGTGTTTCCATCACCAAAGGTGTCCGATACTGTGGTAGAACCATACAACGCTACACTCTCCGTCCACCAGCTTGTTGAGAATGCTGATGAGTGTATGGTTTTGGACAACGAGGCACTCTATGATATCTGCTTCCGTACTCTCAAGCTCACTACTCCCAGCTTTGGAGATCTGAACCACTTGATTTCTGCCACCATGAGTGGTGTGACTTGCTGCCTTCGCTTTCCCGGTCAACTCAACTCAGACCTTCGCAAGCTTGCAGTCAACCTCATCCCATTCCCACGACTCCACTTCTTCATGGTCGGCTTTGCACCGCTCACGTCACGTGGCTCCCAGCAATACCGCGCCCTCACCGTTCCCGAACTCACCCAACAGATGTGGGACTCCAAGAACATGATGTGTGCCGCTGATCCCCGTCACGGTCGCTACCTCACGGCATCAGCAATGTTCCGTGGAAAAATGAGCACGAAGGAGGTGGATGAACAGATGATAAACGTGCAGAACAAGAACTCGTCGTACTTTGTTGAGTGGATCCCCAACAATGTGAAATCAACTGTTTGTGACATCCCTCCAACGGGTCTCAAAATGGCTTCGACGTTTATTGGGAACTCTACTTCCATACAAGAGATGTTTAGGAGGGTGAGTGAACAGTTCACGGCTATGTTCCGTAGGAAGGCTTTCTTGCATTGGTACACAGGAGAGGGGATGGACGAGATGGAGTTTACCGAGGCAGAG

***UBCE*  Amplicon length 101 bp**

ATGGCGTCGAAACGGATATTGAAGGAGCTCAAGGATTTGCAGAAGGACCCTCCGACATCGTGCAGTGCCGGTCCTGTAGCCGAAGACATGTTCCATTGGCAAGCAACAATTATGGGACCTACCGATAGCCCTTATGCAGGAGGCGTGTTTCTGGTTTCTATTCATTTTCCTCCAGATTATCCTTTTAAGCCCCCGAAGGTAGCATTCAGGACCAAGGTCTTCCACCCAAATATTAATAGCAATGGAAGTATTTGCCTTGACATTCTGAAAGAGCAATGGAGTCCAGCATTAACCATTTCCAAGGTCTTATTATCCATCTGCTCCCTCCTGACGGACCCAAACCCCGATGATCCTCTTGTGCCAGAAATAGCTCACATGTACAAGACCGACAGGGCCAAATATGAGGCCACTGCTCGCAGTTGGACGCAGAAATACGCCATGGGA
